# Towards unified AI-driven fracture mechanics: the extended deep energy method (XDEM)

**DOI:** 10.1038/s41467-026-76748-1

**Published:** 2026-08-15

**Authors:** Yizheng Wang, Yuzhou Lin, Somdatta Goswami, Luyang Zhao, Huadong Zhang, Jinshuai Bai, Cosmin Anitescu, Mohammad Sadegh Eshaghi, Xiaoying Zhuang, Timon Rabczuk, Yinghua Liu

**Affiliations:** 1 Department of Engineering Mechanics, Tsinghua University, Beijing, China; 2 Department of Civil and Systems Engineering, Johns Hopkins University, Baltimore, MD USA; 3 Department of Mechanical and Materials Engineering, Western University, London, ON Canada; 4 Institute of Structural Mechanics, Bauhaus-Universität Weimar, Weimar, Germany; 5 Institute of Photonics, Department of Mathematics and Physics, Leibniz University Hannover, Hannover, Germany

**Keywords:** Mechanical engineering, Computational science

## Abstract

Physics-Informed Neural Networks (PINNs) have recently emerged as powerful tools for solving partial differential equations (PDEs), with the Deep Energy Method (DEM) proving especially effective in fracture mechanics due to its energy-based formulation. Despite these advances, existing DEM approaches require dense collocation near cracks, face stability challenges, and typically treat discrete and continuous fracture models separately. To overcome these limitations, we introduce the Extended Deep Energy Method (XDEM), a unified deep learning framework that incorporates both displacement discontinuities and crack-tip asymptotics in the discrete setting, while flexibly coupling displacement and phase fields in the continuous setting. This integration enables accurate fracture predictions using uniformly distributed, relatively sparse collocation points. Validation across benchmark problems including stress intensity factor evaluation, straight and kinked crack growth, and complex crack initiation demonstrates that XDEM consistently outperforms standard DEM in accuracy and efficiency. By bridging discrete and phase-field models within a single framework, XDEM establishes a robust foundation for applying AI to fracture mechanics and opens new avenues for predictive modeling in engineering and materials science.

## Abstract (web summary)

This study presents the Extended Deep Energy Method (XDEM), an AI approach for predicting material fracture, opening new possibilities for safer engineering and more resilient materials.

## Introduction

The accurate modeling of the failure of materials and, thereby, structures has long been recognized as a grand challenge in mechanics, shaping decades of research in engineering and materials science. Fracture is a primary driver of material failure, and the accurate simulation of crack initiation and propagation remains a central problem. Classical computational approaches to fracture fall broadly into two categories: discrete fracture models and continuous damage models. Discrete approaches, including the Virtual Crack Closure Technique (VCCT)^1^, the Cohesive Zone Method (CZM)^2,3^, and extended finite element methods (XFEM^4^/XIGA^5,6^) offer computational efficiency but require explicit crack representation and fracture criteria, making them difficult to apply in problems with complex crack networks or three-dimensional geometries. Continuous approaches, such as the phase-field method^7–11^ and peridynamics^12^, provide a more flexible description by allowing cracks to nucleate and evolve without any tracking. However, this flexibility comes at the cost of significantly higher computational expense, primarily due to the need for fine spatial discretization to resolve crack features accurately.

It is important to note that these modeling frameworks-whether discrete or continuous-ultimately lead to governing partial differential equations (PDEs) that must be solved numerically. Traditional numerical schemes such as the finite element method (FEM), finite difference method (FDM), or meshfree methods have long been the workhorses for these PDEs. Recently, a new class of solvers based on machine learning, particularly Physics-Informed Neural Networks (PINNs)^13,14^, has emerged as an alternative paradigm for solving such equations. PINNs and their variational counterpart, the Deep Energy Method (DEM), embed the underlying physical laws directly into the neural network training objective, enabling the solution of PDEs without requiring explicit meshing or labeled data. The DEM formulation is particularly well suited for fracture mechanics, where the governing equations arise naturally from variational energy principles^15^. DEM has been successfully applied to both discrete and continuous fracture models, yielding several representative advances. In discrete formulations, Zhao et al. employed DEM to simulate crack propagation^16^, though their approach required additional collocation points along the evolving crack path. Chen et al.^17^ combined strong-form PINNs with asymptotic fracture solutions to model fatigue crack growth, yet did not exploit the variational DEM framework. Chen et al.^17^ applied the PINNs (employing the PDE in its strong form) combined with asymptotic fracture solutions to simulate fatigue crack growth. For continuous models, Goswami et al. pioneered the use of DEM for phase-field fracture, demonstrating its effectiveness for both second-order^15^ and fourth-order formulations^18^. However, accurate modeling of shear-dominated (mode-II) failure remained challenging. Zheng^19^ proposed a FEM-inspired approach, where the displacement field and the phase field at the nodes of the mesh were predicted by neural networks and subsequently interpolated with shape functions to construct displacement and phase fields. However, this method required mesh refinement along the crack path, similar to FEM. Building on this idea, Manav et al.^20^ conducted a more systematic study, applying DEM to crack nucleation, propagation, kinking, branching, and coalescence. Compared with traditional numerical solvers like FEM and isogeometric analysis (IGA), a key advantage of DEM is that it allows larger load increments^15^, enabling accelerated simulation of crack propagation. Despite these advances, existing DEM approaches to fracture mechanics have treated discrete and continuous models independently. Since each model has its own strengths, this separation limits their overall effectiveness: discrete models offer higher computational efficiency and are advantageous for relatively simple crack growth problems, while continuous phase-field models do not require explicit fracture criteria and are better suited for complex crack patterns and 3D systems. Moreover, existing DEM frameworks typically require refined collocation near crack tips to accurately capture the highly localized fields^15,16,18,20^, which in turn demands a priori knowledge of the crack path or the development of efficient adaptive refinement schemes^18^.

**Fig. 1:**
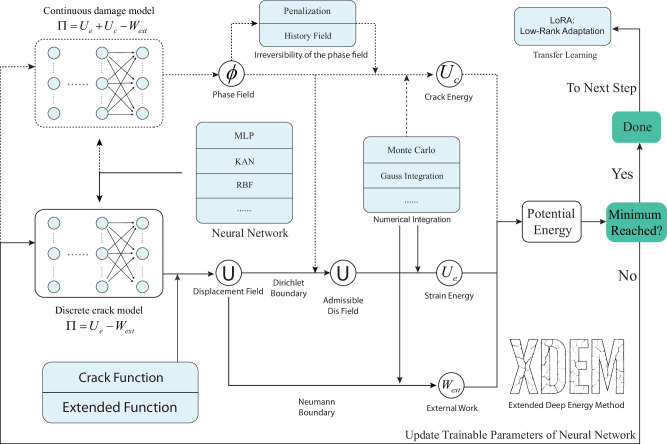
Schematic illustration of the extended deep energy method (XDEM): XDEM consists of discrete and continuous models. The continuous formulation is indicated by dashed lines.

To address these limitations, we propose the Extended Deep Energy Method (XDEM) (Fig. 1), a unified AI framework that bridges discrete and continuous formulations of fracture mechanics. In the discrete regime, XDEM introduces a crack function to represent displacement discontinuities and an extended function to capture near-tip asymptotic fields. In the continuous phase-field regime, XDEM decouples displacement and damage evolution through flexible neural architectures, enforcing irreversibility via penalization or history-field strategies. Together, these developments eliminate the need for problem-specific collocation refinement, while substantially enhancing accuracy, robustness, and computational efficiency. Specifically, XDEM establishes a unified DEM-based framework applicable to both discrete and continuous fracture models. The introduced crack and extended functions enable accurate stress intensity factor identification using sparse, uniformly distributed collocation points. The versatility of XDEM is demonstrated through benchmark studies involving stress intensity factor evaluation, straight, kinked, and branching crack propagation, and crack initiation.

## Results

This section presents the performance of the proposed Extended Deep Energy Method (XDEM) in modeling fracture evolution under varying loading and boundary conditions. XDEM is presented in two forms: discrete (XDEM-D) and continuous (XDEM-C). The results discussed in the main text pertain to XDEM-D, while results for XDEM-C are provided in the Supplementary Note 2.2. The results are organized into two parts: (i) prediction of stress intensity factors (SIFs), which quantify the near-tip stress fields, and (ii) simulation of crack path propagation under different fracture modes (modes I–III).

### Stress intensity factor

The stress intensity factor is a fundamental quantity in linear elastic fracture mechanics, characterizing the intensity of the stress field near the crack tip. We begin by evaluating the ability of XDEM to accurately predict SIFs. Three representative cases: mode I (opening), mode II (sliding), and mode III (tearing) are considered, along with mixed-mode loading conditions. Since the individual modes I–III cracks can be regarded as special cases of mixed-mode fracture, we focus here on the mixed-mode configuration, while detailed results for the pure modes are provided in Supplementary Note 2.1.

**Fig. 2:**
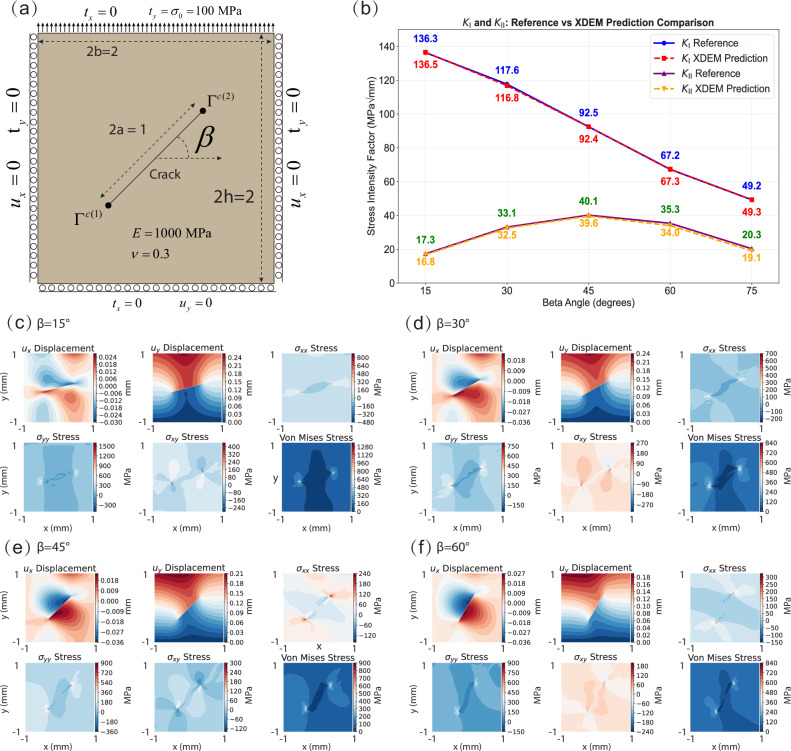
Mixed-mode crack problem. **a** geometry, material properties, and boundary conditions; **b** comparison of XDEM-predicted SIFs (*K*_I_ and *K*_II_) with FEM reference solutions for *a* = 0.5 and *σ*_0_ = 100 MPa at different crack inclination angles *β*. **c–f** Displacement and stress contours predicted by XDEM for different crack angles *β* in the mixed-mode crack problem.

The mixed-mode problem involves a rectangular plate of length 2*b* = 2 and height 2*h* = 2, containing a central crack of length 2*a* = 1 inclined at an angle *β* (see Fig. 2a). The material parameters are *E* = 1000 MPa and Poisson’s ratio *ν* = 0.3, under plane strain conditions. A uniform tensile stress *σ*_0_ = 100 MPa is applied on the top boundary, while the left and right edges are constrained in the *x*-direction (*u*_*x*_ = 0) and the bottom boundary in the *y*-direction (*u*_*y*_ = 0). The displacement fields used in the XDEM formulation are defined in Supplementary Note 2.1.4. Figure 2b compares the XDEM-predicted mode-I and mode-II stress intensity factors (*K*_I_ and *K*_II_) with finite element method (FEM) reference solutions for crack inclination angles *β* ∈ 15°, 30°, 45°, 60°, 75°. XDEM shows excellent agreement across all orientations, accurately capturing both components of the stress intensity factor. The SIFs are computed using the interaction integral method, as described in Supplementary Note 3.8.

These results demonstrate that XDEM reliably captures near-tip stress fields-a key prerequisite for accurate crack propagation modeling, which is explored further in Supplementary Note 2.2. Training parameters are consistent with the mode-I case, using 100 × 100 uniformly distributed collocation points. The displacement and stress contours for various crack angles (Fig. 2c–f) clearly exhibit the expected discontinuities across the crack surface, underscoring the effectiveness of the crack function in representing displacement jumps within the neural architecture.

To examine the sensitivity of XDEM to collocation density, Table 1 summarizes its accuracy and computational efficiency across different point distributions. The results indicate that, for most crack angles, a moderate resolution of 30 × 30 points suffices to achieve high accuracy. At very low point densities, however, integration inaccuracies during training can occasionally lead to spurious stress fields or non-physical fracture patterns (see Supplementary Note 3.1). Such effects can be mitigated by employing early stopping criteria or by modestly increasing the collocation resolution.

**Table 1:** Accuracy and efficiency of XDEM for the mixed-mode crack problem

Angle *β*	Points	*K*_*I*_ (Ref., XDEM)	Rel. error	*K*_*II*_ (Ref., XDEM)	Rel. error	Epochs, Time (s)
15°	30 × 30	136.3, 132.9	2.52%	17.26, 17.63	2.19%	4000, 117
	50 × 50	136.3, 138.2	1.39%	17.26, 18.73	8.51%	2900, 83.9
	80 × 80	136.3, 135.9	**0.28%**	17.26, 17.35	**0.52%**	2600, 74.8
30°	30 × 30	117.6, 112.9	3.96%	33.10, 32.28	2.47%	4400, 127
	50 × 50	117.6, 115.0	2.18%	33.10, 32.44	2.00%	2900, 83.9
	80 × 80	117.6, 118.3	**0.62%**	33.10, 33.22	**0.37%**	5500, 158
45°	30 × 30	92.45, 93.61	1.25%	40.15, 41.45	3.25%	4000, 116
	50 × 50	92.45, 93.86	1.52%	40.15, 41.19	2.60%	3600, 104
	80 × 80	92.45, 93.23	**0.85%**	40.15, 39.58	**1.43%**	3300, 95.2
60°	30 × 30	67.20, 69.80	3.87%	35.32, 37.09	5.02%	3500, 101
	50 × 50	67.20, 69.02	2.71%	35.32, 35.50	5.15%	4800, 139
	80 × 80	67.20, 66.63	**0.84%**	35.32, 34.94	**1.08%**	8300, 239
75°	30 × 30	49.23, 52.03	5.70%	20.30, 19.19	5.48%	4400, 127
	50 × 50	49.23, 51.09	3.76%	20.30, 19.48	4.03%	6500, 188
	80 × 80	49.23, 49.63	**0.81%**	20.30, 19.95	**1.72%**	9700, 279

The results are presented for different crack angles *β* and collocation point densities, using FEM solutions as the reference. Bold values indicate the convergence trend of XDEM with increasing collocation point density.

**Fig. 3:**
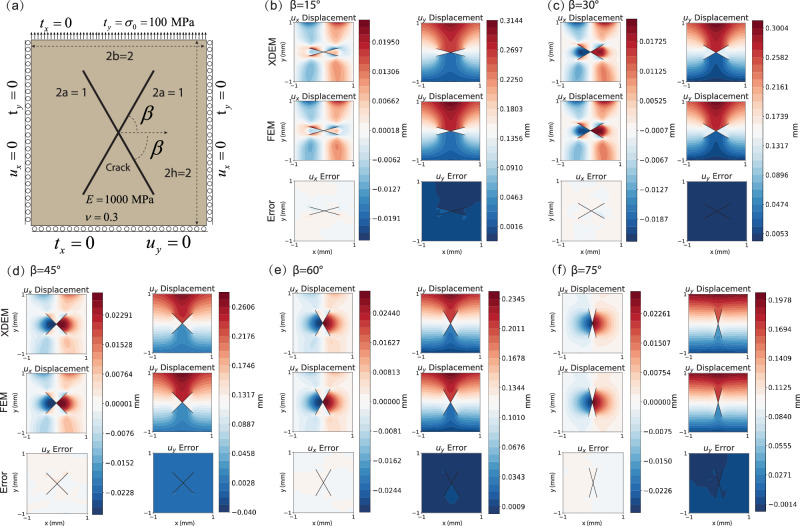
Displacement contours predicted by XDEM and FEM (reference solution) for intersecting cracks. **a** Geometry, boundary conditions, and crack configuration. **b–f** Displacement contours for intersecting cracks with *β* = 15^°^, 30^°^, 45^°^, 60^°^, and 75^°^, respectively. For each angle, the *u*_*x*_ and *u*_*y*_ fields predicted by XDEM are compared with the FEM reference solution, and the corresponding error maps are computed with FEM taken as the reference solution. Source data are provided as a Source Data file.

Finally, to further illustrate the generality of XDEM, Fig. 3 presents results for intersecting cracks. XDEM accurately reproduces displacement discontinuities along multiple crack interfaces in close agreement with the reference solution^16^. These results highlight the robustness of XDEM in representing complex crack topologies without requiring adaptive meshing or problem-specific refinement.

### Crack propagation

The preceding section established that XDEM can accurately predict SIFs, forming the basis for modeling dynamic crack evolution. Here, we further validate XDEM’s performance in simulating crack propagation under mixed-mode loading, focusing on three representative scenarios: straight crack growth, crack kinking, and crack initiation. Detailed results for the additional examples are provided in Supplementary Note 2.2.

**Fig. 4:**
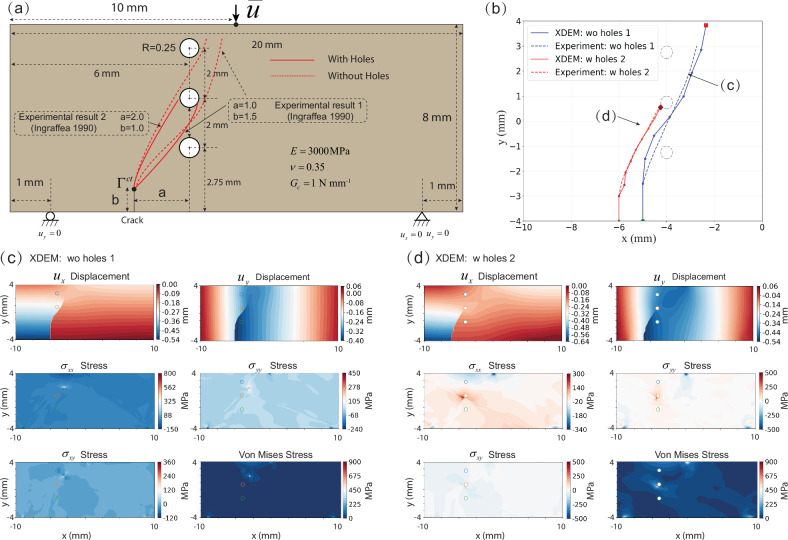
XDEM results for the Bittencourt problem. **a** Geometry of the Bittencourt setup. **b** Predicted crack propagation paths by XDEM compared against experimental observations^22^ for two scenarios: without a hole (Experiment 1) and with a hole (Experiment 2). **c** Displacement and stress contours for Experiment 1 (without hole). **d** Displacement and stress contours for Experiment 2 (with hole).

We first examine the classical Bittencourt problem^21^, a widely used benchmark in computational fracture mechanics. Experimental observations for this setup are available in ref. ^22^. As shown in Fig. 4a, this configuration is particularly interesting because the crack trajectory varies depending on the initial crack position, leading the crack to deflect toward different holes. The displacement field formulation used in XDEM for this problem is given in Supplementary Note 2.2.2.

In the XDEM simulations, crack propagation initiates at a normalized displacement $\bar{u}\approx 0.4$, consistent with previous experimental and numerical findings^23^. The predicted crack paths (Fig. 4b) show excellent agreement with the experimental observations^22^, accurately reproducing the deflection behavior observed in both scenarios-with and without a hole. The corresponding displacement and stress contours (Fig. 4c, d) clearly illustrate XDEM’s ability to capture displacement discontinuities and stress concentration near the crack tip during propagation.

**Fig. 5:**
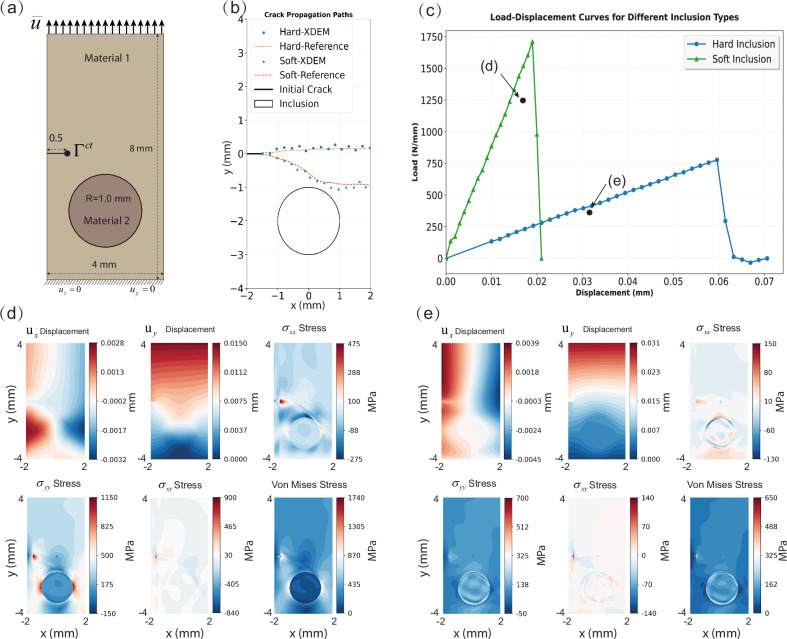
Performance of XDEM on crack inclusion problems. **a** schematic of the inclusion setup with two scenarios. Case 1: linear elasticity with soft inclusion, where the inclusion is a softer material with *E*_1_ = 210, 000N mm^−2^ and *E*_2_ = 21, 000N mm^−2^. Case 2: linear elasticity with hard inclusion, where the inclusion is stiffer, *E*_1_ = 21, 000N mm^−2^ and *E*_2_ = 210, 000N mm^−2^. The Poisson’s ratio and fracture energy are *ν* = 0.3 and *G*_c_ = 2.7N m^−1^ for all materials. **b** Comparison of crack propagation paths between XDEM and the reference solutions^24,25^. **c** Load-displacement curve predicted by XDEM. **d** Displacement and stress contour at $\bar{u}=0.015$ for the soft inclusion case. **e** Displacement and stress contour at $\bar{u}=0.0302$ for the hard inclusion case.

To further assess the robustness of XDEM, we next consider crack propagation in materials with stiffness inclusions, where the primary challenge arises from the interaction between materials of differing elastic properties. The benchmark setup follows^24,25^ and is illustrated in Fig. 5a. The displacement field used in XDEM is detailed in Supplementary Note 2.2.3. Figure 5b compares the predicted crack paths for soft and hard inclusions in linear elastic materials with corresponding reference solutions^24,25^. XDEM reproduces the reference trajectories with high fidelity for both cases, confirming its robustness across strong material heterogeneities. The load-displacement response predicted by XDEM (Fig. 5c) exhibits excellent agreement with established results, while the displacement and stress contours (Fig. 5d, e) demonstrate accurate resolution of both the stress concentration near the crack tip and the stress discontinuity across the inclusion interface.

## Discussion

In this work, we introduced the Extended Deep Energy Method (XDEM): a unified, physics-informed AI framework for fracture mechanics that seamlessly integrates discrete and continuous damage representations within a single variational formulation. By embedding explicit crack discontinuities and near-tip enrichments in the discrete setting, and coupling displacement and phase fields in the continuous setting, XDEM effectively overcomes several long-standing limitations of conventional DEM frameworks. In particular, XDEM eliminates the need for refined collocation near crack tips, maintains high accuracy with uniformly distributed sampling points, and enhances numerical stability across a wide spectrum of fracture scenarios. Comprehensive validation across canonical benchmarks-including stress intensity factor prediction, straight and kinked crack growth, crack initiation, inclusion-induced fracture, and three-dimensional crack propagation-demonstrates that XDEM achieves accuracy and efficiency on par with, and in many cases surpassing, traditional numerical approaches and standard DEM formulations. These results highlight XDEM’s ability to accurately capture both localized crack-tip fields and complex crack trajectories, thereby bridging the methodological gap between discrete and phase-field models. Beyond addressing key challenges in fracture simulation, XDEM establishes a scalable foundation for AI-driven computational mechanics. Its unified structure, variational grounding, and compatibility with transfer learning techniques position it as a powerful and data-efficient framework for large-scale simulations of complex materials and structures. Future extensions of XDEM to dynamic fracture, multiphysics coupling (e.g., thermo-mechanical or fluid-driven cracking), and neural operator-based generalization could further broaden its applicability and impact. In summary, XDEM provides a robust, accurate, and versatile framework that advances the state of the art in fracture mechanics while exemplifying how physics-informed AI can transform the predictive modeling of complex failure phenomena in engineering and materials science.

## Method

### Extended deep energy method framework

Extended Deep Energy Method (XDEM) unifies both discrete and continuous formulations of the Deep Energy Method (DEM) within a single framework, as illustrated schematically in Fig. 1. The discrete formulation captures sharp displacement discontinuities across cracks, while the continuous formulation models diffusive damage evolution through a phase-field representation. In the following, we introduce the discrete crack formulation and the continuous damage formulation, respectively.

### Discrete crack formulation

The loss functional of the discrete XDEM is defined as:

1 $${{{\bf{u}}}}^{n+1} 	=\arg {{\min }_{{{\bf{u}}}}}{{\Pi }},\\ {{\Pi }} 	={U}_{{{\rm{e}}}}-{W}_{{{\rm{ext}}}},\\ {U}_{{{\rm{e}}}} 	=\int_{\Omega }\frac{1}{2}\,{{\boldsymbol{\varepsilon }}}({{\bf{x}}};{{{\mathbf{\theta }}}}_{{{\rm{u}}}}):{{\bf{C}}}:{{\boldsymbol{\varepsilon }}}({{\bf{x}}};{{{\mathbf{\theta }}}}_{{{\rm{u}}}})\,dV,\\ {W}_{{{\rm{ext}}}} 	=\int_{\Omega }{{\bf{f}}}\cdot {{\bf{u}}}({{\bf{x}}};{{{\mathbf{\theta }}}}_{{{\rm{u}}}})\,dV+\int_{{\Gamma }^{{{\rm{t}}}}}\bar{{{\bf{t}}}}\cdot {{\bf{u}}}({{\bf{x}}};{{{\mathbf{\theta }}}}_{{{\rm{u}}}})\,dS,\\ \,{\mbox{s.t.}}\, {u}_{i}({{\bf{x}}};{{{\mathbf{\theta }}}}_{{{\rm{u}}}}) 	={\bar{u}}_{i}({{\bf{x}}},{t}^{n+1}),\,{{\bf{x}}}\in {\Gamma }^{{{\rm{u}}}};\quad {u}_{i}^{+}\not\equiv {u}_{i}^{-},\,{{\bf{x}}}\in {\Gamma }^{{{\rm{c}}}}.$$

Here, **σ,** **C,** ***ε***, and **u** represent the stress tensor, stiffness tensor, strain tensor, and displacement vector, respectively. *Ω* denotes the computational domain, while *Γ*^u^, *Γ*^t^, and *Γ*^c^ correspond to the Dirichlet, Neumann, and crack boundaries. The external load contributions **f,** $\bar{{{\bf{t}}}}$, and $\bar{{{\bf{u}}}}$ represent body forces, prescribed traction, and imposed displacements. Traction-free conditions are imposed along the crack surface *Γ*^c^, and mixed (Robin) boundaries are not considered here. In this formulation, the proposed crack function automatically enforces displacement discontinuity across *Γ*^c^, while the extended function enhances the representation of near-tip singular fields, significantly improving accuracy and convergence.

The key challenge for XDEM in discrete crack models is to accurately represent the displacement discontinuities across the crack surface and the highly localized fields near the crack tip. To this end, we incorporate the ideas of the Heaviside step function in XFEM^4^ and the asymptotic crack-tip solution into the Deep Energy Method. Specifically, the step function is used to capture displacement discontinuities, while the crack-tip enrichment function is employed to represent the singular behavior near the crack tip. In what follows, we describe how XDEM introduces these two components, i.e., crack and extended function.

#### Crack function

The treatment of displacement discontinuities in XDEM is achieved by introducing a crack function, which builds upon the concept of embedding functions proposed by Zhao et al.^16^.

In XDEM, the crack function *ϱ*(**x**) is required to satisfy:

2 $$\left\{\begin{array}{ll}{\lim }_{{{\bf{x}}}\to {{{\bf{x}}}}_{c}^{-}}\rho ({{\bf{x}}})\ne {{\lim }_{{{\bf{x}}}\to {{{\bf{x}}}}_{c}^{+}}}\rho ({{\bf{x}}}),\quad &{{{\bf{x}}}}_{c}\in {\Gamma }^{{{\rm{c}}}},\hfill\\ {\lim }_{{{\bf{x}}}\to {{{\bf{x}}}}_{0}}\rho ({{\bf{x}}})=\rho ({{{\bf{x}}}}_{0}),\hfill &{{{\bf{x}}}}_{0}\in \Omega \setminus {\Gamma }^{{{\rm{c}}}},\\ {\lim }_{{{\bf{x}}}\to {{{\bf{x}}}}_{0}}\nabla \rho ({{\bf{x}}})=\nabla \rho ({{{\bf{x}}}}_{0}),\hfill &{{{\bf{x}}}}_{0}\in \Omega \setminus {\Gamma }^{{{\rm{c}}}},\end{array}\right.$$

where *Γ*^c^ denotes the crack surface.

The crack function takes the following form:

3 $$\rho ({{\bf{x}}}) 	={f}_{1}({{\bf{x}}})\cdot {f}_{2}({{\bf{x}}})\\ {f}_{1}({{\bf{x}}}) 	={\mathrm{sgn}}\,\left({{{\rm{D}}}}_{s}({{\bf{x}}};{{\rm\Gamma }}^{{\mathrm{cl}}})\right)\\ {f}_{2}({{\bf{x}}}) 	={{\mathrm{ReLU}}}^{2}\,\left({{{\rm{D}}}}_{s}({{\bf{x}}};{{\rm\Gamma }}^{c1})\cdot {{{\rm{D}}}}_{s}({{\bf{x}}};{{\rm\Gamma }}^{c2})\right)\,\exp \left({{\rm{D}}}({{\bf{x}}};{{\rm\Gamma }}^{{\mathrm{cl}}})\right)$$

where ${{\rm{sgn}}}$, D_*s*_, and *D* are the sign function, the signed distance function, and the distance function, respectively, defined as:

4 $${\mathrm{sgn}}(x)=\left\{\begin{array}{cc}-1,& x\le 0\\ 1,& x > 0\end{array}\right.$$

5 $${{{\rm{D}}}}_{s}({{\bf{x}}};{\Gamma }^{{{\rm{cl}}}})={{\rm{sgn}}}\,\left({{\bf{n}}}\cdot ({{\bf{x}}}-\hat{{{\bf{x}}}})\right)\,\parallel {{\bf{x}}}-\hat{{{\bf{x}}}}\parallel,$$

6 $${{{\rm{D}}}}_{s}({{\bf{x}}};{{\rm\Gamma }}^{{\mathrm{cl}}})={\mathrm{sgn}}\,\left({{\bf{n}}}\cdot ({{\bf{x}}}-\hat{{{\bf{x}}}})\right)\,\parallel {{\bf{x}}}-\hat{{{\bf{x}}}}\parallel $$

with $\hat{{{\bf{x}}}}$ denoting the closest point on the crack extension line *Γ*^cl^ to **x**, and **n** being the outward normal vector at $\hat{{{\bf{x}}}}$. *Γ*^*c*1^ and *Γ*^*c*2^ represent the perpendicular extensions of the two endpoints of *Γ*^c^. Supplementary Fig. 23 illustrates the construction of the crack function *ϱ*(**x**) and its components.

The primary role of the crack function is to embed discontinuity information of cracks into the neural network. There are two possible strategies for incorporating the crack function: (1) include it as an additional input to the neural network, i.e., NN(x, *ϱ*; θ_u_), which is simple but increases the input dimension by one; or (2) incorporate it into the output of the neural network, which avoids increasing the input width but requires a more complex implementation. In this work, we focus on the first approach for its simplicity.

#### Extended function

To enable the neural network to better capture the near-tip singular fields, XDEM introduces the Williams series expansion^26^:

7 $$\left[\begin{array}{l}{u}_{{{\rm{x}}}}\\ {u}_{{{\rm{y}}}}\end{array}\right]=\mathop{\sum }_{n=0}^{+\infty }{A}_{n}^{{{\rm{I}}}}\,\frac{{r}^{\frac{n}{2}}}{2G}\left[\begin{array}{l}{f}_{{{\rm{x}}}n}^{{{\rm{I}}}}\\ {f}_{{{\rm{y}}}n}^{{{\rm{I}}}}\end{array}\right]+{\sum }_{n=0}^{+\infty }{A}_{n}^{{{\rm{II}}}}\,\left(-\frac{{r}^{\frac{n}{2}}}{2G}\right)\left[\begin{array}{l}{f}_{{{\rm{x}}}n}^{{{\rm{II}}}}\\ {f}_{{{\rm{y}}}n}^{{{\rm{II}}}}\end{array}\right],$$

where ${A}_{n}^{{{\rm{I}}}}$ and ${A}_{n}^{{{\rm{II}}}}$ are real constants corresponding to mode I and mode II cracks, *G* is the shear modulus, ${f}_{{{\rm{x}}}n}^{{{\rm{I}}}}$ and ${f}_{{{\rm{y}}}n}^{{{\rm{I}}}}$ are angular distribution functions for mode I, and ${f}_{{{\rm{x}}}n}^{{{\rm{II}}}}$ and ${f}_{{{\rm{y}}}n}^{mathrmII}$ are the corresponding functions for mode II:

8 $${f}_{{{\rm{x}}}n}^{{{\rm{I}}}} 	=\kappa \cos \left(\frac{n}{2}\theta \right)-\frac{n}{2}\cos \left[\left(\frac{n}{2}-2\right)\theta \right]+\left[\frac{n}{2}+{(-1)}^{n}\right]\cos \left(\frac{n}{2}\theta \right),\\ {f}_{{{\rm{y}}}n}^{{{\rm{I}}}} 	=\kappa \sin \left(\frac{n}{2}\theta \right)+\frac{n}{2}\sin \left[\left(\frac{n}{2}-2\right)\theta \right]-\left[\frac{n}{2}+{(-1)}^{n}\right]\sin \left(\frac{n}{2}\theta \right),\\ {f}_{{{\rm{x}}}n}^{{{\rm{II}}}} 	=\kappa \sin \left(\frac{n}{2}\theta \right)-\frac{n}{2}\sin \left[\left(\frac{n}{2}-2\right)\theta \right]+\left[\frac{n}{2}-{(-1)}^{n}\right]\sin \left(\frac{n}{2}\theta \right),\\ {f}_{{{\rm{y}}}n}^{{{\rm{II}}}} 	=-\kappa \cos \left(\frac{n}{2}\theta \right)-\frac{n}{2}\cos \left[\left(\frac{n}{2}-2\right)\theta \right]+\left[\frac{n}{2}-{(-1)}^{n}\right]\cos \left(\frac{n}{2}\theta \right),$$

where (*r*, *θ*) are local polar coordinates centered at the crack tip, with *θ* = 0 aligned with the crack tangent. The parameter *κ* depends on the problem type: for plane strain, *κ* = 3 − 4*ν*; for plane stress, *κ* = (3 − *ν*)/(1 + *ν*), with *ν* being Poisson’s ratio.

By combining the Williams series expansion in Eq. (7) with the embedding function, the displacement neural network is extended as follows:

9 $${u}_{1}({{\bf{x}}},\rho ;{{{\mathbf{\theta }}}}_{{{\rm{u}}}}) 	=D({{\bf{x}}};{\Gamma }_{{{\bf{u}}}})\,\left[{{{\rm{NN}}}}_{{{\rm{x}}}}({{\bf{x}}},\rho ;{{{\mathbf{\theta }}}}_{{{\rm{u}}}})+{\sum }_{i=1}^{{N}_{tip}}{{\rm{T}}}({{\bf{x}}};{\Gamma }^{{{\rm{c}}}(i)})\,{{{\rm{X}}}}_{1}({{\bf{x}}};{\Gamma }^{{{\rm{c}}}(i)})\right]+{\bar{u}}_{{{\rm{x}}}}({{\bf{x}}}),\\ {u}_{2}({{\bf{x}}},\rho ;{{{\mathbf{\theta }}}}_{{{\rm{u}}}}) 	=D({{\bf{x}}};{\Gamma }_{{{\bf{u}}}})\,\left[{{{\rm{NN}}}}_{{{\rm{y}}}}({{\bf{x}}},\rho ;{{{\mathbf{\theta }}}}_{{{\rm{u}}}})+{\sum }_{i=1}^{{N}_{tip}}{{\rm{T}}}({{\bf{x}}};{\Gamma }^{{{\rm{c}}}(i)})\,{{{\rm{X}}}}_{2}({{\bf{x}}};{\Gamma }^{{{\rm{c}}}(i)})\right]+{\bar{u}}_{y}({{\bf{x}}}),\\ {{{\rm{X}}}}_{1}({{\bf{x}}};{\Gamma }^{{{\rm{c}}}(i)}) 	={\sum }_{n=0}^{+\infty }{\alpha }_{n}^{{{\rm{I}}}}\,\frac{{r}^{\frac{n}{2}}}{2G}{f}_{{{\rm{x}}}n}^{{{\rm{I}}}}+{\sum }_{n=0}^{+\infty }{\alpha }_{n}^{{{\rm{II}}}}\,\left(-\frac{{r}^{\frac{n}{2}}}{2G}\right){f}_{{{\rm{x}}}n}^{{{\rm{II}}}},\\ {{{\rm{X}}}}_{2}({{\bf{x}}};{\Gamma }^{{{\rm{c}}}(i)}) 	={\sum }_{n=0}^{+\infty }{\beta }_{n}^{{{\rm{I}}}}\,\frac{{r}^{\frac{n}{2}}}{2G}{f}_{{{\rm{y}}}n}^{{{\rm{I}}}}+{\sum }_{n=0}^{+\infty }{\beta }_{n}^{{{\rm{II}}}}\,\left(-\frac{{r}^{\frac{n}{2}}}{2G}\right){f}_{{{\rm{y}}}n}^{{{\rm{II}}}},\\ {{\rm{T}}}({{\bf{x}}};{\Gamma }^{{{\rm{c}}}(i)}) 	=\exp (-m{r}^{q}),$$

where ${\alpha }_{n}^{{{\rm{I}}}}$, ${\alpha }_{n}^{{{\rm{II}}}}$, ${\beta }_{n}^{{{\rm{I}}}}$, and ${\beta }_{n}^{{{\rm{II}}}}$ are learnable parameters, typically initialized according to the crack type. For example, for mode I cracks, we recommend initializing ${\alpha }_{1}^{I}={\beta }_{1}^{I}={K}_{{{\rm{I}}}}/\sqrt{2\pi }=\sigma \sqrt{a}/\sqrt{2}$, where *a* is the crack length and *σ* the applied tensile stress. The remaining parameters can be initialized to zero. Since the asymptotic solution is more accurate closer to the crack tip, we introduce a decay function ${{\rm{T}}}({{\bf{x}}};{\Gamma }^{{{\rm{c}}}(i)})=\exp (-m{r}^{q})$ to reduce its influence away from the crack tip, where *m* and *q* are hyperparameters (typically *m* = *b*/*a*, *q* = 1), with *b* being a structural length scale and *a* the crack length. ${\bar{u}}_{{{\rm{x}}}}({{\bf{x}}})$ and ${\bar{u}}_{y}({{\bf{x}}})$ are prescribed displacement boundary conditions, while *u*_1_ and *u*_2_ represent the displacement components in the *x* and *y* directions, respectively. D(**x**; *Γ*_**u**_) denotes the distance from **x** to the Dirichlet boundary *Γ*_**u**_. The term T ⋅ X constitutes the *extended function* in XDEM. Supplementary Fig. 24 illustrates the extended function for an inclined crack.

Substituting Eq. (9) into Eq. (1), the optimization problem can be solved. It is important to note that the loading process must be divided into multiple increments, since fracture is path-dependent: even if the final load state is identical, different loading paths may lead to entirely different displacement fields and crack trajectories. After optimization, crack propagation follows a predefined fracture criterion. For instance, under the maximum circumferential stress criterion, the crack extends in the direction **n**_*c*_ of the maximum hoop stress, with an incremental length *α*_*c*_**n**_*c*_, where *α*_*c*_ is the prescribed step size.

Since the enriched displacement space better matches the true fracture fields, XDEM requires significantly fewer collocation points to achieve high accuracy. Unless otherwise stated, we employ uniformly distributed collocation points. We recommend using the discrete XDEM formulation when dealing with relatively simple crack problems.

Unlike conventional XFEM, which suffers from blending and topological enrichment issues, our global approximation framework in XDEM completely eliminates these difficulties.

### Continuous damage formulation

For the continuous (phase-field) setting, the loss functional of XDEM is expressed as:

10 $$	\{{{{\bf{u}}}}^{n+1},{\phi }^{n+1}\}=\arg {{\min }_{{{{\mathbf{\theta }}}}_{{{\rm{u}}}},{{{\mathbf{\theta }}}}_{{{\rm{\phi }}}}}}{{\Pi }}\left({{\bf{u}}}({{\bf{x}}};{{{\mathbf{\theta }}}}_{{{\rm{u}}}}),\phi ({{\bf{x}}};{{{\mathbf{\theta }}}}_{{{\rm{\phi }}}})\right),\\ 	 {{\Pi }}={U}_{{{\rm{e}}}}+{U}_{{{\rm{c}}}}-{W}_{{{\rm{ext}}}},\\ 	 {U}_{{{\rm{e}}}}({{\bf{u}}},\phi )=\int_{\Omega }\,\left[w\,\left(\phi ({{\bf{x}}};{{{\mathbf{\theta }}}}_{{{\rm{\phi }}}})\right)\,{\Psi }^{+}\,\left({{\bf{u}}}({{\bf{x}}};{{{\mathbf{\theta }}}}_{{{\rm{u}}}})\right)+{\Psi }^{-}\,\left({{\bf{u}}}({{\bf{x}}};{{{\mathbf{\theta }}}}_{{{\rm{u}}}})\right)\right]\,dV,\\ 	 {U}_{{{\rm{c}}}}({{\bf{u}}},\phi )=\frac{{G}_{{{\rm{c}}}}}{{c}_{{{\rm{w}}}}}\int_{\Omega }\,\frac{g\,\left(\phi ({{\bf{x}}};{{{\mathbf{\theta }}}}_{{{\rm{\phi }}}})\right)}{{l}_{0}}+{l}_{0}\,\nabla \phi ({{\bf{x}}};{{{\mathbf{\theta }}}}_{{{\rm{\phi }}}})\cdot \nabla \phi ({{\bf{x}}};{{{\mathbf{\theta }}}}_{{{\rm{\phi }}}})\,dV,\\ 	 {W}_{{{\rm{ext}}}}=\int_{\Omega }{{\bf{f}}}\cdot {{\bf{u}}}({{\bf{x}}};{{{\mathbf{\theta }}}}_{{{\rm{u}}}})\,dV+\int_{{\Gamma }^{{{\rm{t}}}}}\bar{{{\bf{t}}}}\cdot {{\bf{u}}}({{\bf{x}}};{{{\mathbf{\theta }}}}_{{{\rm{u}}}})\,dS,\\ \,{\mbox{s.t.}}\, 	 {u}_{i}({{\bf{x}}};{{{\mathbf{\theta }}}}_{{{\rm{u}}}})={\bar{u}}_{i}({{\bf{x}}},{t}^{n+1}),\,{{\bf{x}}}\in {\Gamma }^{{{\rm{u}}}};\quad {\phi }^{n+1}\ge {\phi }^{n}.$$

Here, **θ**_u_ and **θ**_ϕ_ denote the trainable parameters of the displacement and phase-field neural networks, respectively. Unlike the discrete formulation, the phase-field approach naturally captures crack nucleation and evolution without requiring an explicit crack-propagation criterion. The irreversibility constraint *ϕ*^*n*+1^≥*ϕ*^*n*^ is enforced to prevent crack healing, implemented via penalization or history-field mechanisms.

In the continuous damage formulation, XDEM introduces a phase-field variable to represent cracks. The displacement field and the phase field are approximated by separate neural networks. Since the phase field is more compatible with the functional space of radial basis functions (RBFs), we recommend approximating the phase field using an RBF network, with a detailed justification provided in Supplementary Note 3.5. On the other hand, as the displacement field exhibits strong variations near crack tips, and Kolmogorov–Arnold Networks (KANs) have been shown to perform better than MLPs for problems with sharp variations^27^, we recommend approximating the displacement field with a KAN. The architectures of the RBF and KAN networks are illustrated in Supplementary Fig. 25, and further details are given in Supplementary Note 3.6. It should be noted that there is no universally optimal architecture; the choice depends on the specific problem, analogous to the use of different element types in the finite element method.

For optimization, both monolithic and staggered strategies can be employed. The staggered scheme renders the energy functional landscape more convex, making it more robust than the monolithic scheme. However, the monolithic scheme often converges faster in practice. A detailed discussion is provided in Supplementary Note 3.7.

The irreversibility condition *ϕ*^*n*+1^≥*ϕ*^*n*^ for the phase field is typically enforced either by a history field approach^7^ or by penalization techniques^28^; see Supplementary Note 3.4 for details. We recommend employing the continuous phase-field formulation of XDEM when dealing with complex crack problems.

### Transfer learning in extended deep energy method

Regardless of whether XDEM is applied in its discrete or continuous formulation, crack propagation simulations require discretizing the loading process into multiple increments. At each load step, the network must be retrained, which leads to significant computational cost^15^. However, since the displacement and phase fields in neighboring load steps are strongly correlated and share similar patterns, transfer learning can be leveraged to accelerate phase-field fracture simulations.

In this work, we adopt the Low-Rank Adaptation (LoRA) method^29^ for efficient fine-tuning, which substantially reduces computational cost. LoRA provides a more general and flexible parameter-efficient transfer learning strategy compared with both full and lightweight fine-tuning^30^, as illustrated in Supplementary Fig. 26.

The idea of LoRA is to approximate weight updates through the product of two low-rank matrices **AB**, where ${{\bf{A}}}\in {{\mathbb{R}}}^{d\times r}$, ${{\bf{B}}}\in {{\mathbb{R}}}^{r\times m}$, and $r\ll \min (d,m)$. These low-rank updates are added to the pretrained weight matrix ${{\bf{W}}}\in {{\mathbb{R}}}^{d\times m}$, as shown in Supplementary Fig. 26:

11 $${{{\bf{W}}}}^{*}={{\bf{W}}}+\alpha {{\bf{A}}}{{\bf{B}}},$$

where *r* is the rank of LoRA, typically set to 1 in our XDEM implementation. It is important to note that **W** represents the fixed pretrained weights from the previous task and is not updated. Only **A** and **B** are trainable, and their number of parameters is *r*(*d* + *m*), which is significantly smaller than the *d* ⋅ *m* parameters required in full fine-tuning. The scalar *α* is a scaling factor that balances the contribution of pretrained weights **W** and the LoRA updates **AB**, and is set to 1 by default. **A** and **B** are initialized with Gaussian-distributed entries (mean 0, standard deviation 0.02).

LoRA can be interpreted as a generalized form of lightweight fine-tuning, while full fine-tuning can be seen as its limiting case when r=min(d,m). In essence, LoRA reduces the number of trainable parameters by decomposing the weight updates into low-rank factors.

In XDEM, we apply LoRA specifically to the displacement network, as LoRA generalizes both full and lightweight fine-tuning. Concretely, we adapt the parameters ${c}_{m}^{(i,j)}$ and *W*_*ij*_ in the KAN network according to Eq. (11), considering KAN network:

12 $${c}_{m}^{(ij)*} 	={c}_{m}^{(ij)}+{{{\bf{A}}}}_{{{\rm{c}}}}{{{\bf{B}}}}_{{{\rm{c}}}},\\ {W}_{ij}^{*} 	={W}_{ij}+{{{\bf{A}}}}_{{{\rm{w}}}}{{{\bf{B}}}}_{{{\rm{w}}}},$$

where $${{{\bf{A}}}}_{{{\rm{c}}}}\in {{\mathbb{R}}}^{{l}_{o}\times r}$$, $${{{\bf{B}}}}_{{{\rm{c}}}}\in {{\mathbb{R}}}^{r\times [{l}_{i}(G+r)]}$$, $${{{\bf{A}}}}_{{{\rm{w}}}}\in {{\mathbb{R}}}^{{l}_{o}\times r}$$, and $${{{\bf{B}}}}_{{{\rm{w}}}}\in {{\mathbb{R}}}^{r\times {l}_{i}}$$.

It should be noted that transfer can be performed incrementally between consecutive load steps. However, in the later stages of displacement-controlled loading, non-physical fracture patterns may appear (see Supplementary Note 3.1). This issue arises due to the delicate balance between the expressive power of neural networks and the accuracy of numerical integration. Although it is fundamentally unrelated to transfer learning algorithms like LoRA, this issue primarily stems from the numerical integration precision. In the future, however, a transfer learning algorithm that improves the integration accuracy in subsequent load steps could potentially be developed for solving fracture mechanics problems using PINNs. Therefore, in practice, XDEM often uses the parameters obtained from the first load step as the baseline for transfer learning. For the fracture propagation results in the manuscript, we use the parameters obtained from the first load step as the baseline for transfer learning.

In both discrete and continuous settings, the fracture process is simulated incrementally across loading steps. To enhance computational efficiency and reduce retraining costs, XDEM employs LoRA-based transfer learning between load increments-details.

### Statistics & reproducibility

No statistical method was used to predetermine sample size. The sample sizes in the numerical experiments, including the number of collocation points, loading steps, benchmark cases, and repeated simulations where applicable, were chosen based on standard practices in computational mechanics and convergence considerations. No data were excluded from the analyses. The experiments were not randomized. The Investigators were not blinded to allocation during experiments and outcome assessment. All numerical experiments were conducted using the computational framework described in the Methods. The geometries, material parameters, boundary conditions, loading protocols, network architectures, and training settings are reported in the manuscript and Supplementary Information to support reproducibility.

## Supplementary information

Supplementary Information

Transparent Peer Review file

## Source data

Source Data

## Data Availability

The source data underlying all plots shown in Figs. 2-5 and all figures in the Supplementary Information have been deposited in the Zenodo repository^31^ and are available at 10.5281/zenodo.20678658. Source data are provided with this paper.

## Code availability

The version of the XDEM code used in this study has been archived in Zenodo^32^ and is publicly available at 10.5281/zenodo.20690856. The active development repository is available at https://github.com/yizheng-wang/XDEM.
