# Supplementary material for: Towards unified AI-driven fracture mechanics: the extended deep energy method (XDEM)
Source: Nat Commun. 2026 Aug 15;17:8492. doi: 10.1038/s41467-026-76748-1 (PMC13484471; doi:10.1038/s41467-026-76748-1)
Supplement: Supplementary file 1 [file 41467_2026_76748_MOESM1_ESM.pdf]

Supplementary Information

Towards Unified AI-Driven Fracture Mechanics: The Extended Deep Energy Method (XDEM)

Nomenclature

Supplementary Table 1
Summary of the main symbols and notation used in this work.

Notation	Description
$\mathbf{u}$	Displacement field
$\boldsymbol{\varepsilon}$	Strain field
σ	Stress field
$\mathbf{C}$	Elastic stiffness tensor
$\mathbf{f}$	Body force
ϕ	Phase field variable
Γ^c	Crack surface
λ	First parameter of Lamé constants
E	Young's modulus
ν	Poisson's ratio
G	Shear modulus
K	Bulk modulus
G_c	Fracture energy release rate
l	Regularization (length scale) parameter
K_{I}, K_{II}, K_{III}	Mode I, II, and III stress intensity factors
J	J-integral
$\Psi^+(\boldsymbol{\varepsilon})$	Tensile part of the strain energy density
$\Psi^-(\boldsymbol{\varepsilon})$	Compressive part of the strain energy density
$w(\phi)$	Degradation function that represents the reduction of material stiffness
$g(\phi)$	Local dissipation function
$\mathbf{X}$	Learnable Williams series expansion in the extend function
$D(\mathbf{x})$	Distance function
T	Decay function
FEM	Finite Element Method
XDEM-D	Extended Deep Energy Method in discrete crack models
XDEM-C	Extended Deep Energy Method in continuous damage models

Supplementary Note 1 Prerequisite knowledge

This section introduces two fundamental frameworks in fracture mechanics, discrete and continuous crack models, under the assumptions of linear elasticity, small deformations, and quasi-static loading.

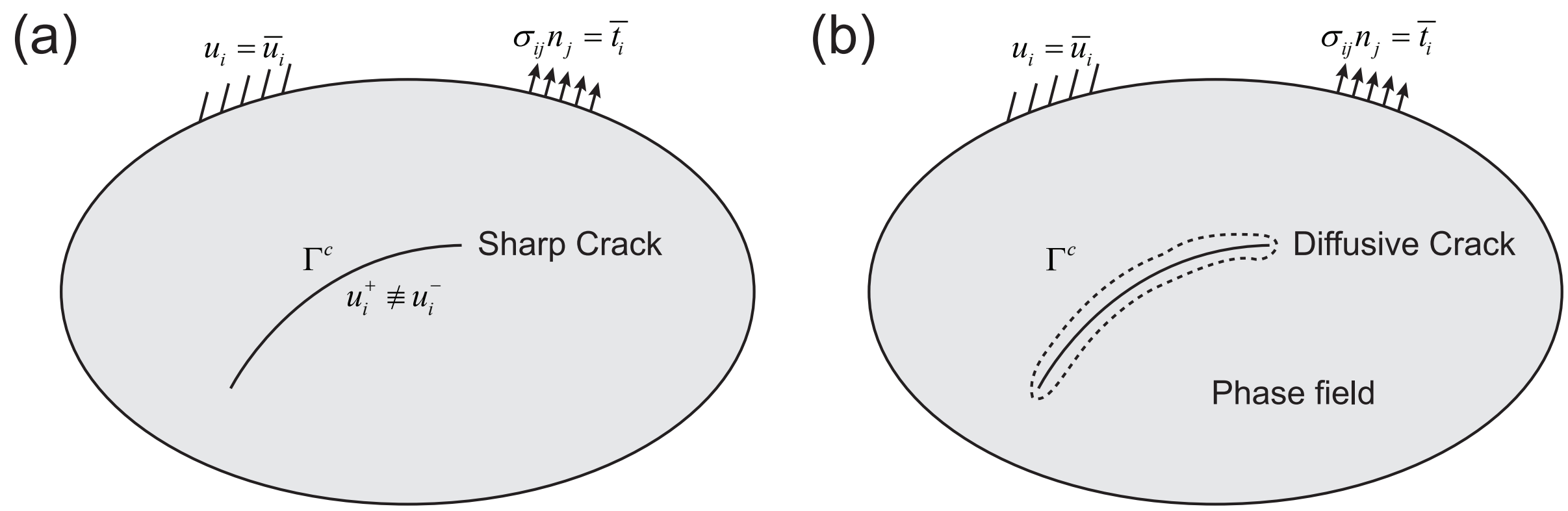

Supplementary Figure 1. Schematic of discrete and continuous damage models in fracture mechanics: (a) Discrete crack model with a sharp crack; (b) Continuous damage model where the sharp crack is regularized into a diffused zone.

1.1 Discrete crack model in fracture mechanics

The discrete crack model is essentially governed by the PDEs of linear elasticity:

$$
\begin{cases}
\sigma_{ij,j}(\boldsymbol{x}) + f_i(\boldsymbol{x}) = 0 & \mathbf{x} \in \Omega, \\
\sigma_{ij}(\boldsymbol{x}) = C_{ijkl}\varepsilon_{kl}(\boldsymbol{x}) & \mathbf{x} \in \Omega, \\
\varepsilon_{ij}(\boldsymbol{x}) = \frac{1}{2}(u_{i,j}(\boldsymbol{x}) + u_{j,i}(\boldsymbol{x})) & \mathbf{x} \in \Omega \setminus \Gamma^{\mathrm{c}}, \\
u_i^+(\boldsymbol{x}) \not\equiv u_i^-(\boldsymbol{x}) & \mathbf{x} \in \Gamma^{\mathrm{c}}, \\
\sigma_{ij}^{\pm} n_j^{\pm} = \bar{q}_i^{\pm} & \mathbf{x} \in \Gamma^{\mathrm{c}}, \\
\sigma_{ij}(\boldsymbol{x}) n_j = \bar{t}_i(\boldsymbol{x}) & \mathbf{x} \in \Gamma^{\mathrm{t}}, \\
u_i(\boldsymbol{x}) = \bar{u}_i(\boldsymbol{x}) & \mathbf{x} \in \Gamma^{\mathrm{u}}.
\end{cases}
\tag{1}
$$

Here, $\boldsymbol{\sigma}$, $\mathbf{C}$, $\boldsymbol{\varepsilon}$, and $\mathbf{u}$ denote the stress tensor, stiffness tensor, strain tensor, and displacement vector, respectively. The domain is Ω, with Γ^{u}, Γ^{t}, and Γ^{c} representing the Dirichlet boundary, Neumann boundary, and crack surface, respectively. $\mathbf{f}$, $\bar{\mathbf{t}}$, and $\bar{\mathbf{u}}$ denote the body force, prescribed traction, and prescribed displacement. $\bar{\mathbf{q}}$ denotes the external traction vector applied on the crack surface. The superscripts $\pm$ indicate the two opposing sides of the crack. The symbol $\not\equiv$ indicates a displacement discontinuity across Γ^{c}. In this work, only Dirichlet and Neumann boundary conditions are considered, with traction-free conditions $\bar{\mathbf{q}} = 0$ assumed on Γ^{c}. The boundaries satisfy $\Gamma^{\mathrm{t}} \cup \Gamma^{\mathrm{u}} = \Gamma$ and $\Gamma^{\mathrm{t}} \cap \Gamma^{\mathrm{u}} = \emptyset$. A schematic of the discrete crack model is shown in Supplementary Fig. 1a.

The system of equations Supplementary Eq. (1) can often be reformulated by the principle of minimum potential energy:

$$
\begin{aligned}
&\mathbf{u} = \arg\min_{\mathbf{u}} \Pi, \\
&\Pi = U_{\mathrm{e}} - W_{\mathrm{ext}}, \\
&U_{\mathrm{e}} = \int_{\Omega} \frac{1}{2}\boldsymbol{\varepsilon}(\boldsymbol{x}) : \mathbf{C} : \boldsymbol{\varepsilon}(\boldsymbol{x})\, dV, \\
&W_{\mathrm{ext}} = \int_{\Omega} \mathbf{f} \cdot \mathbf{u}(\boldsymbol{x})\, dV + \int_{\Gamma^{\mathrm{t}}} \bar{\mathbf{t}} \cdot \mathbf{u}\, dS + \int_{\Gamma^{\mathrm{c}}} \bar{\mathbf{q}}^{+} \cdot \mathbf{u}^{+} dS + \int_{\Gamma^{\mathrm{c}}} \bar{\mathbf{q}}^{-} \cdot \mathbf{u}^{-} dS, \\
\text{s.t. } & u_i(\boldsymbol{x}) = \bar{u}_i(\boldsymbol{x}),\ \mathbf{x} \in \Gamma^{\mathrm{u}}; \quad \forall\ u_i^+(\boldsymbol{x}) \not\equiv u_i^-(\boldsymbol{x}),\ \mathbf{x} \in \Gamma^{\mathrm{c}}.
\end{aligned}
\tag{2}
$$

It is straightforward to show that $\delta\Pi = 0$ is equivalent to Supplementary Eq. (1). By solving Supplementary Eq. (2), the displacement field can be obtained, from which the stress field follows via the constitutive and geometric relations. To further simulate crack propagation, a fracture criterion must be specified. Common criteria include the maximum circumferential stress criterion[1], the maximum energy release rate criterion[2], and the minimum strain energy density criterion[3]. In this work, we adopt the maximum circumferential stress criterion, whereby the crack propagates in the direction of the maximum hoop tensile stress.

1.2 Continuous damage model in fracture mechanics

Here we introduce the classical phase-field fracture model as a representative of continuous damage models. The phase-field method for fracture was first proposed by Bourdin et al. in 2000[4], based on the variational principle of Francfort and Marigo[5]. Unlike discrete models, the phase-field approach regularizes sharp cracks into diffused regions by introducing an additional damage-related phase-field variable, as illustrated in Supplementary Fig. 1b.

Starting from the energy functional in Supplementary Eq. (2), the fracture energy is incorporated as follows:

$$
\begin{aligned}
&\mathbf{u}, \phi = \arg\min_{\mathbf{u},\phi} \Pi, \\
&\Pi = U_{\mathrm{e}} + U_{\mathrm{c}} - W_{\mathrm{ext}}, \\
&U_{\mathrm{e}}(\mathbf{u},\phi) = \int_{\Omega} w(\phi)\,\Psi^{+}(\boldsymbol{\varepsilon}) + \Psi^{-}(\boldsymbol{\varepsilon})\, dV, \\
&U_{\mathrm{c}}(\mathbf{u},\phi) = \frac{G_{\mathrm{c}}}{c_w} \int_{\Omega} \frac{g(\phi)}{l_0} + l_0\, (\nabla\phi)\cdot(\nabla\phi)\, dV, \\
&W_{\mathrm{ext}} = \int_{\Omega} \mathbf{f}\cdot\mathbf{u}\, dV + \int_{\Gamma^{\mathrm{t}}} \bar{\mathbf{t}}\cdot\mathbf{u}\, dS, \\
&\text{s.t. } u_i = \bar{u}_i,\ \mathbf{x}\in\Gamma^{\mathrm{u}}; \quad \phi^{n+1} \geq \phi^{n}.
\end{aligned}
\tag{3}
$$

Here, $w(\phi)$ is the degradation function that represents the reduction of material stiffness. It must satisfy the following conditions: $w(0) = 1$, $w(1) = 0$, $w'(1) = 0$, and $w'(\phi) < 0$. A common choice is $w(\phi) = (1-\phi)^2$. $\Psi^{+}(\boldsymbol{\varepsilon})$ and $\Psi^{-}(\boldsymbol{\varepsilon})$ denote the tensile and compressive contributions of the strain energy, respectively. Typical decompositions include the formulations of Miehe[6] and Amor[7]:

$$
\begin{aligned}
\text{Miehe:}\quad & \Psi^{+}(\boldsymbol{\varepsilon}) = \tfrac{1}{2}\lambda\langle\varepsilon_{ii}\rangle_{+}^{2} + G\sum_{i=1}^{3}\langle\lambda_i\rangle_{+}^{2}, \\
& \Psi^{-}(\boldsymbol{\varepsilon}) = \tfrac{1}{2}\lambda\langle\varepsilon_{ii}\rangle_{-}^{2} + G\sum_{i=1}^{3}\langle\lambda_i\rangle_{-}^{2}, \\
\text{Amor:}\quad & \Psi^{+}(\boldsymbol{\varepsilon}) = \tfrac{1}{2}K\langle\varepsilon_{ii}\rangle_{+}^{2} + G\varepsilon'_{ij}\varepsilon'_{ij}, \\
& \Psi^{-}(\boldsymbol{\varepsilon}) = \tfrac{1}{2}K\langle\varepsilon_{ii}\rangle_{-}^{2},
\end{aligned}
\tag{4}
$$

where λ and G are the Lame constants, $K = \lambda + 2G/3$ is the bulk modulus, λ_i are the eigenvalues of the strain tensor $\boldsymbol{\varepsilon}$, and $\varepsilon'_{ij} = \varepsilon_{ij} - \varepsilon_{kk}\delta_{ij}/3$ is the deviatoric strain tensor. The positive and negative parts of a scalar are defined as $\langle x\rangle_{+} = (x+|x|)/2$ and $\langle x\rangle_{-} = (x-|x|)/2$. In the manuscript, we use Miehe as the form of the energy decomposition.

In Supplementary Eq. (3), G_{c} is the critical energy release rate, and $c_w = 4\int_0^1 \sqrt{g(\phi)}\, d\phi$ is a normalization constant. The function $g(\phi)$ denotes the local dissipation function, commonly chosen as in the AT1 ($g = \phi$, $c_w = 8/3$) or AT2 ($g = \phi^2$, $c_w = 2$) models.

In practical phase-field simulations, the choices of $w(\phi)$ and $g(\phi)$, the type of energy decomposition, and the length-scale parameter l_0 must be specified in advance. By minimizing Π in Supplementary Eq. (3), both the displacement field $\mathbf{u}$ and the phase-field variable ϕ can be obtained. Compared with discrete crack models, the phase-field model has the advantage of allowing spontaneous crack nucleation without prescribing a fracture criterion. However, its major drawback is the significantly higher computational cost. It is also important to note that the irreversibility condition $\phi^{n+1} \geq \phi^{n}$ is typically enforced by either a history field approach[6] or a penalization technique[8], as further discussed in Supplementary Note 3.4.

1.3 Deep Energy Method

The core idea of the Deep Energy Method (DEM) is to employ neural networks as trial functions to approximate the solution fields by directly minimizing the energy functional[9]. In fracture mechanics, the energy functional can be selected either from the discrete formulation[10] in Supplementary Eq. (2) or from the continuous phase-field formulation[11] in Supplementary Eq. (3).

For the discrete crack model, the DEM optimization process is formulated as:

$$\begin{aligned} \mathbf{u}^{n+1} &= \arg\min_{\mathbf{u}} \Pi \\ \Pi &= U_{\mathrm{e}} - W_{\mathrm{ext}} \\ U_{\mathrm{e}} &= \int_{\Omega} \frac{1}{2} \boldsymbol{\varepsilon}(\mathbf{x};\theta_{\mathrm{u}}) : \mathbf{C} : \boldsymbol{\varepsilon}(\mathbf{x};\theta_{\mathrm{u}}) dV \\ W_{\mathrm{ext}} &= \int_{\Omega} \mathbf{f} \cdot \mathbf{u}(\mathbf{x};\theta_{\mathrm{u}}) dV + \int_{\Gamma^{\mathrm{t}}} \bar{\mathbf{t}} \cdot \mathbf{u}(\mathbf{x};\theta_{\mathrm{u}}) dS. \\ \text{s.t. } & u_i(\mathbf{x};\theta_{\mathrm{u}}) = \bar{u}_i(\mathbf{x}, t^{n+1}), \mathbf{x} \in \Gamma^{\mathrm{u}}; u_i^+ \not\equiv u_i^-, \mathbf{x} \in \Gamma^{\mathrm{c}} \end{aligned} \tag{5}$$

Here, θ_{u} denotes the trainable parameters of the displacement neural network $\mathrm{NN}(\mathbf{x};\theta_{\mathrm{u}})$. The discontinuity of the displacement field across the crack surface, $u_i^+ \not\equiv u_i^-$, can be incorporated using subdomain DEM (CENN)[12] or discontinuity-embedded neural networks[10].

For the continuous phase-field fracture model, the DEM optimization problem is given by:

$$\begin{aligned} \{\mathbf{u}^{n+1}, \phi^{n+1}\} &= \arg\min_{\theta_{\mathrm{u}},\theta_{\phi}} \Pi(\mathbf{u}(\mathbf{x};\theta_{\mathrm{u}}), \phi(\mathbf{x};\theta_{\phi})) \\ \Pi &= U_{\mathrm{e}} + U_{\mathrm{c}} - W_{\mathrm{ext}} \\ U_{\mathrm{e}}(\mathbf{u},\phi) &= \int_{\Omega} [w(\phi(\mathbf{x};\theta_{\phi}))\Psi^{+}(\mathbf{u}(\mathbf{x};\theta_{\mathrm{u}})) + \Psi^{-}(\mathbf{u}(\mathbf{x};\theta_{\mathrm{u}}))] dV \\ U_{\mathrm{c}}(\mathbf{u},\phi) &= \frac{G_{\mathrm{c}}}{c_w} \int_{\Omega} \frac{g(\phi(\mathbf{x};\theta_{\phi}))}{l_0} + l_0 (\nabla\phi(\mathbf{x};\theta_{\phi})) \cdot (\nabla\phi(\mathbf{x};\theta_{\phi})) dV, \\ W_{\mathrm{ext}} &= \int_{\Omega} \mathbf{f} \cdot \mathbf{u}(\mathbf{x};\theta_{\mathrm{u}}) dV + \int_{\Gamma^{\mathrm{t}}} \bar{\mathbf{t}} \cdot \mathbf{u}(\mathbf{x};\theta_{\mathrm{u}}) dS. \\ \text{s.t. } & u_i(\mathbf{x};\theta_{\mathrm{u}}) = \bar{u}_i(\mathbf{x}, t^{n+1}), \mathbf{x} \in \Gamma^{\mathrm{u}}; \phi^{n+1} \geq \phi^{n} \end{aligned} \tag{6}$$

where θ_{u} and θ_{ϕ} denote the trainable parameters of the displacement neural network $\mathrm{NN}(\mathbf{x};\theta_{\mathrm{u}})$ and the phase-field neural network $\mathrm{NN}(\mathbf{x};\theta_{\phi})$, respectively. Unlike discrete models, the phase-field formulation does not require a predefined crack propagation criterion, as cracks can nucleate and evolve naturally. However, the irreversibility condition $\phi^{n+1} \geq \phi^{n}$ must be satisfied, ensuring that cracks cannot heal once formed.

Supplementary Note 2 Additional numerical examples

2.1 Stress intensity factor

2.1.1 Mode I crack

We first consider a standard mode I crack problem. The geometry consists of a plate with crack length $2a = 1$, centered at the middle of the structure. The overall dimensions are $2b = 2$ in length and $2h = 6$ in height. The material properties are $E = 1000\,\mathrm{MPa}$ and Poisson's ratio $\nu = 0.3$ under plane strain conditions. A uniform tensile stress $\sigma_0 = 100\,\mathrm{MPa}$ is applied on the top boundary, while the bottom boundary is constrained only in the y-direction ($u_{\mathrm{y}} = 0$). The left and right boundaries are traction-free. To suppress rigid body motions, we additionally fix $u_{\mathrm{x}} = 0$ at the crack center, which coincides with the origin of the coordinate system.

It is worth noting that, theoretically, only half of the structure (either left or right) needs to be modeled due to symmetry in the x-direction. However, in order to better illustrate the role of the embedding function and to make the problem more accessible for non-mechanics readers, we simulate the entire domain, as shown in Supplementary Fig. 2. The displacement fields in XDEM are expressed as:

$$\begin{aligned} u_1(\mathbf{x},\rho;\theta_{\mathrm{u}}) &= \frac{x}{b}\left[\mathrm{NN}_{\mathrm{x}}(\mathbf{x},\rho;\theta_{\mathrm{u}}) + \sum_{i=2} \mathrm{T}(\mathbf{x};\Gamma^{\mathrm{c}(i)}) \cdot \mathrm{X}_1(\mathbf{x};\Gamma^{\mathrm{c}(i)})\right], \\ u_2(\mathbf{x},\rho;\theta_{\mathrm{u}}) &= \frac{y+h}{2h}\left[\mathrm{NN}_{\mathrm{y}}(\mathbf{x},\rho;\theta_{\mathrm{u}}) + \sum_{i=2} \mathrm{T}(\mathbf{x};\Gamma^{\mathrm{c}(i)}) \cdot \mathrm{X}_2(\mathbf{x};\Gamma^{\mathrm{c}(i)})\right], \end{aligned} \tag{7}$$

Supplementary Fig. 3 shows the displacement and stress contours predicted by XDEM for different crack lengths a. The discontinuity in u_{y} and the stress concentration near the crack tips can be clearly observed. The training setup employs a multilayer perceptron (MLP) with 4 hidden layers and 30 neurons per layer, using the tanh activation function. The network input consists of the spatial coordinates (x,y) together with the embedded crack function, and the outputs are the displacement

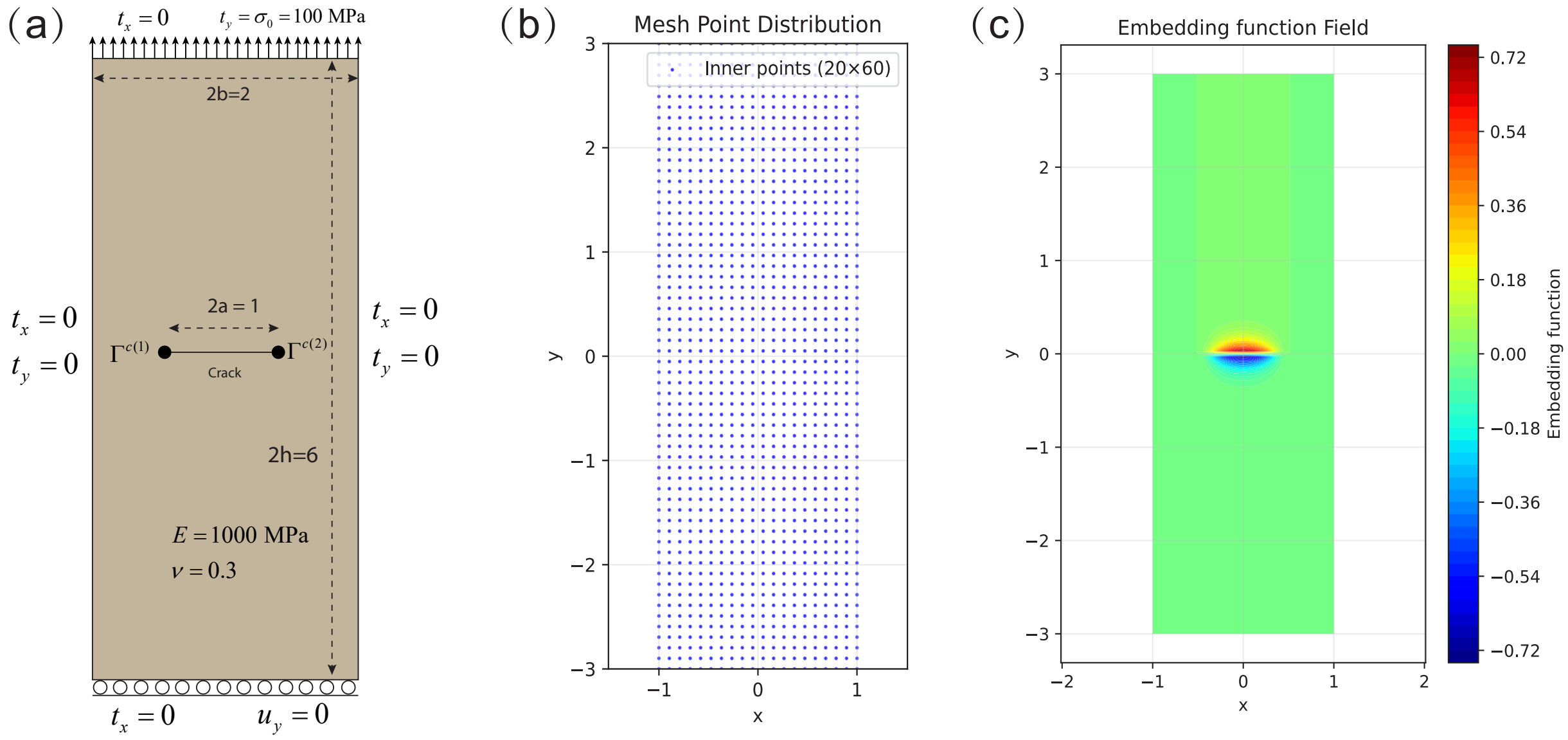

Supplementary Figure 2. Schematic of the mode I crack problem: (a) geometry, material properties, and boundary conditions; (b) collocation points for XDEM, uniformly distributed with 20×60 points; (c) contour of the embedding function.

components (u, v). The optimizer is Adam with an initial learning rate of 0.001. Training is performed for 15,000 epochs with early stopping (patience = 10) to prevent overfitting. A step-wise learning rate scheduler reduces the learning rate by a factor of 0.5 every 5,000 epochs.

For validation, we compare the predicted stress intensity factors against the reference solution from[13]:

$$K_\mathrm{I} = \sigma_0 \sqrt{\pi a} \left[1 - 0.025 \left(\frac{a}{b} \right)^2 + 0.06 \left(\frac{a}{b} \right)^4 \right] \sqrt{\sec \left(\frac{\pi a}{2b} \right)}. \tag{8}$$

Supplementary Fig. 4a compares XDEM with the standard DEM. It is evident that, under the same collocation scheme, XDEM captures the SIF more accurately than DEM. Importantly, XDEM and DEM exhibit comparable efficiency since the extended function in XDEM is analytical and only requires learning a few additional coefficients. Supplementary Fig. 4b shows the predictions of XDEM for different crack lengths, which agree very well with the analytical solution in Supplementary Eq. (8), confirming the accuracy of the displacement and stress fields in Supplementary Fig. 3. The SIFs are computed using the interaction integral method, as described in Supplementary Note 3.8, with the J-integral taken along a circular contour centered at the crack tip with radius $\min\left(\frac{a}{2}, \frac{b-a}{2}\right)$:

$$K_\mathrm{I} = \sqrt{J \frac{E}{1 - \nu^2}}. \tag{9}$$

For comparison, FEM simulations were also performed. With 46,014 CPE8 elements and 138,878 nodes, the FEM-predicted SIF was 148.7, requiring 35 seconds of computation. By contrast, XDEM required only $20 \times 60 = 1200$ collocation points and 1000 training epochs, taking 14 seconds to achieve comparable accuracy. Thus, XDEM, similar in spirit to XFEM, effectively captures displacement discontinuities through the crack function and enhances the representation of the crack-tip fields with the extended function.

2.1.2 Mode II crack

We next consider the standard mode II crack problem. The geometry consists of a plate with crack length $2a = 1$, centered at the middle of the structure. The overall dimensions are $2b = 2$ in length and $2h = 6$ in height. The material properties are $E = 1000\,\mathrm{MPa}$ and Poisson's ratio $\nu = 0.3$ under plane strain conditions. A uniform shear stress $\tau_0 = 100\,\mathrm{MPa}$ is applied on the top boundary. All boundaries are constrained in the y-direction ($u_\mathrm{y} = 0$), and the bottom boundary is further constrained in the x-direction ($u_\mathrm{x} = 0$). The schematic of the mode II crack problem is shown in Supplementary Fig. 5a. The displacement

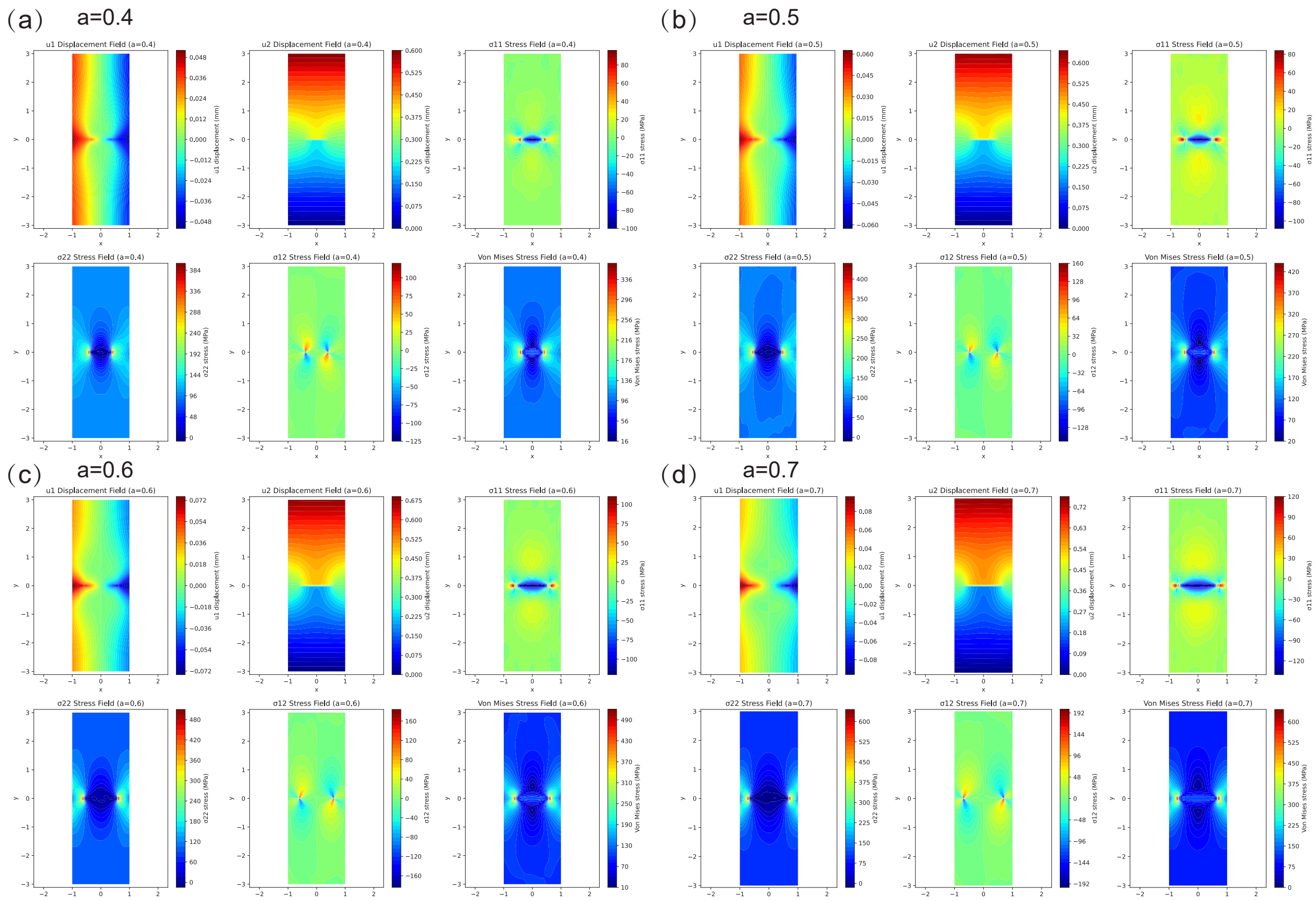

Supplementary Figure 3. XDEM-predicted displacement and stress contours for different crack lengths in the mode I crack problem.

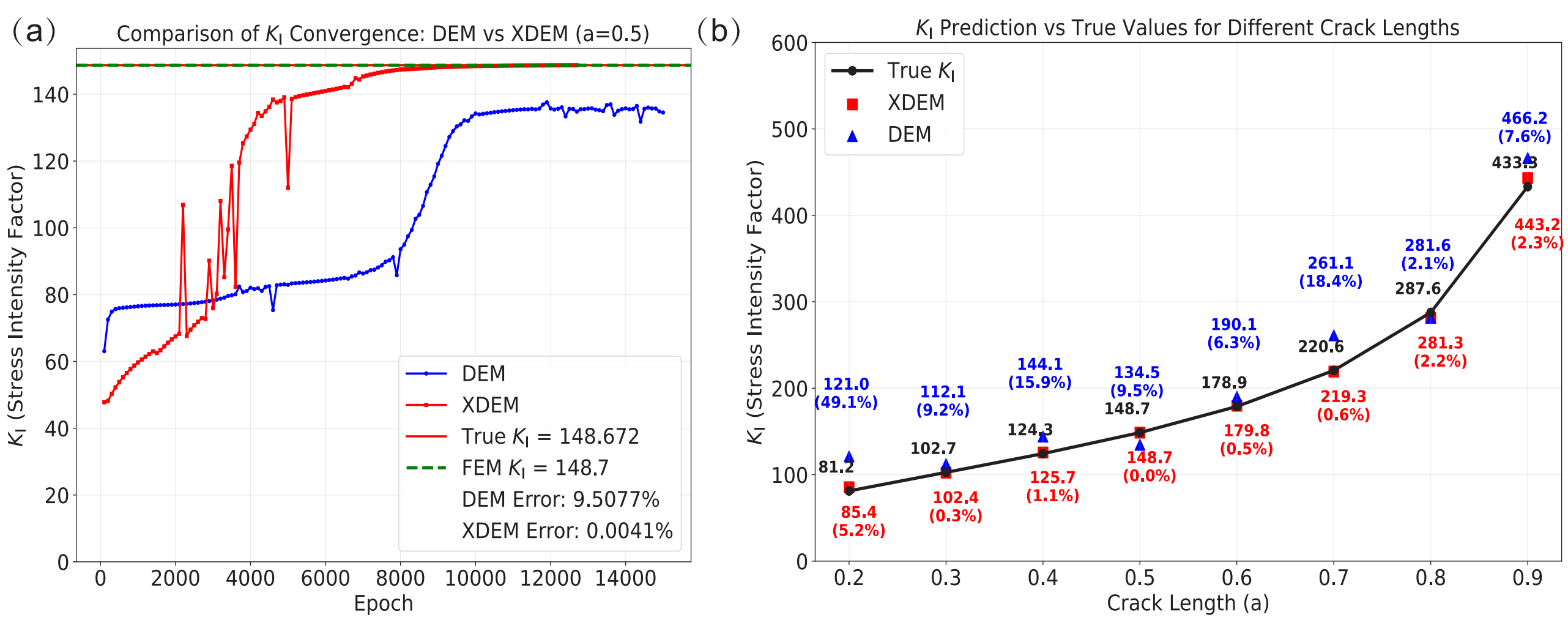

Supplementary Figure 4. Comparison of stress intensity factors in the mode I crack problem: (a) convergence of SIFs with uniformly distributed 20×60 points for XDEM and DEM without enrichment, with the reference value $K_{\mathrm{I}} = 148.672$; (b) XDEM and DEM predicted SIFs for different crack lengths with uniformly distributed 100×600 points. The relative errors are in brackets.

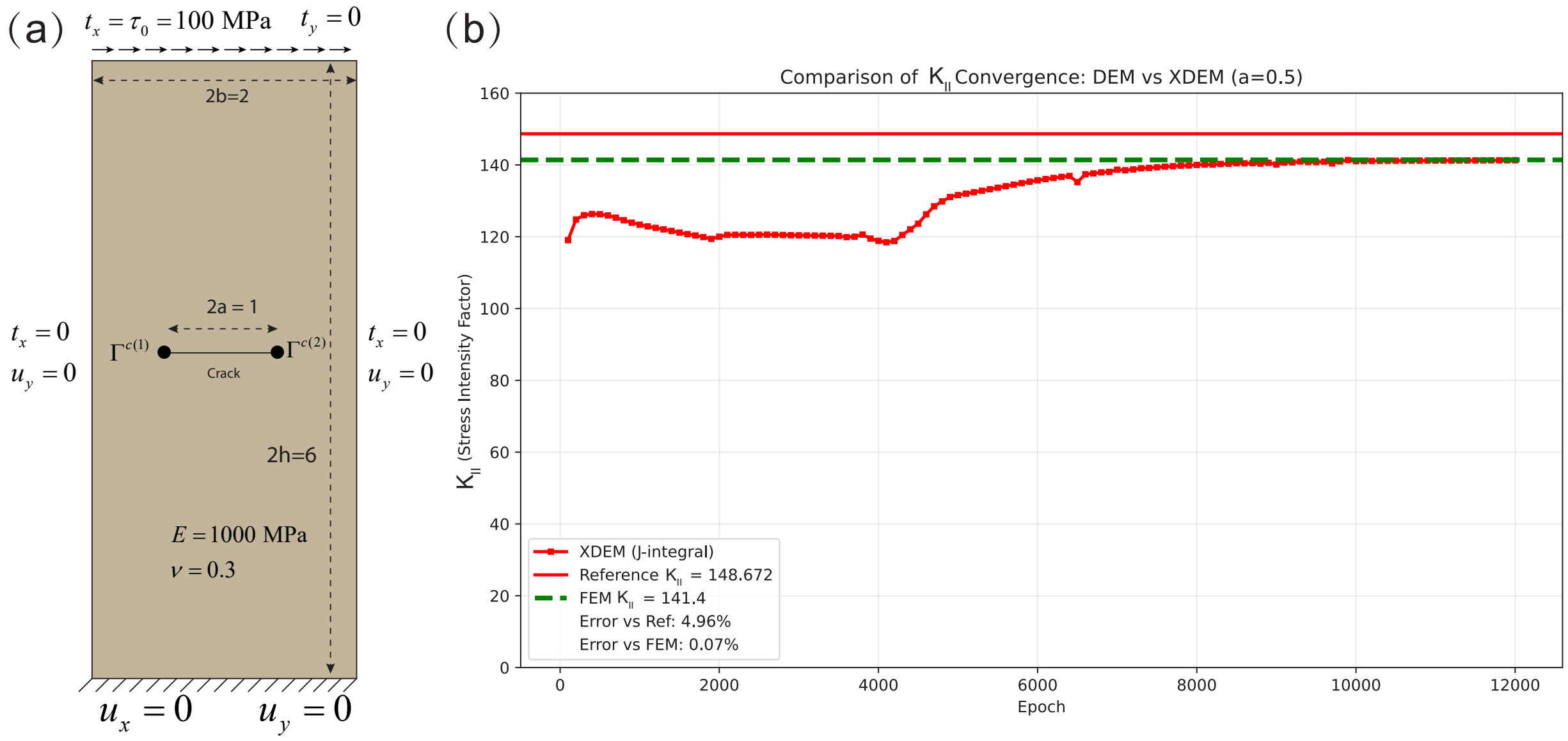

Supplementary Figure 5. Mode II crack problem: (a) geometry, material properties, and boundary conditions; (b) comparison of stress intensity factors predicted by XDEM, FEM, and the reference solution for $a = 0.5$ and $\tau_0 = 100\,\text{MPa}$, where the SIF is computed using the J-integral method.

fields in XDEM are expressed as:

$$
\begin{aligned}
u_1(\mathbf{x},\rho;\theta_{\text{u}}) &= \frac{y+h}{2h}\left[\text{NN}_{\text{x}}(\mathbf{x},\rho;\theta_{\text{u}}) + \sum_{i=2} \text{T}(\mathbf{x};\Gamma^{\text{c}(i)})\cdot \text{X}_1(\mathbf{x};\Gamma^{\text{c}(i)})\right],\\
u_2(\mathbf{x},\rho;\theta_{\text{u}}) &= \frac{h+y}{2h}\cdot\frac{h-y}{2h}\cdot\frac{b+x}{2b}\cdot\frac{b-x}{2b}\left[\text{NN}_{\text{y}}(\mathbf{x},\rho;\theta_{\text{u}}) + \sum_{i=2} \text{T}(\mathbf{x};\Gamma^{\text{c}(i)})\cdot \text{X}_2(\mathbf{x};\Gamma^{\text{c}(i)})\right].
\end{aligned}
\tag{10}
$$

The training setup is consistent with that of the mode I crack problem. In this example, we use 100×600 uniformly distributed collocation points. Supplementary Fig. 5b demonstrates that XDEM provides accurate predictions of the stress intensity factor, highlighting its effectiveness. The reference SIF is given by:

$$
K_{\text{II}} = \tau_0\sqrt{\pi a}\left[1 - 0.025\left(\frac{a}{b}\right)^2 + 0.06\left(\frac{a}{b}\right)^4\right]\sqrt{\sec\left(\frac{\pi a}{2b}\right)}. \tag{11}
$$

It is worth noting that, due to the boundary conditions not being purely shear loading, the analytical expression in Supplementary Eq. (11) yields a small deviation. Therefore, the SIF predicted by XDEM is evaluated using the J-integral:

$$
K_{\text{II}} = \sqrt{J\frac{E}{1-\nu^2}}. \tag{12}
$$

For reference, we also compute the SIF using FEM, which serves as a reliable benchmark after convergence analysis. The FEM model consists of 174,633 CPE8 elements and 525,787 nodes, with a total runtime of 61 seconds. Supplementary Fig. 6a shows the XDEM predictions of K_{II} under different loading conditions, while Supplementary Fig. 7 presents the corresponding displacement and stress contours, clearly exhibiting the discontinuity in u_{y} and stress concentration near the crack tips. Supplementary Fig. 6b further shows the effect of network architecture: as long as the neural network exceeds a certain threshold in complexity, XDEM is able to accurately predict the SIF for mode II cracks. These results confirm that XDEM delivers robust and accurate performance for mode II fracture problems across different loads and network configurations.

2.1.3 Mode III crack

Next, we analyze the performance of XDEM for a Mode III crack. Mode III is an anti-plane shear problem in which the displacement field is out of the plane. The governing PDE simplifies to the Poisson equation: $\nabla^2 u(\mathbf{x}) = 0$.

In the previous problems, the results were verified using reference solutions such as FEM rather than analytical solutions. Therefore, here we employ the analytical solution:

$$
w = u_3 = \frac{K_{\text{III}}}{G}\sqrt{\frac{2r}{\pi}}\sin\left(\frac{\theta}{2}\right), \tag{13}
$$

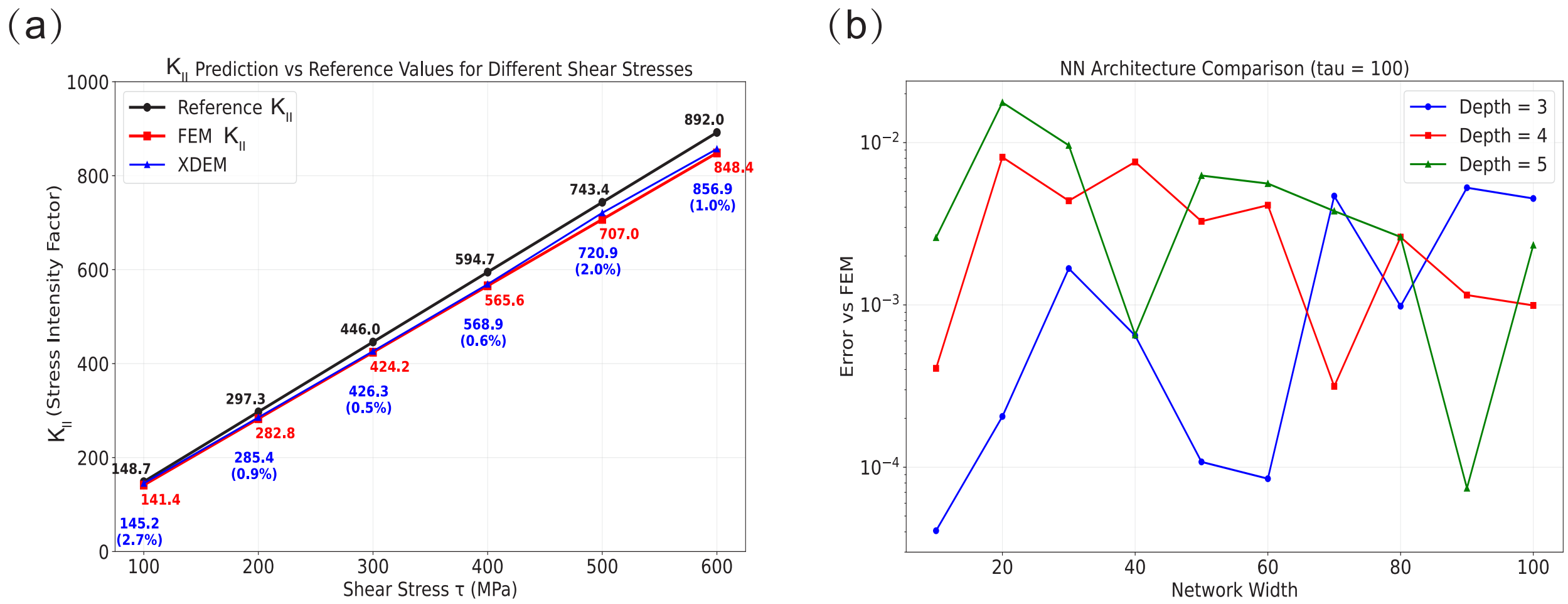

Supplementary Figure 6. Performance of XDEM for the mode II crack problem: (a) predicted SIFs for different load levels with $a = 0.5$ (trained for 10,000 steps); (b) effect of neural network architecture, with errors evaluated against the FEM reference solution.

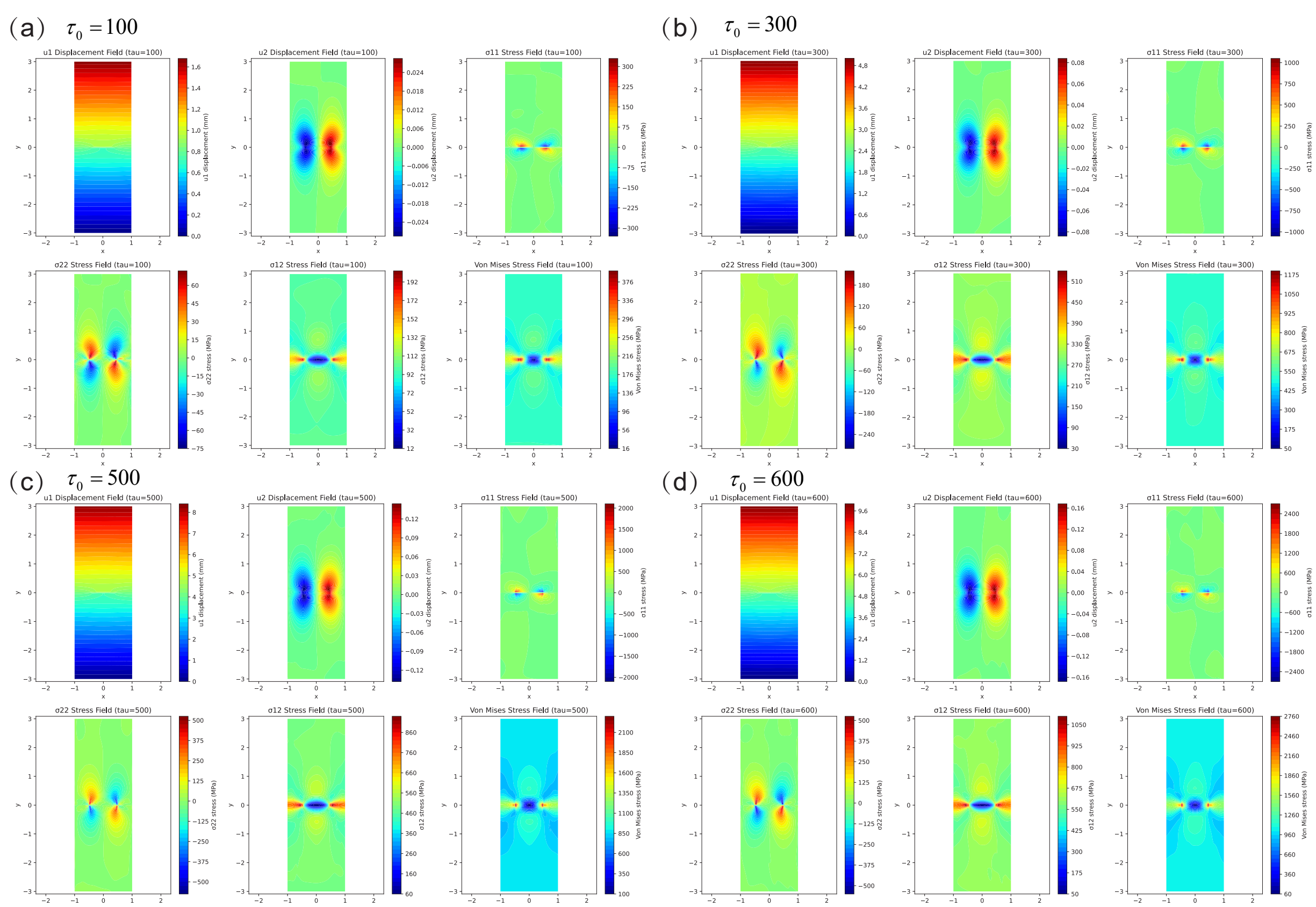

Supplementary Figure 7. Displacement and stress contours predicted by XDEM under different load levels for the mode II crack problem.

where $K_{\mathrm{III}} = 1$ MPa$\sqrt{\mathrm{mm}}$ is the Mode III stress intensity factor, and $G = 1$ MPa is the shear modulus. The displacement boundary conditions defined by Supplementary Eq. (13) are imposed on $x = \pm 1$ and $y = \pm 1$, while traction-free conditions are applied along the crack faces. The admissible displacement field in XDEM is expressed as

$$w(\mathbf{x}, \rho; \theta_{\mathrm{u}}) = u_p(\mathbf{x}) + \left(\frac{1+y}{2}\right)\left(\frac{1-y}{2}\right)\left(\frac{1+x}{2}\right)\left(\frac{1-x}{2}\right)\left[\mathrm{NN}(\mathbf{x}, \rho; \theta_{\mathrm{u}}) + \mathrm{T}(\mathbf{x}; \Gamma^{\mathrm{ct}}) X_3(\mathbf{x}; \Gamma^{\mathrm{ct}})\right], \tag{14}$$

where X_3 denotes the extended function, and $u_p(\mathbf{x})$ is the particular solution satisfying $u_p(\mathbf{x}) = u_3$ on the essential boundaries. The total loss function of XDEM is formulated as

$$\mathscr{L}_{\mathrm{XDEM}} = \int_{\Omega} \frac{1}{2} (\nabla w) \cdot (\nabla w)\, d\Omega. \tag{15}$$

The neural network architecture and training setup are identical to those used in the Mode I and II crack problems.

Supplementary Fig. 8(b) shows the stress intensity factors and singular strain predictions obtained by XDEM at different radii. The analytical expression of the singular strain is given by

$$\varepsilon_{z\theta}\big|_{\mathrm{interface}} = \frac{K_{\mathrm{III}}}{G}\sqrt{\frac{2}{\pi}}\frac{1}{r}\frac{\partial w}{\partial \theta} = \frac{1}{2\sqrt{r}}\cos\left(\frac{\theta}{2}\right)\bigg|_{\theta=0} = \frac{K_{\mathrm{III}}}{G}\frac{1}{\sqrt{2\pi r}}. \tag{16}$$

It can be observed that XDEM accurately captures the singular strain near the crack tip. Furthermore, we evaluate the accuracy of the stress intensity factors computed from the J-integral at different contour radius. Supplementary Fig. 8(c) presents the evolution of $\mathscr{L}_2$ and $\mathscr{H}_1$ errors during training, where $\mathscr{L}_2$ measures the L^2 norm of displacement errors, and $\mathscr{H}_1$ represents the H^1 norm of the strain energy density error. Rapid convergence of both metrics confirms the efficiency of XDEM. Supplementary Fig. 8(d-f) illustrate the analytical solution, XDEM prediction, and absolute error contour, respectively.

2.1.4 Mixed-mode crack

The displacement fields in XDEM are expressed as:

$$\begin{aligned} u_1(\mathbf{x}, \rho; \theta_{\mathrm{u}}) &= \frac{b+x}{2b} \cdot \frac{b-x}{2b}\left[\mathrm{NN_x}(\mathbf{x}, \rho; \theta_{\mathrm{u}}) + \sum_{i=2} \mathrm{T}(\mathbf{x}; \Gamma^{\mathrm{c}(i)}) \cdot \mathrm{X}_1(\mathbf{x}; \Gamma^{\mathrm{c}(i)})\right], \\ u_2(\mathbf{x}, \rho; \theta_{\mathrm{u}}) &= \frac{h+y}{2h}\left[\mathrm{NN_y}(\mathbf{x}, \rho; \theta_{\mathrm{u}}) + \sum_{i=2} \mathrm{T}(\mathbf{x}; \Gamma^{\mathrm{c}(i)}) \cdot \mathrm{X}_2(\mathbf{x}; \Gamma^{\mathrm{c}(i)})\right]. \end{aligned} \tag{17}$$

2.2 Crack propagation

In Supplementary Note 2.1, we demonstrated that XDEM can accurately predict stress intensity factors (SIFs), laying the groundwork for its application to crack growth problems. In this section, we further validate XDEM for simulating crack propagation, covering three classical scenarios: straight crack growth, kinking, inclusion, and crack initiation.

2.2.1 Straight crack propagation

The single-edge notched tension (SENT) specimen is a classical benchmark in fracture mechanics and is widely used for method validation. Supplementary Fig. 9a shows the geometry and material settings for the mode-I crack, where the crack propagates in a straight path. Both the discrete and continuous variants of XDEM are capable of simulating crack growth for this problem. The displacement field used in XDEM is:

$$\begin{aligned} u_1(\mathbf{x}, \rho; \theta_{\mathrm{u}}) &= \frac{h+y}{2h}\left[\mathrm{NN_x}(\mathbf{x}, \rho; \theta_{\mathrm{u}}) + \mathrm{T}(\mathbf{x}; \Gamma^{\mathrm{ct}})\, \mathrm{X}_1(\mathbf{x}; \Gamma^{\mathrm{ct}})\right], \\ u_2(\mathbf{x}, \rho; \theta_{\mathrm{u}}) &= \frac{h+y}{2h} \cdot \frac{h-y}{2h}\left[\mathrm{NN_y}(\mathbf{x}, \rho; \theta_{\mathrm{u}}) + \mathrm{T}(\mathbf{x}; \Gamma^{\mathrm{ct}})\, \mathrm{X}_2(\mathbf{x}; \Gamma^{\mathrm{ct}})\right] + \frac{h+y}{2h}\bar{u}, \end{aligned} \tag{18}$$

where Γ^{ct} denotes the crack tip and $\bar{u}$ is the prescribed displacement load.

For the discrete model (XDEM-D), the loading is divided into increments. At each increment, we compute the J-integral at the current crack tip. If $J > G_{\mathrm{c}}$, we evaluate K_{I} and K_{II} and determine the direction θ_c of maximum circumferential (hoop) stress in the local polar coordinate system (centered at the crack tip with $\theta = 0$ aligned with the crack tangent); see Supplementary Note 3.8 for details. The crack is then advanced by a small prescribed length $\delta a = 0.05$ along θ_c. After extension by δa, K_{I} and K_{II} are recomputed and the condition $J \lesseqgtr G_{\mathrm{c}}$ is rechecked, repeating the process until $J \leq G_{\mathrm{c}}$, at which point the algorithm proceeds to the next load step. The network architecture and training settings follow those in Supplementary Note 2.1.1, with 5000 training iterations per load increment.

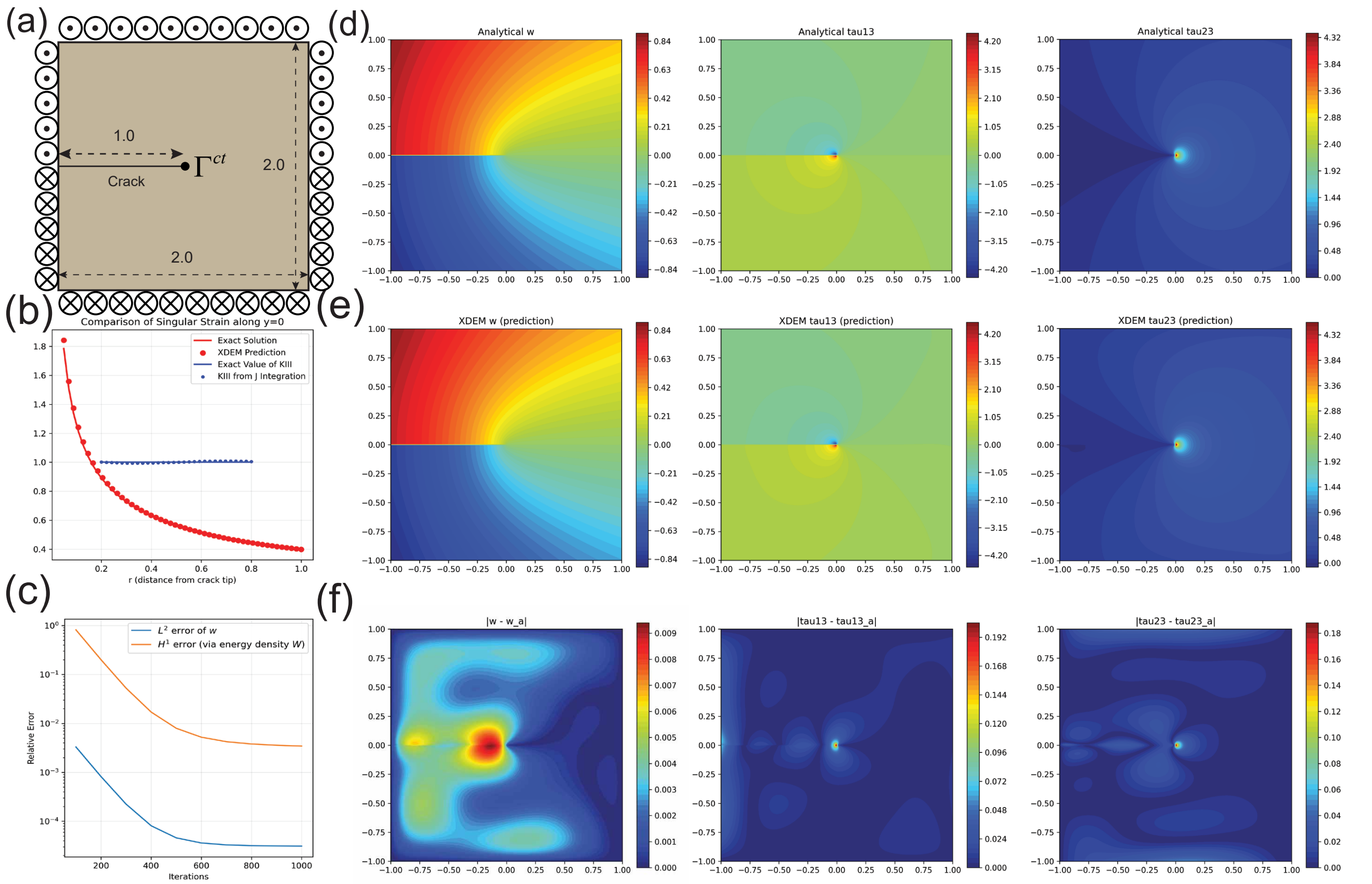

Supplementary Figure 8. Performance of XDEM for the Mode III crack: (a) schematic of the mode III. This is an anti-plane shear problem where the displacement is perpendicular to the x-y plane. Symbols with a cross ($\otimes$) indicate displacements directed into the plane, while dots ($\odot$) indicate displacements directed out of the plane. $K_{\text{III}} = 1$ MPa$\sqrt{\text{mm}}$, $G = 1$ MPa, specimen dimensions of 2 mm $\times$ 2 mm, and crack length of 1 mm; (b) comparison between the analytical and XDEM-predicted singular strain ($x > 0$, $y = 0$) and the stress intensity factors computed from the J-integral at various contour radii; (c) evolution of $\mathscr{L}_2$ and $\mathscr{H}_1$ errors during iterations; (d-f) analytical solution, XDEM prediction, and absolute error contour, respectively.

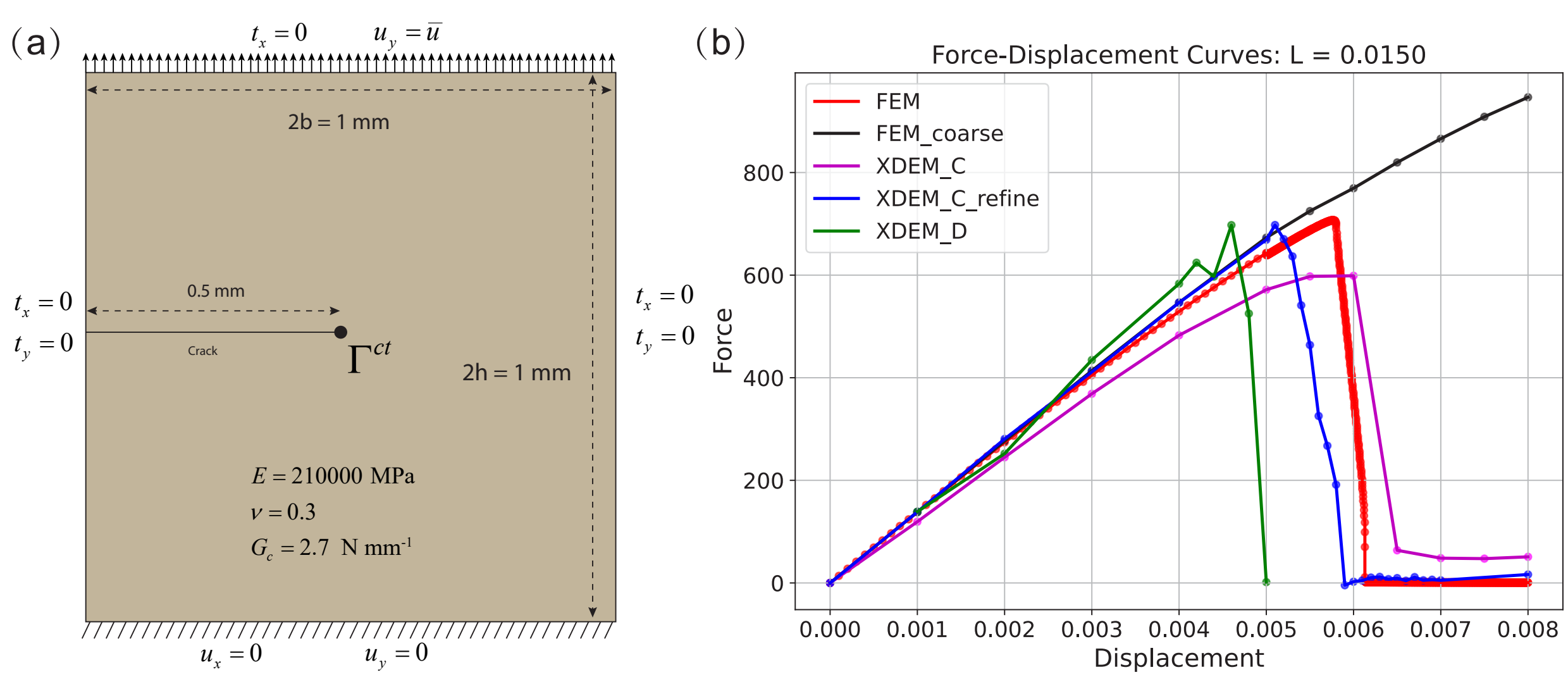

Supplementary Figure 9. Single-edge notched specimen under tensile loading: (a) Mode-I (opening) crack geometry; (b) load–displacement curves predicted by XDEM.

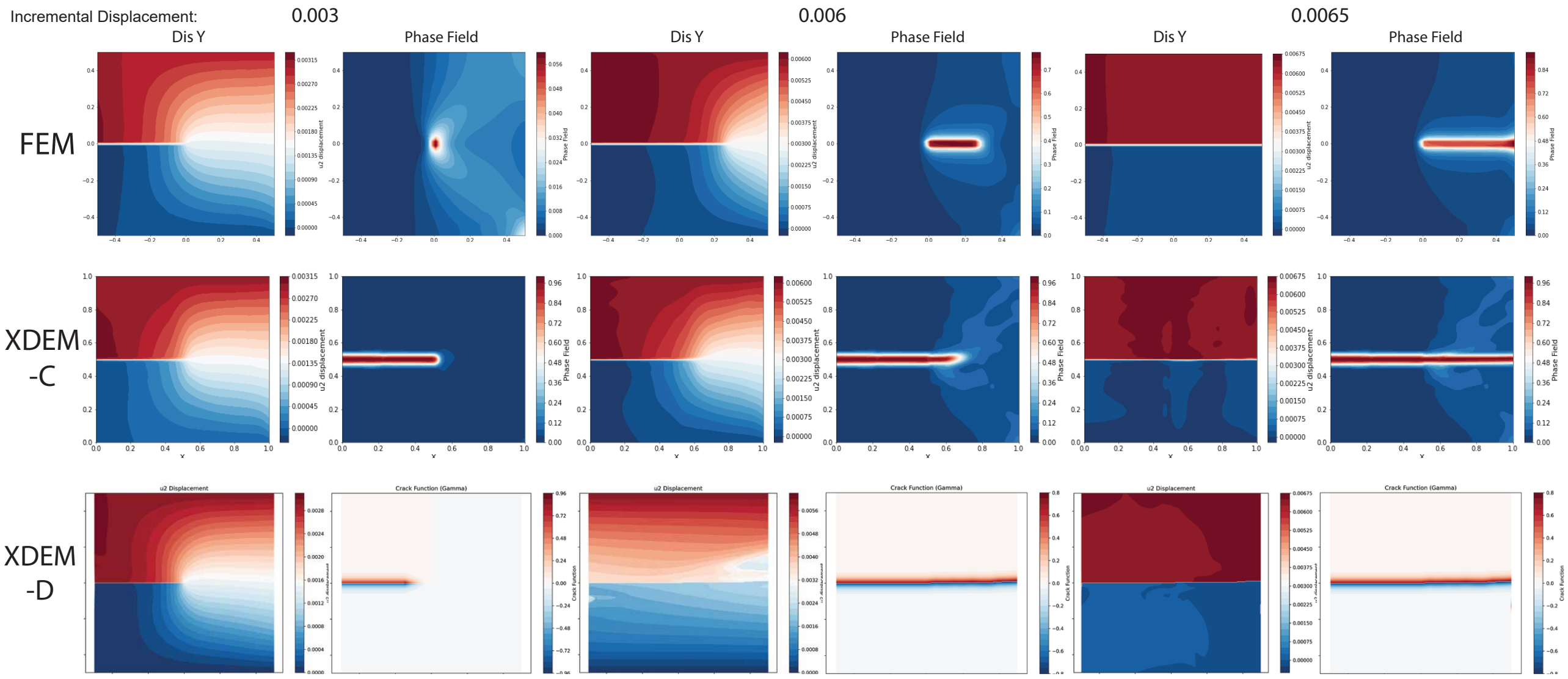

Supplementary Figure 10. Displacement and phase-field contours for the SENT test: the first and second columns show u_y and the phase field at $\bar{u} = 0.003\,\text{mm}$; the third and fourth columns at $\bar{u} = 0.006\,\text{mm}$; and the fifth and sixth columns at $\bar{u} = 0.0065\,\text{mm}$. Rows (top to bottom): FEM, XDEM-C, and XDEM-D (where the crack function replaces the phase field for crack visualization).

For the continuous model (XDEM-C), the loading is likewise partitioned into increments and the variational problem Supplementary Eq. (6) is minimized at each step. The phase-field irreversibility is enforced using a history-field approach. The displacement field is approximated by a KAN with architecture $[2,5,5,5,2]$, while the phase field is represented by an RBF network with architecture $[2,1000,1]$. We adopt a monolithic optimization strategy: the first load step is trained with 3000 Adam iterations, and subsequent steps with 1000 Adam iterations. In XDEM-C we use the AT2 model, $w(\phi) = (1-\phi)^2$, and the history-field enforcement of irreversibility.

Supplementary Fig. 9b shows the load–displacement curves predicted by XDEM. As a reference, we employ a phase-field FEM solution with length-scale $l_0 = 0.015$. Specifically, a user-defined element (UEL) implementation in ABAQUS is used with mesh refinement near the crack tip; see Supplementary Note 3.9 for details. XDEM accurately reproduces the load–displacement response using only 100×100 uniformly distributed collocation points, without the tip-focused mesh refinement required by FEM. Moreover, XDEM permits larger load increments. When FEM uses the same coarse increment as XDEM (see the "FEM_coarse" and "XDEM_C" curves in Supplementary Fig. 9b), FEM fails to capture the correct response.

Supplementary Fig. 10 presents contour plots of the displacement and phase fields predicted by XDEM, demonstrating accurate field reconstruction. Note that, in XDEM-D, the crack function serves as an indicator of the crack location: discontinuities in the crack function correspond to the crack surface.

Since XDEM-C employs an RBF network for the phase field, the centers $\mathbf{c}_i$ adaptively concentrate near the crack, as shown in Supplementary Fig. 11. More than half of the centers move toward the vicinity of the crack where gradients are steep. The associated weights w_i exhibit alternating signs, and most β_i values cluster around $1/l_0$, with higher magnitudes near the crack. We emphasize that the centers $\mathbf{c}_i$ do not lie directly on the crack, but rather in its neighborhood. This is expected because the phase field is nearly saturated (smooth) both inside the crack and far away from it (approaching 1 and 0, respectively). In contrast, the transition zone features sharp variations, and the RBF centers naturally concentrate there to allocate more basis functions where they are most needed.

XDEM in the discrete formulation faces challenges in tracking crack surfaces in three dimensions. Therefore, it is more natural and effective to employ the continuous formulation of XDEM for 3D crack problems. Here, we demonstrate the application of XDEM-C to the 3D crack configuration shown in Supplementary Fig. 12a. The phase-field length scale is chosen as $l = 0.0313$. Since the problem setup is essentially analogous to the 2D SENT test in Supplementary Fig. 9, we use the 2D FEM phase-field solution as a reference. The load–displacement response is shown in Supplementary Fig. 12b. It is worth noting that conventional DEM can only solve this problem if collocation points are heavily concentrated around the crack; with uniformly distributed points, DEM fails to converge. In contrast, XDEM achieves accurate results using far fewer, uniformly distributed collocation points. Although the accuracy of XDEM-C still shows some discrepancy compared to traditional FEM, to the best of our knowledge, the 3D load-displacement curve is the best result available in the current literature. We believe that

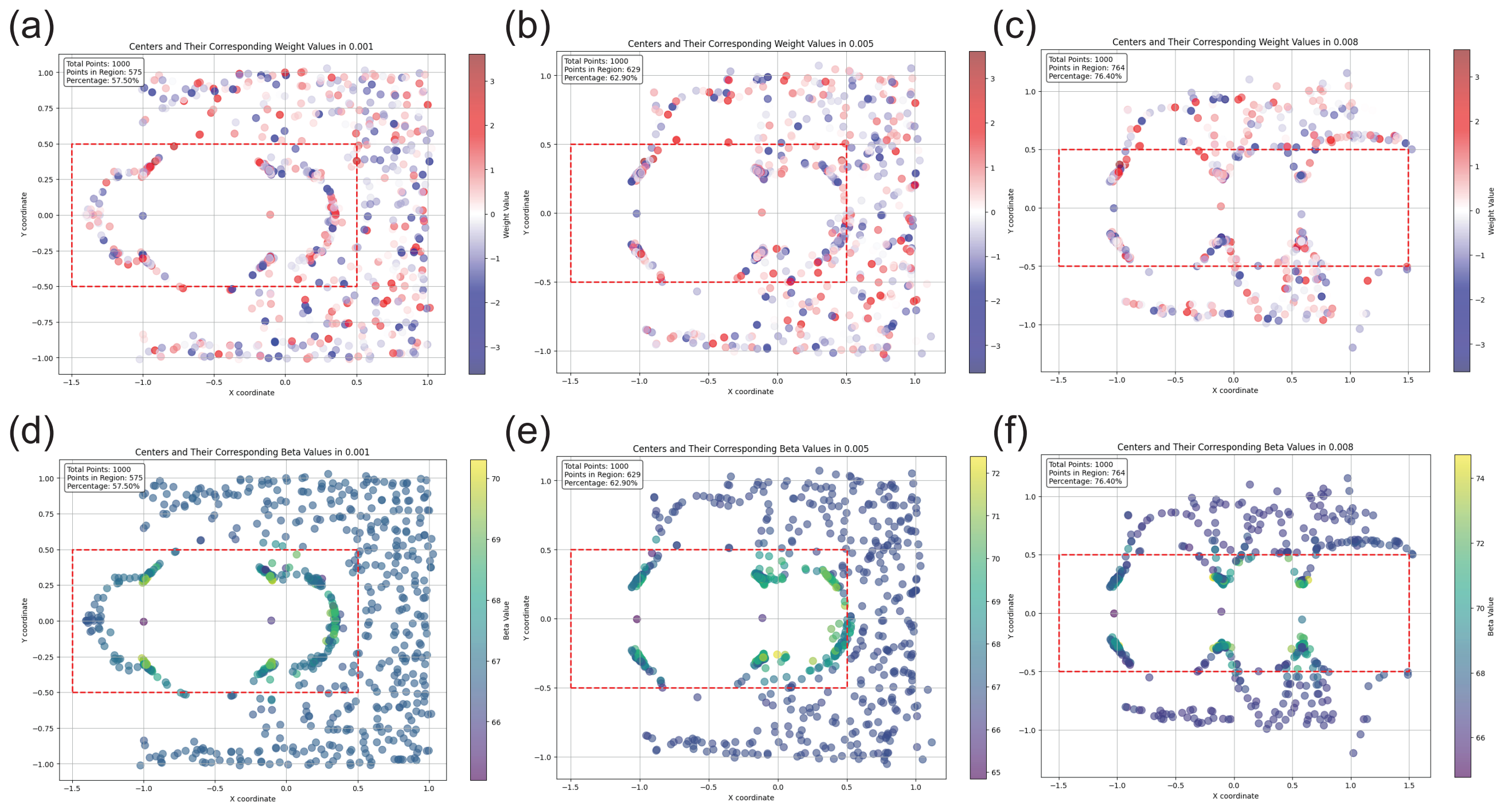

Supplementary Figure 11. Locations of RBF centers in XDEM-C at three load steps ($\bar{u} = 0.001$, 0.005, and 0.008). The first and second rows show the distributions of w_i and β_i, respectively.

XDEM-C still has room for improvement in the future, especially regarding the issue of overcoming the energy multiminima barrier in phase-field fracture, which is a key challenge for the future breakthroughs of XDEM-C.

In XDEM, collocation points are distributed uniformly: 70 points along the x- and z-directions, and 8 points along the y-direction. The displacement field is represented by a KAN network with architecture $[3,5,5,5,3]$, while the phase field is approximated by an RBF network with architecture $[3,2000,1]$. For the phase-field distribution, collocation points are placed uniformly with 20 in x, 20 in z, and 5 in y, giving a total of $20 \times 20 \times 5 = 2000$ points. A monolithic optimization strategy is adopted: the first load step is trained for 3000 Adam iterations, and subsequent steps for 1000 iterations each. Transfer learning is employed using LoRA with rank $r = 1$.

Since the energy principle requires that trial displacement fields satisfy essential boundary conditions a priori, the displacement field is constructed as

$$\begin{aligned} u_{\mathrm{x}}(x,y,z;\theta_{\mathrm{u}}) &= \mathrm{NN}_{\mathrm{x}}(x,y,z;\theta_{\mathrm{u}})\,z, \\ u_{\mathrm{y}}(x,y,z;\theta_{\mathrm{u}}) &= \mathrm{NN}_{\mathrm{y}}(x,y,z;\theta_{\mathrm{u}})\,z, \\ u_{z}(x,y,z;\theta_{\mathrm{u}}) &= \mathrm{NN}_{z}(x,y,z;\theta_{\mathrm{u}})\,z(1-z) + z\bar{u}. \end{aligned} \tag{19}$$

Supplementary Fig. 13 presents the predicted displacement and phase-field contours by XDEM. The results closely resemble those of the 2D case, further confirming the accuracy and robustness of the proposed framework in 3D fracture simulations.

2.2.2 Crack kinking

Next, we consider the crack kinking problem using XDEM. The benchmark setup is the single-edge notched specimen under shear loading, where the crack path deflects downward at approximately 70°, as shown in Supplementary Fig. 14a. The displacement field is formulated as

$$\begin{aligned} u_1(\mathbf{x},\rho;\theta_{\mathrm{u}}) &= \left(\tfrac{h+y}{2h}\right)\left(\tfrac{h-y}{2h}\right)\left[\mathrm{NN}_{\mathrm{x}}(\mathbf{x},\rho;\theta_{\mathrm{u}}) + \mathrm{T}(\mathbf{x};\Gamma^{\mathrm{ct}})\mathrm{X}_1(\mathbf{x};\Gamma^{\mathrm{ct}})\right] + \left(\tfrac{h+y}{2h}\right)\bar{u}, \\ u_2(\mathbf{x},\rho;\theta_{\mathrm{u}}) &= \left(\tfrac{h+y}{2h}\right)\left(\tfrac{h-y}{2h}\right)\left(\tfrac{b+x}{2b}\right)\left(\tfrac{b-x}{2b}\right)\left[\mathrm{NN}_{\mathrm{y}}(\mathbf{x},\rho;\theta_{\mathrm{u}}) + \mathrm{T}(\mathbf{x};\Gamma^{\mathrm{ct}})\mathrm{X}_2(\mathbf{x};\Gamma^{\mathrm{ct}})\right], \end{aligned} \tag{20}$$

where Γ^{ct} denotes the crack tip and $\bar{u}$ is the applied displacement.

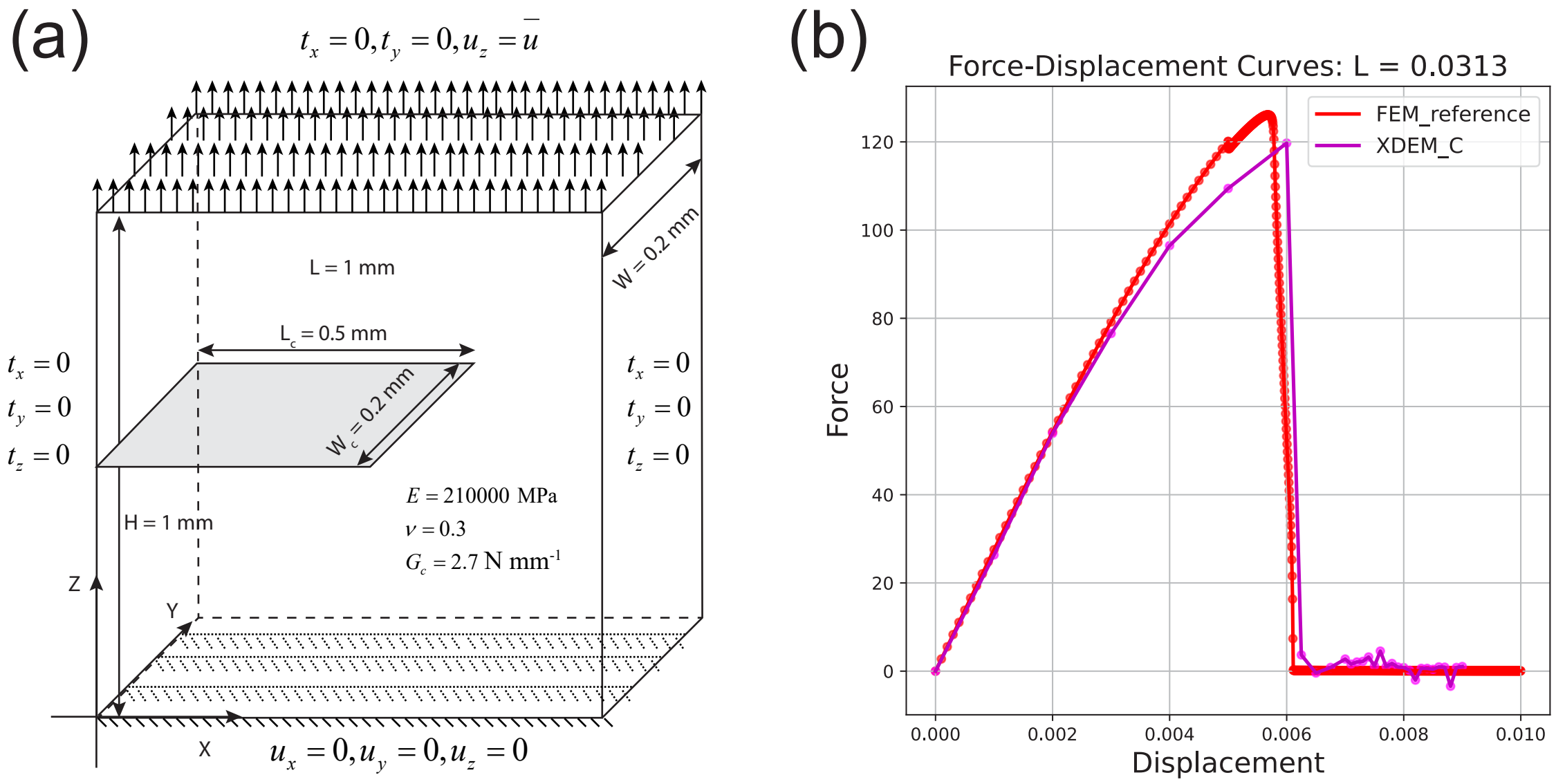

Supplementary Figure 12. (a) Geometry of the 3D crack problem. (b) Load–displacement response predicted by XDEM.

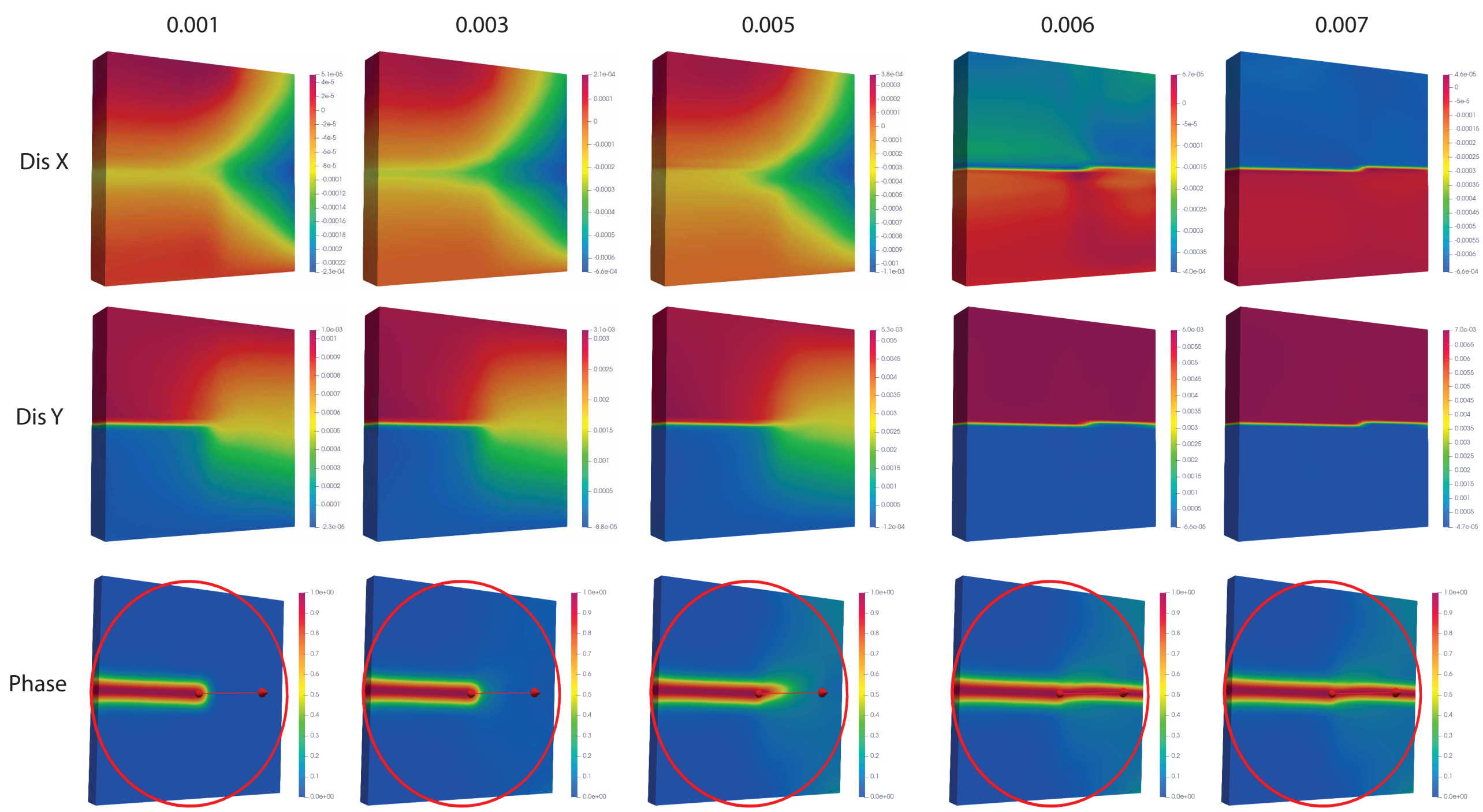

Supplementary Figure 13. XDEM predictions of displacement and phase fields in the 3D crack problem. Columns 1–6 correspond to applied displacements $\bar{u} = 0.001, 0.003, 0.005, 0.006$, and 0.007 mm, respectively. Rows (top to bottom) show u_x, u_y, and the phase field.

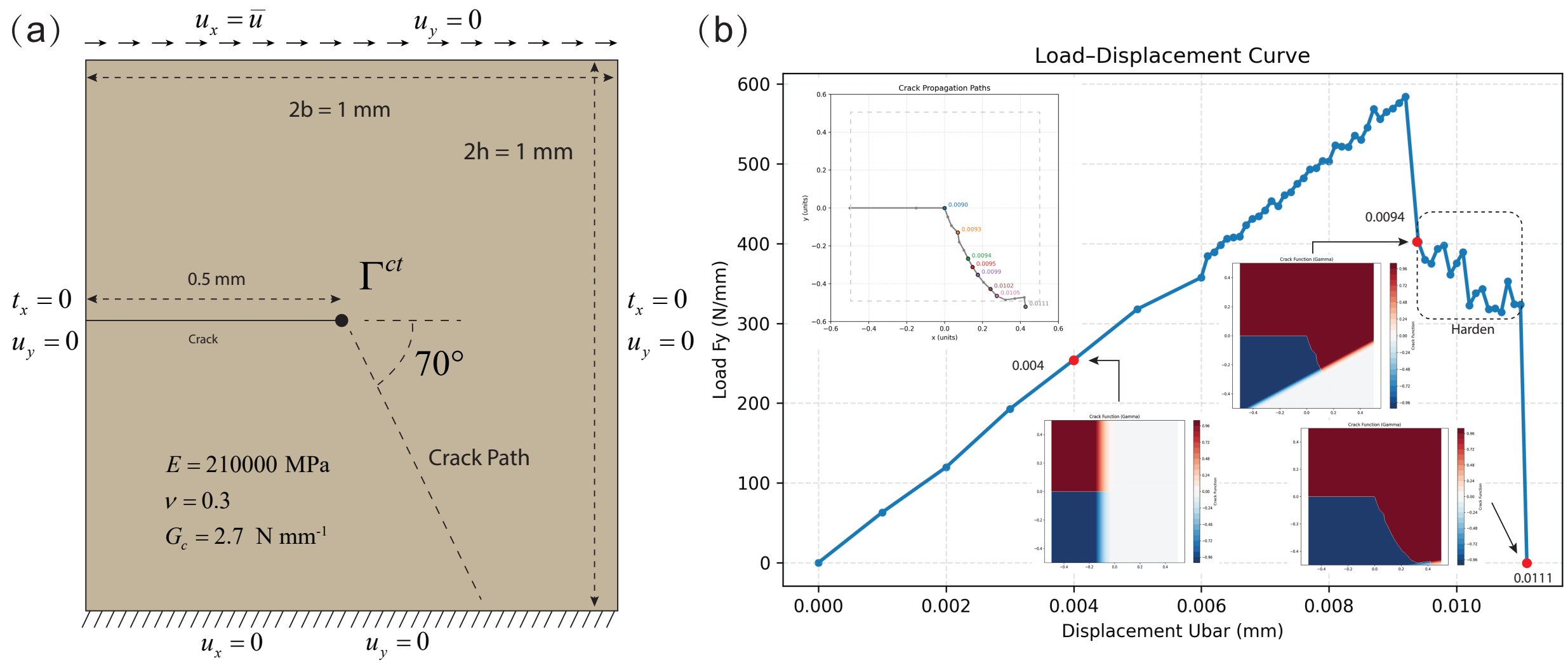

Supplementary Figure 14. Single-edge notched specimen under shear loading: (a) Mode II crack in kinking configuration; (b) Load-displacement curve predicted by XDEM.

In this case, collocation points are uniformly distributed with a resolution of 100×100. The loading increments are $\Delta\bar{u} = 0.001$ for the first six steps, followed by finer increments of $\Delta\bar{u} = 0.0001$. The load-displacement curve predicted by XDEM is shown in Supplementary Fig. 14b. We clearly observe a hardening stage, consistent with the reference results of[14]. Supplementary Fig. 14b also presents the predicted crack propagation path and the corresponding crack function under different loading steps. To demonstrate that the crack function is not unique, we also tested an alternative embedding function that satisfies Supplementary Eq. (21), incorporating an additional degradation near the crack front to better capture the interaction of multiple crack segments. The displacement and stress contours in Supplementary Fig. 15 show distinct discontinuities across the crack and stress concentration at the crack tip.

In XDEM, the crack function $\rho(\mathbf{x})$ is required to satisfy:

$$\begin{cases} \lim_{\mathbf{x}\to\mathbf{x}_c^-} \rho(\mathbf{x}) \neq \lim_{\mathbf{x}\to\mathbf{x}_c^+} \rho(\mathbf{x}), & \mathbf{x}_c \in \Gamma^c, \\ \lim_{\mathbf{x}\to\mathbf{x}_0} \rho(\mathbf{x}) = \rho(\mathbf{x}_0), & \mathbf{x}_0 \in \Omega \setminus \Gamma^c, \\ \lim_{\mathbf{x}\to\mathbf{x}_0} \nabla\rho(\mathbf{x}) = \nabla\rho(\mathbf{x}_0), & \mathbf{x}_0 \in \Omega \setminus \Gamma^c, \end{cases} \tag{21}$$

where Γ^c denotes the crack surface.

The displacement field formulation in XDEM for the Bittencourt problem is given by

$$\begin{aligned} u_1(\mathbf{x},\rho;\theta_u) &= \frac{w_2}{w_2+1}\left[\mathrm{NN_x}(\mathbf{x},\rho;\theta_u) + \mathrm{T}(\mathbf{x};\Gamma^{ct})\mathrm{X}_1(\mathbf{x};\Gamma^{ct})\right], \\ u_2(\mathbf{x},\rho;\theta_u) &= \frac{w_1 w_2 w_3}{(w_1+1)(w_2+1)}\left[\mathrm{NN_y}(\mathbf{x},\rho;\theta_u) + \mathrm{T}(\mathbf{x};\Gamma^{ct})\mathrm{X}_2(\mathbf{x};\Gamma^{ct})\right] + \frac{y+4}{8}\bar{u}, \\ w_1 &= (9+x)^2 + (4+y)^2, \\ w_2 &= (-9+x)^2 + (4+y)^2, \\ w_3 &= x^2 + (4-y)^2, \end{aligned} \tag{22}$$

where Γ^{ct} denotes the crack tip and $\bar{u}$ is the applied displacement.

2.2.3 Crack inclusion

The displacement field is assumed as

$$\begin{aligned} u_1(\mathbf{x},\rho;\theta_u) &= \left(\frac{4+y}{8}\right)\left[\mathrm{NN_x}(\mathbf{x},\rho;\theta_u) + \mathrm{T}(\mathbf{x};\Gamma^{ct})\mathrm{X}_1(\mathbf{x};\Gamma^{ct})\right], \\ u_2(\mathbf{x},\rho;\theta_u) &= \left(\frac{4+y}{8}\right)\left(\frac{4-y}{8}\right)\left[\mathrm{NN_y}(\mathbf{x},\rho;\theta_u) + \mathrm{T}(\mathbf{x};\Gamma^{ct})\mathrm{X}_2(\mathbf{x};\Gamma^{ct})\right] + \left(\frac{4+y}{8}\right)\bar{u}. \end{aligned} \tag{23}$$

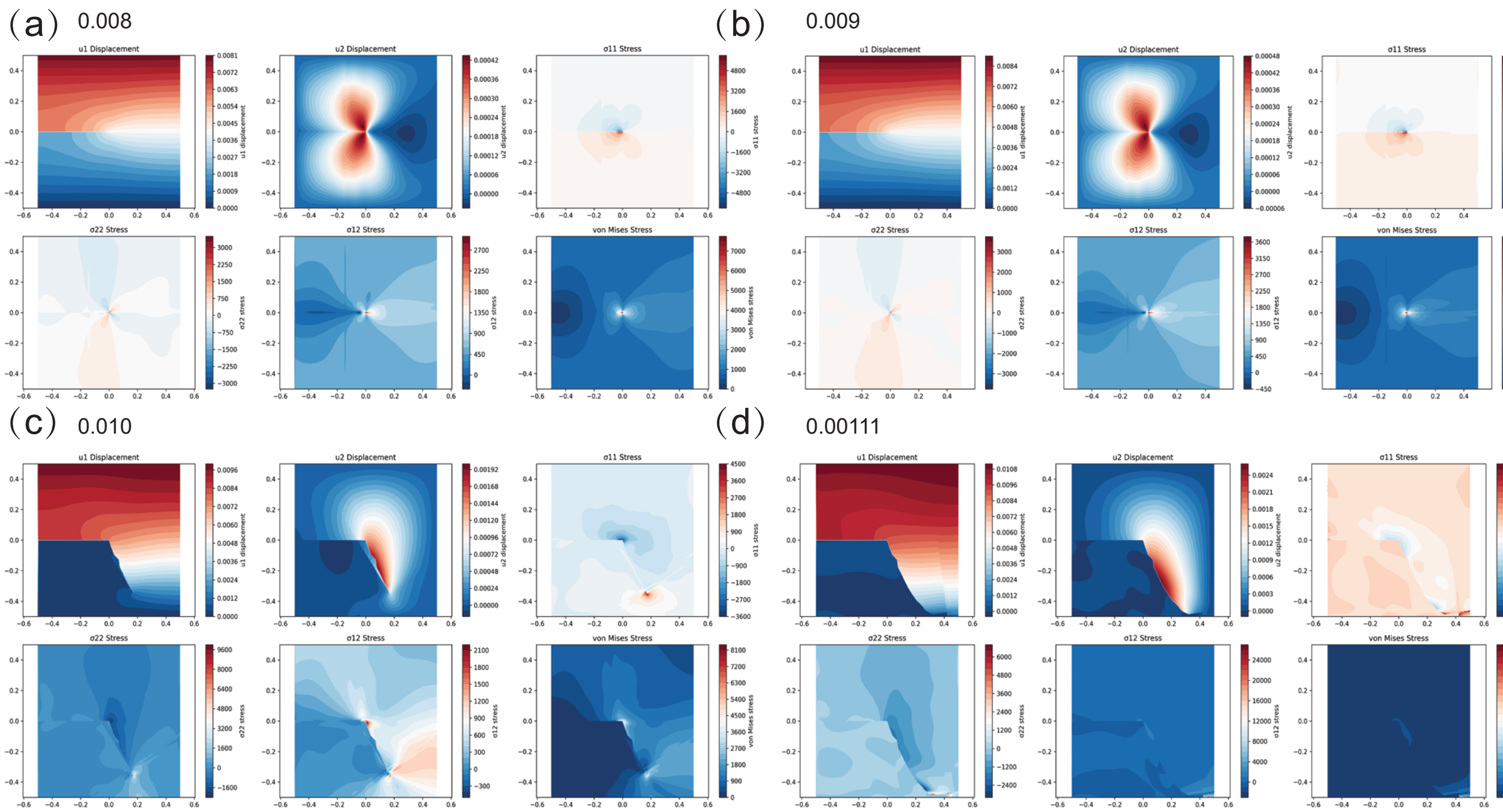

Supplementary Figure 15. Displacement and stress contours predicted by XDEM for the single-edge notched specimen under shear loading at different displacement levels. The applied displacement $\bar{u} = 0.00111$ corresponds to complete failure.

2.2.4 Crack initiation

Crack initiation problems are often difficult to handle using discrete crack models. Therefore, we employ the continuous XDEM formulation to address this case, as illustrated in Supplementary Fig. 16a. The displacement field setup follows[15]. We employ a penalization term to enforce the irreversibility of the phase field.

Supplementary Fig. 16b presents the evolution of the total energy functional and the phase-field contours at different loading levels. The results predicted by XDEM show very good agreement with the reference solution of[15]. One notable advantage of XDEM is its flexibility in network selection. In this example, we approximate both the displacement and phase-field using Kolmogorov Arnold Networks (KANs) with architecture $[2,15,15,15,3]$. For differentiation, we employed finite element shape function derivatives rather than automatic differentiation (AD) algorithms. It is worth noting that XDEM only used 7700 uniformly distributed collocation points, which is significantly fewer than the 34149 points used by[15], who further required local mesh refinement in the crack initiation region.

Supplementary Fig. 16 c and d show the predicted displacement and stress fields at displacement loads $\bar{u} = 0.1$ and $\bar{u} = 1.2$, respectively. These results demonstrate that XDEM is able to capture the nucleation and subsequent evolution of cracks with high accuracy and efficiency.

Supplementary Note 3 Discussion

3.1 Zero-energy modes in DEM: Non-physical (virtual) fracture

During our experiments, we observed that when the number of collocation points in DEM is insufficient, spurious non-physical fracture patterns may appear. An example is shown in Supplementary Fig. 17a, where the y-displacement field under mode I loading exhibits an artificial discontinuity. In the following, we explain the cause of this numerical artifact.

Such virtual fracture is a common failure mode of DEM training. To illustrate the mechanism, let us first consider a simple linear elastic problem. Under external loading (i.e., Neumann boundary conditions), if there is a mismatch between the strain energy and the external work in the total potential, the strain energy is often underestimated. As a result, the neural network tends to focus on minimizing the external potential, whose gradient dominates and overwhelms the contribution from the internal energy. Consequently, displacements on the Neumann boundary may grow excessively in order to reduce the overall energy. In principle, the internal strain energy should also increase, but due to inaccurate integration between boundary collocation points and interior points near the boundary, the strain energy contribution is underestimated. This creates a loophole where the neural network artificially reduces the energy by introducing displacement jumps at the boundary.

In the case of pure displacement loading (Dirichlet boundary conditions), the external work vanishes, and the network

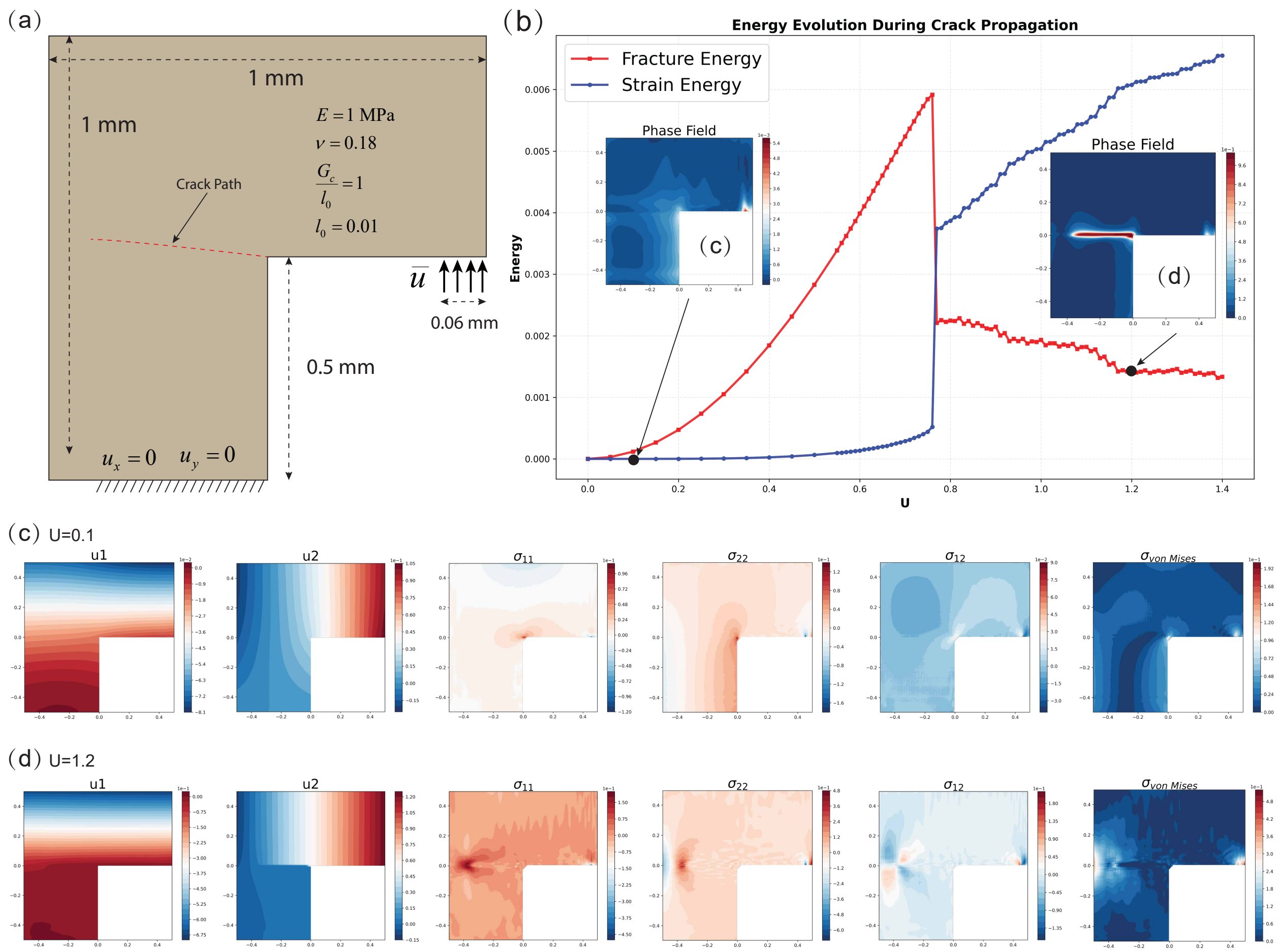

Supplementary Figure 16. Performance of XDEM in the crack initiation problem: (a) schematic of the setup; (b) evolution of the energy functional and phase-field distribution; (c) displacement and stress fields at $\bar{u}=0.1$; (d) displacement and stress fields at $\bar{u}=1.2$.

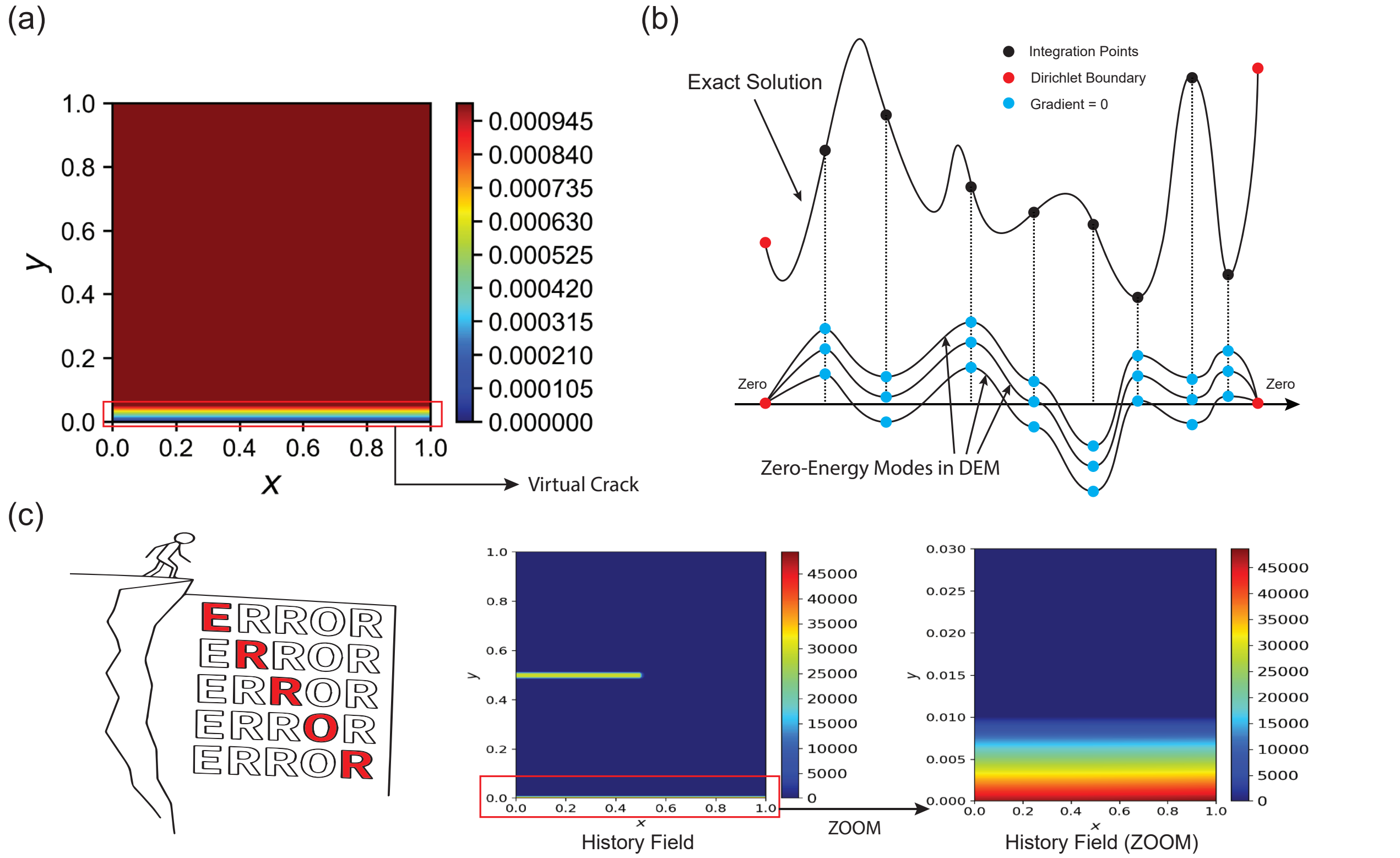

Supplementary Figure 17. Non-physical fracture phenomenon: (a) DEM failure with insufficient collocation points, showing artificial discontinuities at the boundary; (b) illustration of zero-energy modes in DEM; (c) Illustration of the failure cascade caused by zero-energy modes in XDEM. The cliffside metaphor represents a numerically unstable state in which small non-physical errors can be amplified and inherited by subsequent load steps. The history-field maps show that a virtual crack may be retained in the history variable and further affect the predicted crack evolution.

focuses solely on minimizing strain energy. When integration accuracy is insufficient, the network tends to approximate the displacement field as uniformly as possible in the domain, which again may lead to spurious fracture-like discontinuities, as illustrated in Supplementary Fig. 17a.

Fundamentally, this phenomenon can be interpreted as a manifestation of zero-energy modes, as illustrated in Supplementary Fig. 17b:

$$\Pi(u^{\text{exact}}) = \Pi(u^{\text{exact}} + u^{\text{zero}}), \tag{24}$$

where u^{zero} denotes a zero-energy mode displacement field. In this mode, the strain (derivatives) vanishes at collocation points, yet may vary arbitrarily in between. At essential boundary points, the displacements remain zero (here we only consider displacement fields). Such u^{zero} can be added to the exact solution u^{exact} without affecting the energy functional Π, and hence cannot be eliminated by the optimizer. Unlike finite element methods, where spurious zero-energy modes are finite-dimensional, the zero-energy modes in DEM are essentially infinite-dimensional and thus more difficult to control.

In fracture simulations, the impact of such spurious modes becomes magnified. Once a non-physical crack emerges, it contaminates all subsequent load steps, as illustrated in Supplementary Fig. 17c, leading to cascading failure. A practical remedy is to reinitialize the optimization once such virtual fracture is detected.

In summary, the root cause of virtual fracture is insufficient integration accuracy, which typically occurs when the number of collocation points is too small relative to the number of training iterations. With too few points, numerical integration becomes inaccurate, and excessive training steps make the zero-energy mode more likely to appear. The recommended solution is to maintain a reasonable proportionality between the number of iterations and the number of collocation points.

3.2 Loss functions in DEM and XDEM

In the experiments presented in Supplementary Note 2.1.1, we observed that XDEM predicts stress intensity factors (SIFs) more accurately and converges faster than DEM. A natural question is whether the evolution of the energy function (loss

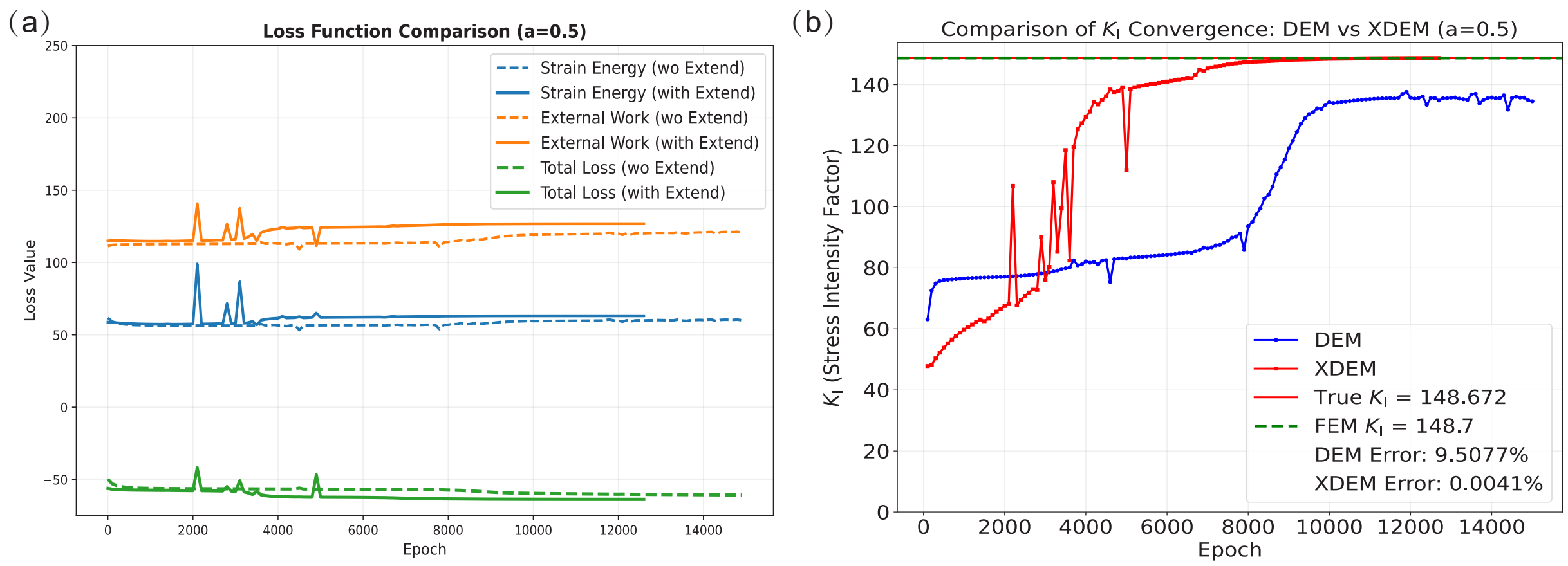

Supplementary Figure 18. Evolution of the loss function and stress intensity factors in DEM and XDEM.

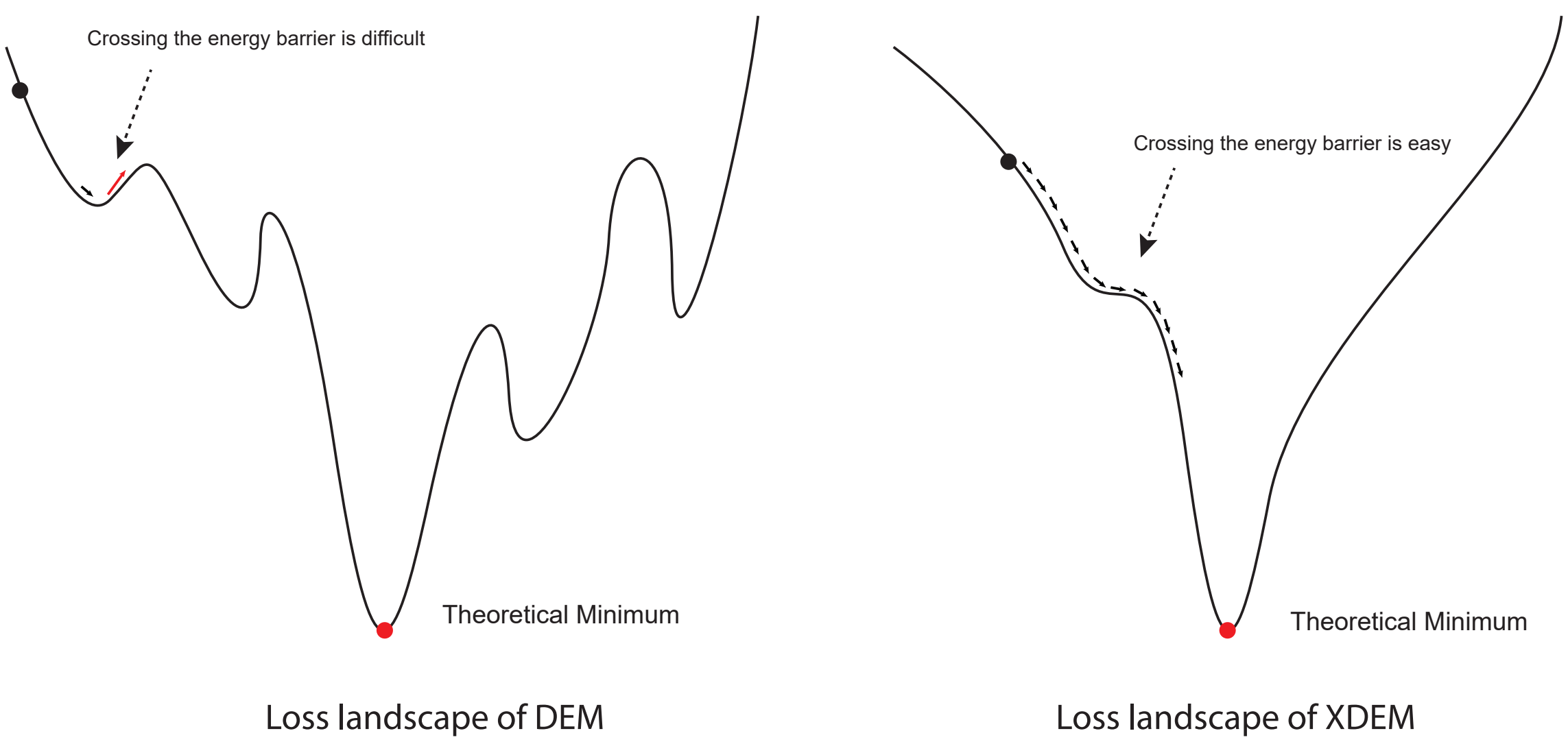

Supplementary Figure 19. Comparison of the loss landscapes between DEM and XDEM. Note that this is only a schematic.

landscape) differs significantly between DEM and XDEM. To answer this, we analyzed the loss functions from the same setting as in Supplementary Fig. 4a. As shown in Supplementary Fig. 18, the overall difference in energy loss between DEM and XDEM is not substantial. However, their abilities to capture the SIF differ markedly. This discrepancy arises because the SIF characterizes a local physical quantity at the crack tip, which has limited impact on the global energy. This again confirms the role of the extended function in XDEM: it enhances the representation of crack-tip fields and allows XDEM to more effectively capture local features relevant to fracture.

3.3 Message passing

The inclusion of extended functions in XDEM significantly improves its accuracy and efficiency. In principle, neural networks are universal approximators with strong representation capabilities[21]. Thus, even without extended functions, the network could eventually achieve comparable results if trained with a sufficient number of iterations. In practice, however, PINNs often fail to reach this potential, not because of inadequate expressive power, but due to optimization difficulties.

In XDEM, we alleviate this issue by introducing the crack function to approximate displacement discontinuities, and the extended function to represent crack-tip fields. These additions effectively reduce the non-convexity of the loss landscape, thereby easing the optimization process. We refer to this phenomenon as *message passing*, as illustrated in Supplementary Fig. 19.

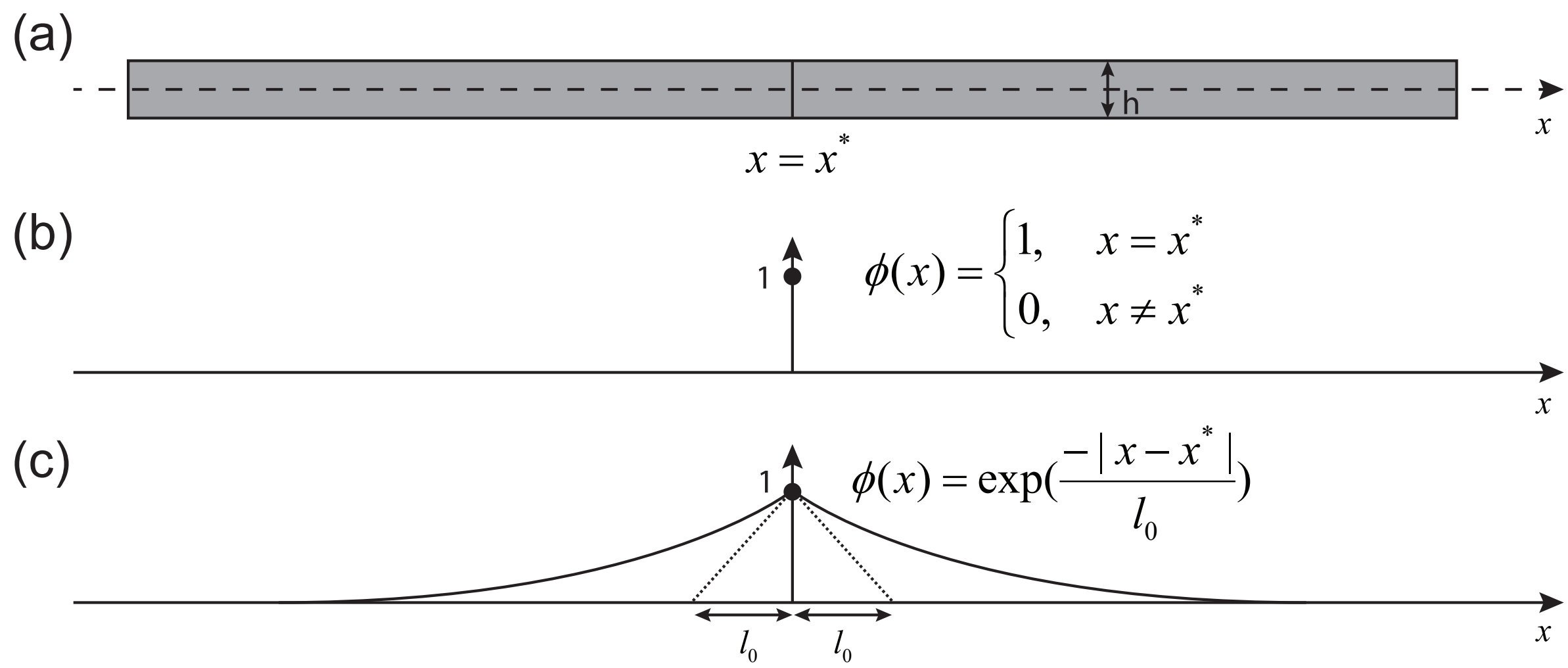

Supplementary Figure 20. Phase-field representation of a one-dimensional infinite crack: (a) schematic of a one-dimensional infinite crack, where x^* is the crack location; (b) discrete phase-field representation; (c) diffuse phase-field representation.

3.4 Irreversibility of the Phase Field

The irreversibility of the phase field is typically enforced in two ways: the penalization approach and the history field approach:

$$\begin{aligned} \text{Penalization: } U^{\text{ir}} &= \int_\Omega \frac{1}{2}\gamma_{ir}\langle \phi - \phi^n \rangle_-^2 \, dV, \\ \text{History: } U^{\text{ir}} &= \int_\Omega w(\phi) H(\Psi^+, t_n) \, dV, \end{aligned} \tag{25}$$

where γ_{ir} is the penalty factor, which must be prescribed a priori. Following[8], it can be defined as:

$$\gamma_{ir} = \begin{cases} \frac{G_c}{l_0}\frac{27}{64\delta_{tol}^2}, & \text{AT1}, \\ \frac{G_c}{l_0}\left(\frac{1}{\delta_{tol}^2} - 1\right), & \text{AT2}, \end{cases} \tag{26}$$

with δ_{tol} denoting the tolerance parameter.

In the history field formulation, H is given by:

$$H(\Psi^+, t) = \max_{\tau \in [0,t]} \Psi^+(\boldsymbol{\varepsilon}(\mathbf{x}, \tau)), \tag{27}$$

where Ψ^+ is the tensile part of the elastic energy density. For problems with an initial crack, the history field is initialized as:

$$H(\Psi^+, t = 0) = \begin{cases} \frac{BG_c}{2l_0}\left[1 - \frac{2d(\mathbf{x})}{l_0}\right], & d(\mathbf{x}) \le \frac{l_0}{2}, \\ 0, & d(\mathbf{x}) > \frac{l_0}{2}, \end{cases} \tag{28}$$

where B is a tunable hyperparameter (set to 1000 in our implementation), and $d(\mathbf{x})$ denotes the shortest distance from point $\mathbf{x}$ to the crack surface.

3.5 The Phase Field in Fracture

We illustrate the similarity between the phase field in fracture and the radial basis function (RBF) space using a one-dimensional infinite crack example, as shown in Supplementary Fig. 20.

Since the crack exists only at $x = x^*$, the crack can be described by a discrete phase field:

$$\phi(x) = \begin{cases} 1, & x = x^*, \\ 0, & x \neq x^*. \end{cases} \tag{29}$$

Although this description is exact, it causes difficulties in numerical simulations. Therefore, we relax Supplementary Eq. (29) into a smooth formulation:

$$\phi(x) = \exp\left(-\frac{|x-x^*|}{l_0}\right), \tag{30}$$

where l_0 is the length scale parameter controlling the extent of the diffused crack zone. The larger the l_0, the wider the diffuse region of the crack.

It is straightforward to verify that Supplementary Eq. (30) is the solution of the following ordinary differential equation (ODE):

$$\begin{aligned} &\text{ODE: } \phi(x) - l_0^2\phi''(x) = 0, \\ &\text{Boundary: } \phi(\pm\infty) = 0, \quad \phi(x^*) = 1, \end{aligned} \tag{31}$$

which corresponds to a Helmholtz-type equation with prescribed boundary conditions.

By employing the weak form and Gaussian quadrature, the solution of Supplementary Eq. (31) can be reformulated as a minimization problem of the functional I:

$$\begin{aligned} &\phi(x) = \arg\min_{\phi(x)\in C_\mathrm{b}} I(\phi), \\ &I(\phi) = A_\Gamma \int_{-\infty}^{+\infty} \frac{1}{2}\left(\phi^2(x) + l_0^2[\phi'(x)]^2\right) dx, \end{aligned} \tag{32}$$

where $A_\Gamma = h$, with h denoting the height of the one-dimensional bar (see Supplementary Fig. 20a). The admissible space is $C_\mathrm{b} = \{\phi(x) \,|\, \phi(\pm\infty) = 0,\ \phi(x^*) = 1\}$, i.e., the set of all phase-field functions satisfying the boundary conditions in Supplementary Eq. (31).

It is evident that Supplementary Eq. (30) closely resembles the RBF formulation introduced in Supplementary Note 3.6, which further explains the suitability of RBF networks for approximating the phase field in fracture.

3.6 Radial Basis Networks and Kolmogorov-Arnold Networks

3.6.1 Radial Basis Networks for the Phase Field

The structure of a radial basis function (RBF) network can be expressed as

$$\phi(\mathbf{x};\theta_\phi) = \mathrm{R}(\mathbf{x};\theta_\phi) = \sum_{i=1}^{N} w_i \exp[-\beta_i(\mathbf{x}-\mathbf{c}_i)\cdot(\mathbf{x}-\mathbf{c}_i)], \tag{33}$$

where w_i, β_i, and $\mathbf{c}_i$ are trainable network parameters.

In practice, we adopt a slightly modified form by introducing normalization at the output:

$$\phi(\mathbf{x};\theta_\phi) = R(\mathbf{x};\theta_\phi) = \frac{\sum_{i=1}^{N} w_i \exp[-\beta_i(\mathbf{x}-\mathbf{c}_i)\cdot(\mathbf{x}-\mathbf{c}_i)]}{\sum_{i=1}^{N} \exp[-\beta_i(\mathbf{x}-\mathbf{c}_i)\cdot(\mathbf{x}-\mathbf{c}_i)]}. \tag{34}$$

The normalized form Supplementary Eq. (34) has the advantage of being able to represent constant functions simply by setting all w_i equal, which is not possible in Supplementary Eq. (33). This property is particularly important for fracture phase-field modeling, where the field is zero over most of the domain. Hence, the normalized RBF form is more consistent with the intrinsic distribution of the phase field.

3.6.2 Kolmogorov-Arnold Networks for the Displacement Field

In Kolmogorov-Arnold Networks (KANs)[22], consider a layer with l_i input neurons and l_o output neurons. The activation functions of this layer are denoted by ϕ_{ij}, where $i \in \{1,2,\cdots,l_o\}$ and $j \in \{1,2,\cdots,l_i\}$. Each activation ϕ_{ij} is constructed from B-spline basis functions according to the number of grid points G and the spline order r:

$$\phi_{ij}(\mathbf{X}) = \begin{bmatrix} \sum_{m=1}^{G_1+r_1} c_m^{(1,1)} B_m(x_1) & \sum_{m=1}^{G_2+r_2} c_m^{(1,2)} B_m(x_2) & \cdots & \sum_{m=1}^{G_{l_i}+r_{l_i}} c_m^{(1,l_i)} B_m(x_{l_i}) \\ \sum_{m=1}^{G_1+r_1} c_m^{(2,1)} B_m(x_1) & \sum_{m=1}^{G_2+r_2} c_m^{(2,2)} B_m(x_2) & \cdots & \sum_{m=1}^{G_{l_i}+r_{l_i}} c_m^{(2,l_i)} B_m(x_{l_i}) \\ \vdots & \vdots & \ddots & \vdots \\ \sum_{m=1}^{G_1+r_1} c_m^{(l_o,1)} B_m(x_1) & \sum_{m=1}^{G_2+r_2} c_m^{(l_o,2)} B_m(x_2) & \cdots & \sum_{m=1}^{G_{l_i}+r_{l_i}} c_m^{(l_o,l_i)} B_m(x_{l_i}) \end{bmatrix}, \tag{35}$$

Supplementary Table 2
Trainable parameters in KAN.

Type of parameters	Variable	Number	Description
$c_m^{(i,j)}$	spline_weight	$l_o \times l_i \times (G+r)$	Coefficients of B-spline in activation function ϕ
W_{ij}	base_weight	$l_i \times l_o$	Linear transformation after nonlinear activation σ
S_{ij}	spline_scaler	$l_i \times l_o$	Scaling factors for activation functions

where G_j is the number of grid points and r_j is the order of the B-spline in the j-th input direction. Each coefficient $c_m^{(i,j)}$ corresponds to a B-spline weight, with $(G_j + r_j)$ parameters in the j-th direction. Note that both the grid division and spline order can be chosen independently for each input direction.

To enhance the representational power of the activation functions, we introduce scaling matrices S_{ij} with the same shape as ϕ_{ij}:

$$S_{ij} = \begin{bmatrix} s_{11} & s_{12} & \cdots & s_{1l_i} \\ s_{21} & s_{22} & \cdots & s_{2l_i} \\ \vdots & \vdots & \ddots & \vdots \\ s_{l_o1} & s_{l_o2} & \cdots & s_{l_ol_i} \end{bmatrix}. \tag{36}$$

The scaling operation is applied element-wise as $\phi = \phi \odot \mathbf{S}$, where $\odot$ denotes the Hadamard product.

The final output of the layer is given by

$$\mathbf{Y} = \tanh\left\{ \sum_{\text{columns}} [\phi(\mathbf{X}) \odot \mathbf{S}] + \mathbf{W} \cdot \sigma(\mathbf{X}) \right\}, \tag{37}$$

where $\mathbf{W}$ is a linear transformation matrix and σ is a nonlinear activation function. The residual term $\mathbf{W} \cdot \sigma(\mathbf{X})$ plays a role analogous to ResNet[23], while the scaling factors $\mathbf{S}$ and $\mathbf{W}$ act similarly to normalization layers. The additional nonlinear mapping σ improves smoothness, preventing the B-spline approximation from producing rough outputs.

If the grid and spline order are the same for all input directions, the number of trainable parameters $c_m^{(i,j)}$ is $(G+r)$ for each direction. The total number of trainable parameters in KAN is summarized in Supplementary Table 2. Further details can be found in the original KAN paper[22] and its applications to PINNs, such as KINN[18,24].

3.7 Monolithic and Staggered Schemes

We derive the first variation of the AT2 model in Supplementary Eq. (3) as

$$\begin{aligned} \delta\Pi = & \int_\Omega \left\{ w'(\phi)\Psi^+(\boldsymbol{\varepsilon}) + \frac{G_c}{l_0}(\phi - l_0^2\phi_{,ii}) + w'(\phi)H(\Psi^+) \right\} \delta\phi \, dV \\ & - \int_\Omega \left\{ [w(\phi)\sigma_{ij}^+ + \sigma_{ij}^-]_{,j} + f_i \right\} \delta u_i \, dV + \int_{\Gamma^t} \left\{ [w(\phi)\sigma_{ij}^+ + \sigma_{ij}^-] n_j - \bar{t}_i \right\} \delta u_i \, dS \\ & + \int_\Gamma l_0^2 \phi_{,i} n_i \delta\phi \, dS, \end{aligned} \tag{38}$$

where $\delta H(\Psi^+) = 0$.

The second variation of Supplementary Eq. (38) is

$$\begin{aligned} \delta^2\Pi = & \int_\Omega \Big[w''(\phi)\delta\phi^2\Psi^+(\boldsymbol{\varepsilon}) + 2w'(\phi)\delta\phi\sigma_{ij}^+(\boldsymbol{\varepsilon})\delta\varepsilon_{ij} + w(\phi)\delta\varepsilon_{kl}\frac{\partial^2\Psi^+}{\partial\varepsilon_{ij}\partial\varepsilon_{kl}}\delta\varepsilon_{ij} \\ & + \delta\varepsilon_{kl}\frac{\partial^2\Psi^-}{\partial\varepsilon_{ij}\partial\varepsilon_{kl}}\delta\varepsilon_{ij} \Big] dV \\ & + \int_\Omega \left\{ \frac{G_c}{l_0}\left[\delta\phi^2 + l_0^2(\nabla\delta\phi)\cdot(\nabla\delta\phi)\right] + w''(\phi)\delta\phi^2 H(\Psi^+) \right\} dV. \end{aligned} \tag{39}$$

We assume $w(\phi) = (1-\phi)^2$. It can be shown that for any nonzero $\delta\boldsymbol{\varepsilon}$,

$$\begin{aligned} & w(\phi)\delta\varepsilon_{kl}\frac{\partial^2\Psi^+}{\partial\varepsilon_{ij}\partial\varepsilon_{kl}}\delta\varepsilon_{ij} + \delta\varepsilon_{kl}\frac{\partial^2\Psi^-}{\partial\varepsilon_{ij}\partial\varepsilon_{kl}}\delta\varepsilon_{ij} \\ & = w(\phi)\left[\lambda\langle\delta\varepsilon_{ii}\rangle_+^2 + 2\mu\langle\delta\varepsilon_{ij}\rangle_+^2\right] + \left[\lambda\langle\delta\varepsilon_{ii}\rangle_-^2 + 2\mu\langle\delta\varepsilon_{ij}\rangle_-^2\right] \geq 0. \end{aligned} \tag{40}$$

Thus, the only term affecting the positive definiteness of $\delta^2\Pi$ in Supplementary Eq. (39) is

$$\int_\Omega 2w^{'}(\phi)\delta\phi\sigma_{ij}^{+}(\boldsymbol{\varepsilon})\delta\varepsilon_{ij}.$$

Rewriting Supplementary Eq. (39) in matrix form yields

$$\delta^2\Pi = \int_\Omega \begin{bmatrix}\delta\boldsymbol{\varepsilon}\\ \delta\phi\end{bmatrix}^T \begin{bmatrix}\mathbf{A} & \mathbf{B}\\ \mathbf{B}^T & C\end{bmatrix} \begin{bmatrix}\delta\boldsymbol{\varepsilon}\\ \delta\phi\end{bmatrix} dV, \tag{41}$$

with

$$\begin{aligned}
&\mathbf{A} = w(\phi)\frac{\partial^2\Psi^+}{\partial\boldsymbol{\varepsilon}\partial\boldsymbol{\varepsilon}} + \frac{\partial^2\Psi^-}{\partial\boldsymbol{\varepsilon}\partial\boldsymbol{\varepsilon}},\\
&\mathbf{B} = \sigma^+(\boldsymbol{\varepsilon})w^{'}(\phi),\\
&C = w^{''}(\phi)[\Psi^+(\boldsymbol{\varepsilon}) + H(\Psi^+)] + \frac{G_c}{l_0}\left[1 + l_0^2(\nabla)\cdot(\nabla)\right].
\end{aligned} \tag{42}$$

For $\delta\boldsymbol{\varepsilon} = A^{-1}B\delta\phi$, the minimization becomes

$$\min_{\delta\boldsymbol{\varepsilon}}(\delta^2\Pi) = \delta\phi^T M\delta\phi, \quad M = C - \mathbf{B}^T\mathbf{A}^{-1}\mathbf{B}. \tag{43}$$

Hence, a necessary and sufficient condition for $\delta^2\Pi \geq 0$ is $M > 0$, i.e.,

$$w^{''}(\phi)\left[\Psi^+(\boldsymbol{\varepsilon}) + H(\Psi^+)\right] + \frac{G_c}{l_0}\left[1 + l_0^2(\nabla)\cdot(\nabla)\right] > [w^{'}(\phi)]^2\boldsymbol{\sigma}^+(\boldsymbol{\varepsilon})\left[w(\phi)\frac{\partial^2\Psi^+}{\partial\boldsymbol{\varepsilon}\partial\boldsymbol{\varepsilon}} + \frac{\partial^2\Psi^-}{\partial\boldsymbol{\varepsilon}\partial\boldsymbol{\varepsilon}}\right]^{-1}\boldsymbol{\sigma}^+(\boldsymbol{\varepsilon}). \tag{44}$$

From Supplementary Eq. (44), we conclude:

- At early loading stages, Supplementary Eq. (44) always holds since $\boldsymbol{\varepsilon} = 0$ initially, making the right-hand side vanish. Physically, the system is stable with no crack initiation.
- At the critical load, Supplementary Eq. (44) reduces to an equality, corresponding to $\delta^2\Pi \geq 0$ and the onset of crack nucleation.
- Under overload, Supplementary Eq. (44) is violated, implying instability and crack propagation.

Therefore, algorithmically, when the load is below the crack initiation threshold, ϕ and $\mathbf{u}$ can be optimized simultaneously using a monolithic scheme for faster convergence. Near the critical load, a staggered scheme is preferable since the cross term $\int_\Omega 2w^{'}(\phi)\delta\phi\sigma_{ij}^{+}(\boldsymbol{\varepsilon})\delta\varepsilon_{ij} = 0$, ensuring $\delta^2\Pi \geq 0$ and thus improved robustness. As shown in Supplementary Fig. 21, the energy landscape varies across different loading stages.

3.8 Calculation of Stress Intensity Factor

In XDEM, two approaches are employed to evaluate the stress intensity factors (SIFs): the J-integral method and the interaction integral method.

First, consider the J-integral, defined as

$$J = \oint_\Gamma (wn_1 - n_\alpha\sigma_{\alpha\beta}u_{\beta,1})d\Gamma \tag{45}$$

where w is the strain energy density function, n_1 is the x-component of the unit normal vector to the closed contour Γ in the local coordinate system, $\sigma_{\alpha\beta}$ is the stress tensor, and $u_{\beta,1}$ denotes the gradient of the displacement in the local x-direction. All physical quantities must be evaluated in the local coordinate system, with the x-axis aligned with the crack tangent. The contour Γ encloses the crack tip.

In linear elastic fracture mechanics (LEFM), the J-integral represents the energy release rate:

$$J = G_c = \frac{K_I^2}{E} + \frac{K_{II}^2}{E} + \frac{K_{III}^2}{2G}, \tag{46}$$

where K_I, K_{II}, and K_{III} denote the mode I, mode II, and mode III SIFs, respectively. E and G are the elastic and shear moduli. For plane strain conditions, E is replaced by $E/(1-\nu^2)$, where ν is the Poisson's ratio.

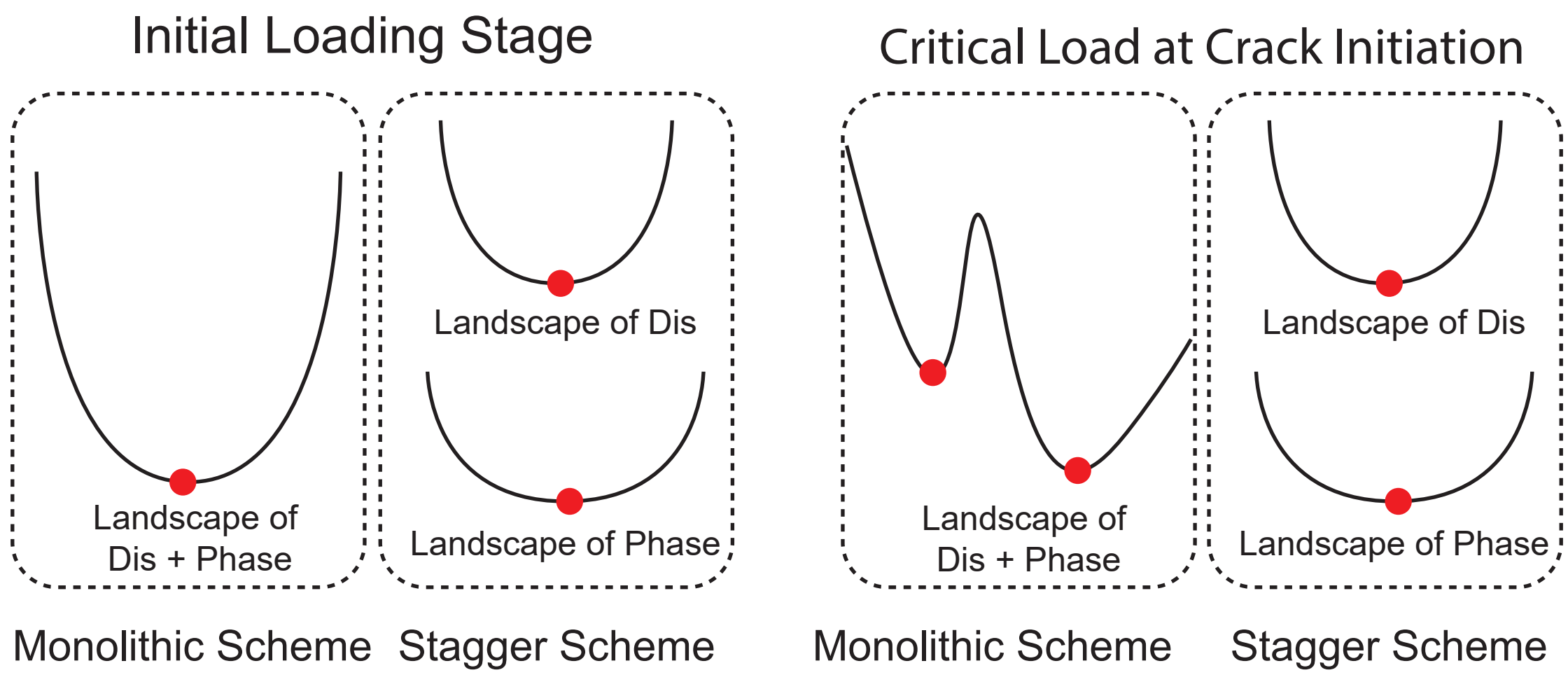

Supplementary Figure 21. Energy landscape under different load levels. Red dots indicate minima. Left: Early loading stage, where both monolithic and staggered schemes yield a convex landscape. Right: Critical load stage, where the monolithic scheme results in a non-convex landscape with multiple minima, while the staggered scheme ensures convexity in either the displacement or phase-field space.

It is evident that the J-integral couples K_{I}, K_{II}, and K_{III}. To decouple the mode I and mode II components, we introduce the phase angle ψ, defined as

$$\psi = \arctan\frac{K_{\mathrm{II}}}{K_{\mathrm{I}}} = \lim_{r\to 0,\theta=0} \arctan\frac{\tau_{12}}{\sigma_{22}}. \tag{47}$$

Once ψ is obtained, the individual SIFs are recovered as

$$\begin{aligned} K_{\mathrm{I}} &= \sqrt{EJ}\cos(\psi), \\ K_{\mathrm{II}} &= \sqrt{EJ}\sin(\psi). \end{aligned} \tag{48}$$

However, accurate evaluation of ψ requires information very close to the crack tip, where steep gradients make the computation challenging. Therefore, this method is generally more reliable for pure mode I or pure mode II cracks.

For mixed-mode cracks, the interaction integral method is more robust. We define

$$\begin{aligned} M^{(1)} &= \oint_{\Gamma} \left(w^{mix(1)}\delta_{1j} - \sigma_{\alpha\beta} u^{(1)}_{\alpha,1} - \sigma^{(1)}_{\alpha\beta} u_{\alpha,1} \right) n_j \, d\Gamma, \\ M^{(2)} &= \oint_{\Gamma} \left(w^{mix(2)}\delta_{1j} - \sigma_{\alpha\beta} u^{(2)}_{\alpha,1} - \sigma^{(2)}_{\alpha\beta} u_{\alpha,1} \right) n_j \, d\Gamma, \\ w^{mix(1)} &= \tfrac{1}{2}\left(\sigma_{\alpha\beta}\varepsilon^{(1)}_{\alpha\beta} + \sigma^{(1)}_{\alpha\beta}\varepsilon_{\alpha\beta}\right), \\ w^{mix(2)} &= \tfrac{1}{2}\left(\sigma_{\alpha\beta}\varepsilon^{(2)}_{\alpha\beta} + \sigma^{(2)}_{\alpha\beta}\varepsilon_{\alpha\beta}\right), \end{aligned} \tag{49}$$

where the superscripts (1) and (2) denote auxiliary (virtual) fields, constructed from the K-field representation in Eq. (7) (main file) by taking the $n = 1$ term. Once $M^{(1)}$ and $M^{(2)}$ are computed, the SIFs are obtained as

$$\begin{aligned} K_{\mathrm{I}} &= \frac{E}{2} M^{(1)}, \\ K_{\mathrm{II}} &= \frac{E}{2} M^{(2)}. \end{aligned} \tag{50}$$

The interaction integral is widely regarded as more reliable for mixed-mode fracture problems.

After obtaining K_{I} and K_{II}, the crack propagation angle can be estimated using the maximum hoop stress criterion:

$$\begin{aligned} \sigma_{\theta} &= \frac{1}{\sqrt{2\pi r}} \cos\frac{\theta}{2} \left[K_{\mathrm{I}}(1+\cos\theta) - 3K_{\mathrm{II}}\sin\theta \right], \\ \theta &= \arccos \frac{3K_{\mathrm{II}}^2 \pm \sqrt{K_{\mathrm{I}}^4 + 8K_{\mathrm{I}}^2 K_{\mathrm{II}}^2}}{K_{\mathrm{I}}^2 + 9K_{\mathrm{II}}^2}. \end{aligned} \tag{51}$$

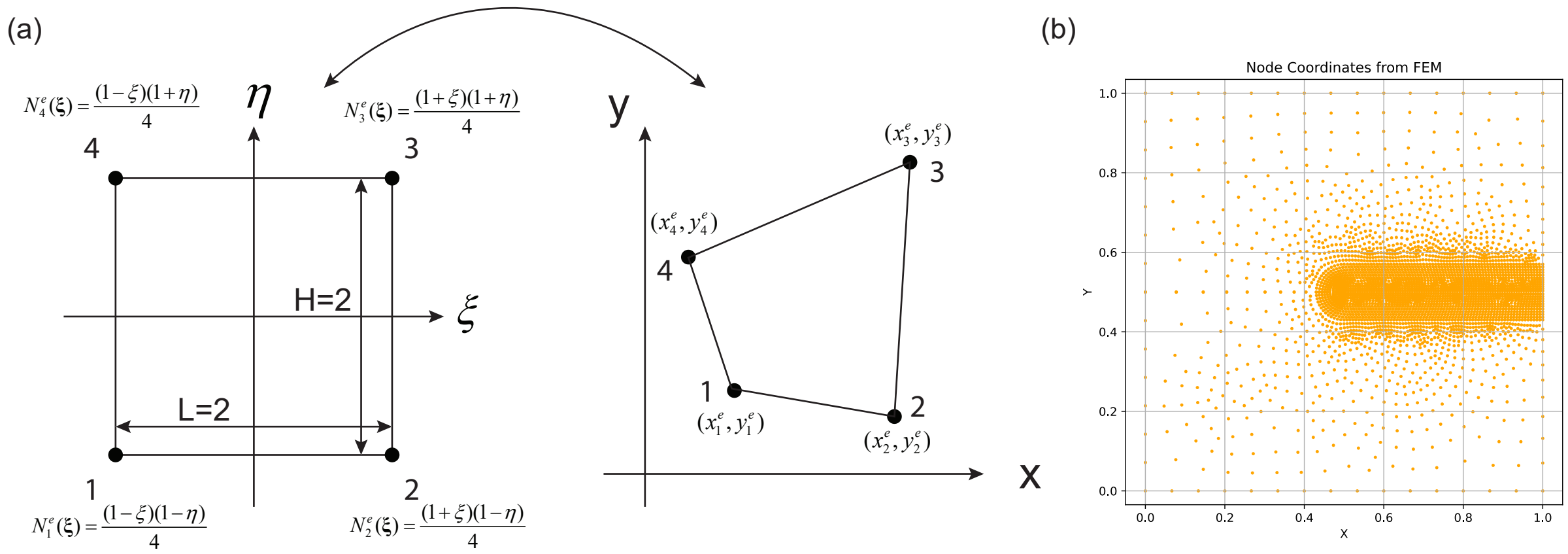

Supplementary Figure 22. (a) Mapping from the parent coordinates (ξ, η) of a standard square parent element to the physical coordinates (x, y) of the actual finite element. (b) Node distribution of the mesh in FEM.

Among the two candidate angles θ, the one corresponding to the maximum σ_θ is selected as the crack propagation direction.

3.9 UEL Details in FEM

Although the phase-field fracture model offers clear theoretical advantages, standard finite element software typically does not provide elements with an additional phase-field degree of freedom (DOF). Hence, a user-defined element (UEL) is required in order to couple the mechanical displacements with the phase field. In our implementation within ABAQUS, the UEL carries three DOFs per node in 2D: two displacement components (x, y) and the scalar phase field. The coupled system is solved in a staggered manner. Below we summarize the essential FEM details.

In the finite element discretization, the displacement field and the phase field are interpolated as

$$\begin{aligned}
\mathbf{u}(\mathbf{x}) &= \sum_{I=1}^{N_{\text{node}}} \mathbf{N}_I^{\mathrm{u}}(\mathbf{x})\,\mathbf{u}_I = \sum_{e=1}^{N_e}\sum_{I=1}^{m} \mathbf{N}_I^{\mathrm{u}e}(\mathbf{x})\,\mathbf{u}_I^e = \sum_{e=1}^{N_e} \mathbf{N}^{\mathrm{u}e}(\mathbf{x})\,\mathbf{u}^e, \\
\phi(\mathbf{x}) &= \sum_{I=1}^{N_{\text{node}}} N_I^{\phi}(\mathbf{x})\,\phi_I = \sum_{e=1}^{N_e}\sum_{I=1}^{m} N_I^{\phi e}(\mathbf{x})\,\phi_I^e = \sum_{e=1}^{N_e} \mathbf{N}^{\phi e}(\mathbf{x})\,\phi^e,
\end{aligned} \tag{52}$$

where $\mathbf{u}_I = \left[u_I^x \;\; u_I^y\right]^T$ is the nodal displacement and ϕ_I the nodal phase field. N_{node} and N_e are the total numbers of nodes and elements, respectively; $\mathbf{u}_I^e$ and ϕ_I^e are the element-level nodal DOFs. For 2D problems we use

$$\begin{aligned}
&\mathbf{N}_I^{\mathbf{u}}(\mathbf{x}) = \begin{bmatrix} N_I(\mathbf{x}) & \\ & N_I \end{bmatrix}; N_I^{\phi}(\mathbf{x}) = N_I \\
&\mathbf{N}^{\mathbf{u}e}(\mathbf{x}) = \begin{bmatrix} \mathbf{N}_I^{\mathrm{u}e}(\mathbf{x}) & \mathbf{N}_2^{\mathrm{u}e}(\mathbf{x}) & \cdots & \mathbf{N}_m^{\mathrm{u}e}(\mathbf{x}) \end{bmatrix}; \mathbf{N}^{\phi e}(\mathbf{x}) = \begin{bmatrix} N_I^{\phi e}(\mathbf{x}) & N_2^{\phi e}(\mathbf{x}) & \cdots & N_m^{\phi e}(\mathbf{x}) \end{bmatrix}. \\
&\mathbf{u}^e = \begin{bmatrix} \mathbf{u}_1^e & \mathbf{u}_2^e & \cdots & \mathbf{u}_m^e \end{bmatrix}^T; \phi^e = \begin{bmatrix} \phi_1^e & \phi_2^e & \cdots & \phi_m^e \end{bmatrix}^T
\end{aligned} \tag{53}$$

Here m denotes the number of nodes per element; we adopt 4-node quadrilateral elements, so $m = 4$. Unless otherwise stated, vectors are written as column vectors.

For the 4-node quadrilateral element, the shape functions in the parent (reference) coordinates $\xi = (\xi, \eta) \in [-1, 1] \times [-1, 1]$ are

$$\begin{aligned}
N_1^e(\xi) &= \tfrac{(1-\xi)(1-\eta)}{4}, \qquad N_2^e(\xi) = \tfrac{(1+\xi)(1-\eta)}{4}, \\
N_3^e(\xi) &= \tfrac{(1+\xi)(1+\eta)}{4}, \qquad N_4^e(\xi) = \tfrac{(1-\xi)(1+\eta)}{4}.
\end{aligned} \tag{54}$$

The mapping from the parent element to the physical element is standard in FEM (see Supplementary Fig. 22a and any FEM book[25]).

Taking spatial derivatives for the phase field, we obtain

$$\begin{aligned}
&\frac{\partial \phi(\mathbf{x})}{\partial \mathbf{x}} = \begin{bmatrix} \partial\phi/\partial x \\ \partial\phi/\partial y \end{bmatrix} = \sum_{e=1}^{N_e} \mathbf{B}^{\phi e}(\mathbf{x})\,\phi^e, \\
&\mathbf{B}^{\phi e}(\mathbf{x}) = \begin{bmatrix} \partial N_1^e/\partial x & \partial N_2^e/\partial x & \partial N_3^e/\partial x & \partial N_4^e/\partial x \\ \partial N_1^e/\partial y & \partial N_2^e/\partial y & \partial N_3^e/\partial y & \partial N_4^e/\partial y \end{bmatrix}, \qquad \phi^e = \begin{bmatrix} \phi_1^e\ \phi_2^e\ \phi_3^e\ \phi_4^e \end{bmatrix}^T.
\end{aligned} \tag{55}$$

Since N_I^e is defined in the parent coordinates, chain rule gives

$$\begin{aligned}
\frac{\partial N_I^e(\mathbf{x})}{\partial x} &= \frac{\partial N_I^e(\xi)}{\partial \xi}\frac{\partial \xi}{\partial x} = \begin{bmatrix} \partial\xi/\partial x & \partial\eta/\partial x \end{bmatrix} \begin{bmatrix} \partial N_I^e/\partial \xi \\ \partial N_I^e/\partial \eta \end{bmatrix}, \\
\frac{\partial N_I^e(\mathbf{x})}{\partial y} &= \frac{\partial N_I^e(\xi)}{\partial \xi}\frac{\partial \xi}{\partial y} = \begin{bmatrix} \partial\xi/\partial y & \partial\eta/\partial y \end{bmatrix} \begin{bmatrix} \partial N_I^e/\partial \xi \\ \partial N_I^e/\partial \eta \end{bmatrix}.
\end{aligned} \tag{56}$$

Hence

$$\mathbf{B}^e(\mathbf{x}) = (\mathbf{J}^e)^{-1}\,\mathbf{N}_\xi^e, \tag{57}$$

$$(\mathbf{J}^e)^{-1} = \begin{bmatrix} \partial\xi/\partial x & \partial\eta/\partial x \\ \partial\xi/\partial y & \partial\eta/\partial y \end{bmatrix}, \qquad \mathbf{J}^e = \begin{bmatrix} \partial x/\partial\xi & \partial y/\partial\xi \\ \partial x/\partial\eta & \partial y/\partial\eta \end{bmatrix}, \tag{58}$$

$$\mathbf{N}_\xi^e = \begin{bmatrix} \partial N_1^e/\partial\xi & \partial N_2^e/\partial\xi & \partial N_3^e/\partial\xi & \partial N_4^e/\partial\xi \\ \partial N_1^e/\partial\eta & \partial N_2^e/\partial\eta & \partial N_3^e/\partial\eta & \partial N_4^e/\partial\eta \end{bmatrix} = \begin{bmatrix} -\frac{(1-\eta)}{4} & \frac{(1-\eta)}{4} & \frac{(1+\eta)}{4} & -\frac{(1+\eta)}{4} \\ -\frac{(1-\xi)}{4} & -\frac{(1+\xi)}{4} & \frac{(1+\xi)}{4} & \frac{(1-\xi)}{4} \end{bmatrix}. \tag{59}$$

By interpolating the physical coordinates with the same shape functions, the Jacobian can be written as

$$\mathbf{J}^e = \begin{bmatrix} \dfrac{\partial\left[\sum_{I=1}^m N_I^e(\xi)\,x_I^e\right]}{\partial\xi} & \dfrac{\partial\left[\sum_{I=1}^m N_I^e(\xi)\,y_I^e\right]}{\partial\xi} \\ \dfrac{\partial\left[\sum_{I=1}^m N_I^e(\xi)\,x_I^e\right]}{\partial\eta} & \dfrac{\partial\left[\sum_{I=1}^m N_I^e(\xi)\,y_I^e\right]}{\partial\eta} \end{bmatrix} = \mathbf{N}_\xi^e\,\mathbf{X}^e, \tag{60}$$

$$\mathbf{X}^e = \begin{bmatrix} x_1^e & x_2^e & x_3^e & x_4^e \\ y_1^e & y_2^e & y_3^e & y_4^e \end{bmatrix}^T, \tag{61}$$

where (x_I^e, y_I^e) are the physical coordinates of the element nodes.

Substituting Supplementary Eq. (60) into Supplementary Eq. (57), and then using $\mathbf{B}^{\phi e}$ in Supplementary Eq. (55), we obtain

$$\frac{\partial\phi(\mathbf{x})}{\partial\mathbf{x}} = \sum_{e=1}^{N_e} \mathbf{B}^{\phi e}(\mathbf{x})\,\phi^e = \sum_{e=1}^{N_e} \left(\mathbf{N}_\xi^e\mathbf{X}^e\right)^{-1}\mathbf{N}_\xi^e\,\phi^e. \tag{62}$$

The same procedure applies to the displacement gradient.

Inserting Supplementary Eq. (62) and Supplementary Eq. (52) into the first variation Supplementary Eq. (38) yields

$$\begin{aligned}
\delta\Pi =& \int_\Omega \Big[w'(\phi)\,\delta\phi\,\Psi^+(\boldsymbol{\varepsilon}) + w(\phi)\,\sigma_{ij}^+\,\delta\varepsilon_{ij} + \sigma_{ij}^-\,\delta\varepsilon_{ij}\Big]\,dV + \int_\Omega \Big\{\frac{G_c}{l_0}\big[\phi\,\delta\phi + l_0^2(\nabla\phi)\cdot(\nabla\delta\phi)\big] + w'(\phi)\,\delta\phi\,H(\Psi^+)\Big\}\,dV \\
&- \int_\Omega \mathbf{f}\cdot\delta\mathbf{u}\,dV - \int_{\Gamma^t} \bar{\mathbf{t}}\cdot\delta\mathbf{u}\,dS \\
=& \sum_{e=1}^{N_e}\Bigg\{\int_{\Omega^e}\Big[w'(\phi)\,\mathbf{N}^e\delta\phi^e\,\Psi^+(\boldsymbol{\varepsilon}) + \big(\mathbf{D}\langle\mathbf{B}^e\mathbf{u}^e\rangle_+\big)^T\mathbf{B}^e\delta\mathbf{u}^e\,w(\phi) + \big(\mathbf{D}\langle\mathbf{B}^e\mathbf{u}^e\rangle_-\big)^T\mathbf{B}^e\delta\mathbf{u}^e - \mathbf{f}\cdot\mathbf{N}^e\delta\mathbf{u}^e\Big]\,dV \\
&+ \int_{\Omega^e}\Big\{\frac{G_c}{l_0}\big[(\mathbf{N}^e\phi^e)^T\mathbf{N}^e\delta\phi^e + l_0^2(\mathbf{B}^e\phi^e)^T\mathbf{B}^e\delta\phi^e\big] + w'(\phi)\,\mathbf{N}^e\delta\phi^e H(\Psi^+)\Big\}\,dV\Bigg\} - \int_{\Gamma^{te}} \bar{\mathbf{t}}\cdot\mathbf{N}^e\delta\mathbf{u}^e\,dS \\
=& \sum_{e=1}^{N_e}\Bigg\{\int_{\Omega^e}\Big[w'(\phi)\,\mathbf{N}^e\,\Psi^+(\boldsymbol{\varepsilon}) + \frac{G_c}{l_0}\big(\mathbf{N}^{eT}\mathbf{N}^e\phi^e + l_0^2\mathbf{B}^{eT}\mathbf{B}^e\phi^e\big) + w'(\phi)\,\mathbf{N}^e H(\Psi^+)\Big]\,dV\ \delta\phi^e \\
&+ \Big[\int_{\Omega^e}\big(w(\phi)\mathbf{D}\langle\mathbf{B}^e\mathbf{u}^e\rangle_+^T\mathbf{B}^e + \mathbf{D}\langle\mathbf{B}^e\mathbf{u}^e\rangle_-^T\mathbf{B}^e\big)\,dV - \int_{\Omega^e}\mathbf{f}\cdot\mathbf{N}^e\,dV - \int_{\Gamma^{te}}\bar{\mathbf{t}}\cdot\mathbf{N}^e\,dS\Big]\,\delta\mathbf{u}^e\Bigg\}.
\end{aligned} \tag{63}$$

The resulting nonlinear residual equations at the element level are

$$
\begin{aligned}
\mathbf{R}_{\mathbf{u}^e} &= \int_{\Omega^e} \left[w(\phi)\, \mathbf{B}^{eT} \mathbf{D} \langle \mathbf{B}^e \mathbf{u}^e \rangle_+ + \mathbf{B}^{eT} \mathbf{D} \langle \mathbf{B}^e \mathbf{u}^e \rangle_- \right] dV - \int_{\Omega^e} \mathbf{N}^{eT} \mathbf{f}\, dV - \int_{\Gamma^{\mathbf{t}e}} \mathbf{N}^{eT} \bar{\mathbf{t}}\, dS = \mathbf{0}, \\
\mathbf{R}_{\phi^e} &= \int_{\Omega^e} \left[w'(\phi)\, \mathbf{N}^{eT} \Psi^+(\boldsymbol{\varepsilon}) + \frac{G_c}{l_0} \left(\mathbf{N}^{eT} \mathbf{N}^e \phi^e + l_0^2 \mathbf{B}^{eT} \mathbf{B}^e \phi^e \right) + w'(\phi)\, \mathbf{N}^{eT} H(\Psi^+) \right] dV = \mathbf{0}.
\end{aligned}
\tag{64}
$$

We linearize the system via Newton's method. The element tangent matrix reads

$$
\mathbf{K}^e = \begin{bmatrix} \mathbf{K}^e_{\mathbf{uu}} & \mathbf{K}^e_{\mathbf{u}\phi} \\ \mathbf{K}^e_{\phi\mathbf{u}} & \mathbf{K}^e_{\phi\phi} \end{bmatrix} = \begin{bmatrix} \partial \mathbf{R}_{\mathbf{u}^e} / \partial \mathbf{u}^e & \partial \mathbf{R}_{\mathbf{u}^e} / \partial \phi^e \\ \partial \mathbf{R}_{\phi^e} / \partial \mathbf{u}^e & \partial \mathbf{R}_{\phi^e} / \partial \phi^e \end{bmatrix}, \qquad \frac{\partial \mathbf{R}_{\phi^e}}{\partial \mathbf{u}^e} = \left(\frac{\partial \mathbf{R}_{\mathbf{u}^e}}{\partial \phi^e} \right)^T = \int_{\Omega^e} w'(\phi)\, \mathbf{B}^{eT} \mathbf{D} \langle \mathbf{B}^e \mathbf{u}^e \rangle_+ \mathbf{N}^e\, dV, \tag{65}
$$

with

$$
\frac{\partial \mathbf{R}_{\mathbf{u}^e}}{\partial \mathbf{u}^e} = \frac{\partial}{\partial \mathbf{u}^e} \int_{\Omega^e} \left[w(\phi)\, \mathbf{B}^{eT} \mathbf{D} \langle \mathbf{B}^e \mathbf{u}^e \rangle_+ + \mathbf{B}^{eT} \mathbf{D} \langle \mathbf{B}^e \mathbf{u}^e \rangle_- \right] dV, \tag{66}
$$

$$
\frac{\partial \mathbf{R}_{\phi^e}}{\partial \phi^e} = \int_{\Omega^e} \left[w''(\phi)\, \mathbf{N}^{eT} \mathbf{N}^e \Psi^+(\boldsymbol{\varepsilon}) + \frac{G_c}{l_0} \left(\mathbf{N}^{eT} \mathbf{N}^e + l_0^2 \mathbf{B}^{eT} \mathbf{B}^e \right) + w''(\phi)\, \mathbf{N}^{eT} \mathbf{N}^e H(\Psi^+) \right] dV. \tag{67}
$$

Due to the inherent non-convexity of the fracture energy (see the analyses in Supplementary Note 3.7), the global tangent may be indefinite, which affects numerical stability and convergence. A common remedy is to drop the off-diagonal coupling blocks $\mathbf{K}^e_{\mathbf{u}\phi}$ and $\mathbf{K}^e_{\phi\mathbf{u}}$, and to employ a staggered solution: solve alternately for the displacement and phase-field subproblems.

In our UEL implementation, we exploit the block structure and adopt a staggered iterative scheme. To accurately resolve the diffusive crack, the element size h must be chosen sufficiently small relative to the phase-field length scale l_0. For the single-edge notched square specimen with a mode-I crack, a total of 3259 user elements are used. The bottom edge is fully clamped; the crack faces are traction-free. A uniform tensile displacement is prescribed on the top boundary. To extract the load-displacement curve, all y-DOFs on the top edge are coupled to a reference point, where the displacement is applied and the reaction force is recorded. The mesh (with local refinement near the crack tip) is illustrated in Supplementary Fig. 22b.

Supplementary Note 4 Supplementary Figures

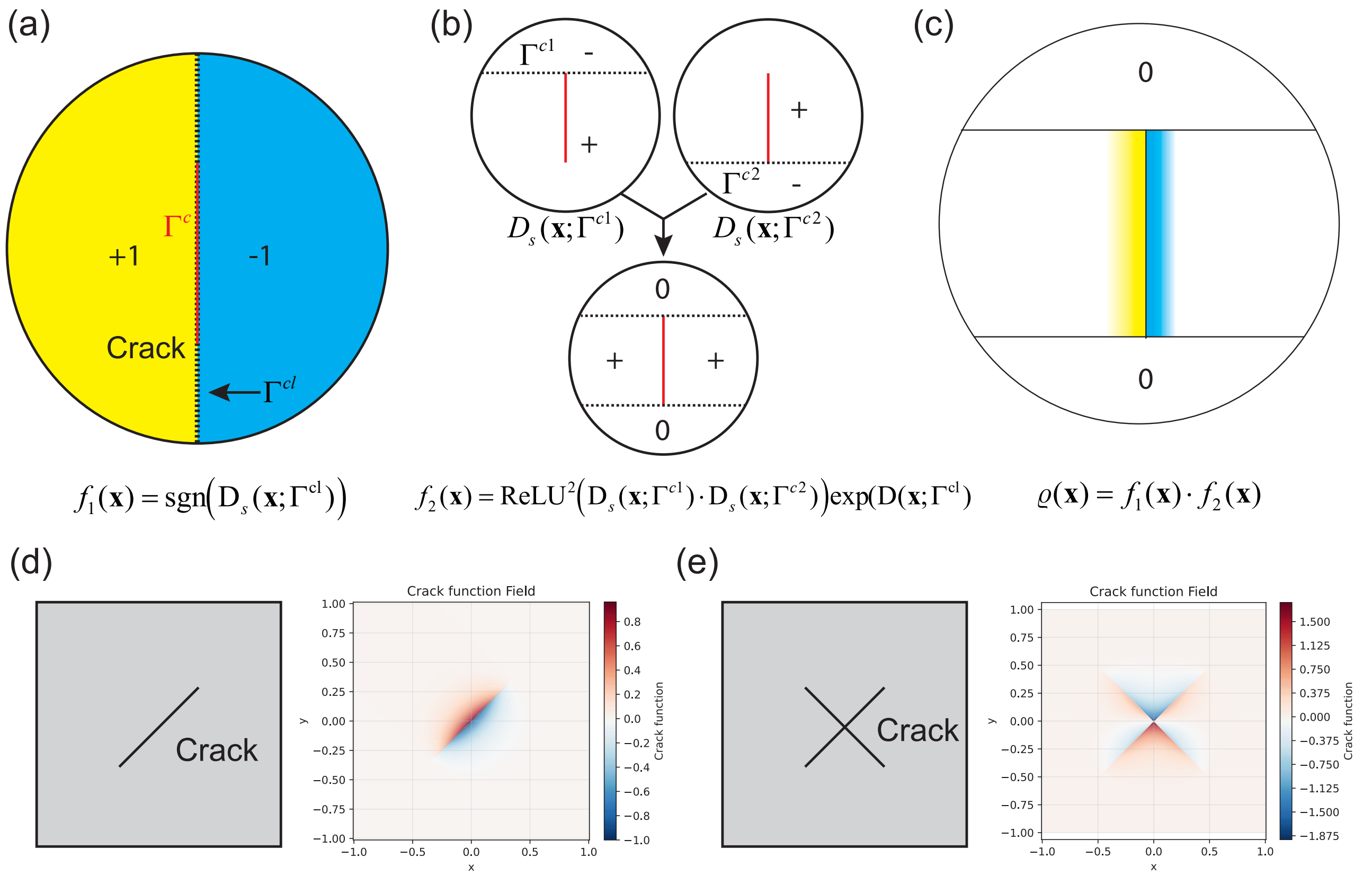

Supplementary Figure 23. Illustration of the crack function: (a) signed distance function calculation for the crack followed by the sgn operator; (b) signed distance functions calculated for the two endpoints Γ^{c1} and Γ^{c2}, processed by ReLU^2 and multiplied by a decay function; (c) schematic of the crack function; (d) crack function for an inclined crack; (e) crack function for intersecting cracks.

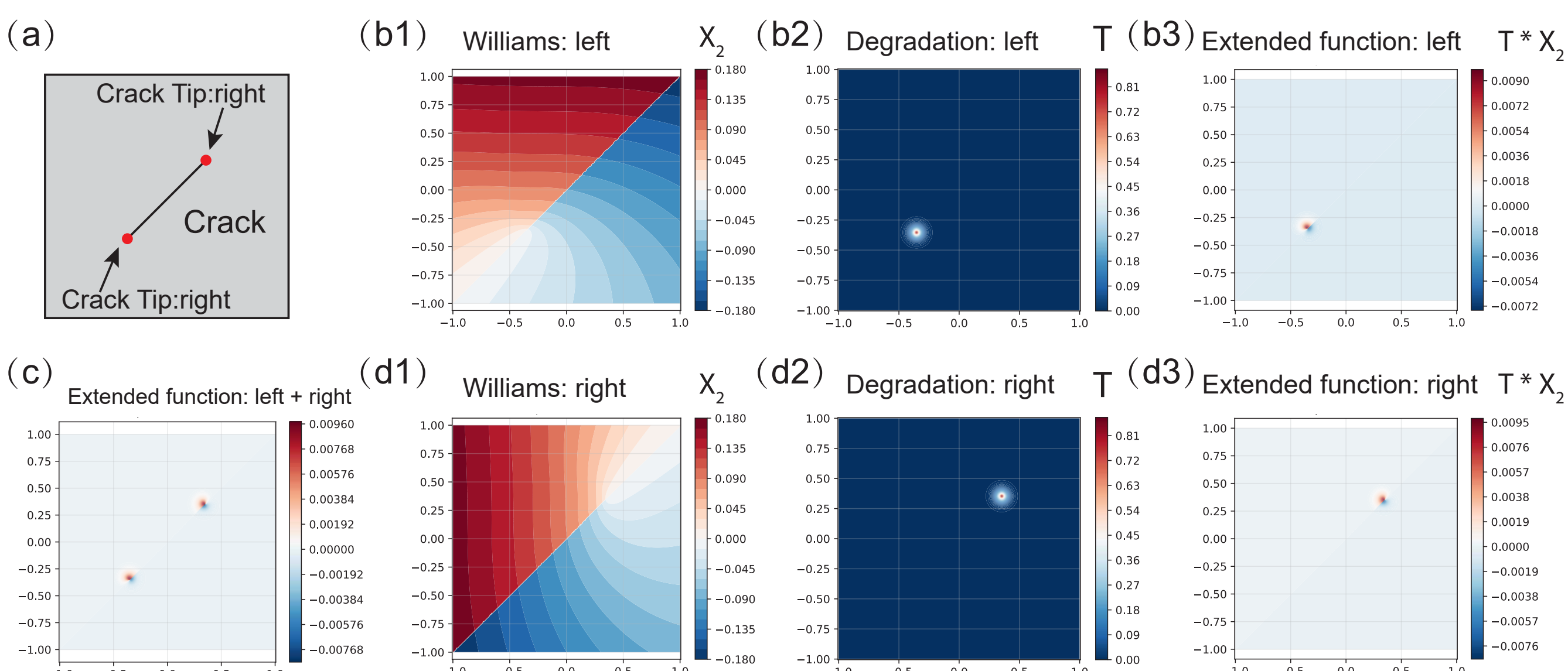

Supplementary Figure 24. Illustration of the extended function: (a) both crack tips of an inclined crack require extended functions; (b1–3) Williams series solution X_2, decay function T, and extended function $T \cdot X_2$ at the left crack tip; (c) combined extended functions at both crack tips; (d1–3) Williams series solution X_2, decay function T, and extended function $T \cdot X_2$ at the right crack tip.

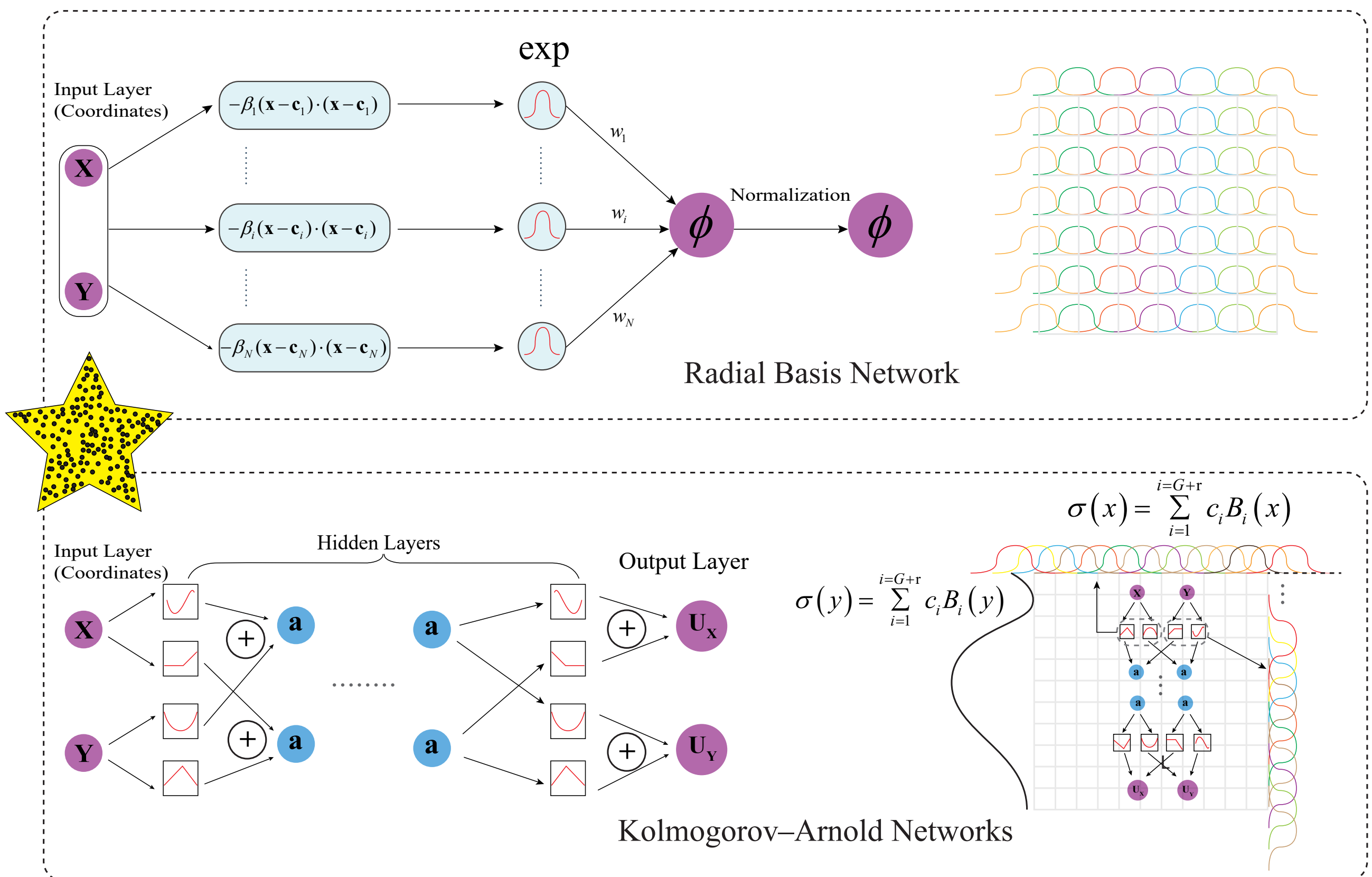

Supplementary Figure 25. Network architectures of the Radial Basis Function Network and the Kolmogorov–Arnold Network. (a) The Radial Basis Function Network maps the input coordinates through radial basis functions before producing the output. (b) The Kolmogorov–Arnold Network represents the input–output relationship through learnable activation functions.

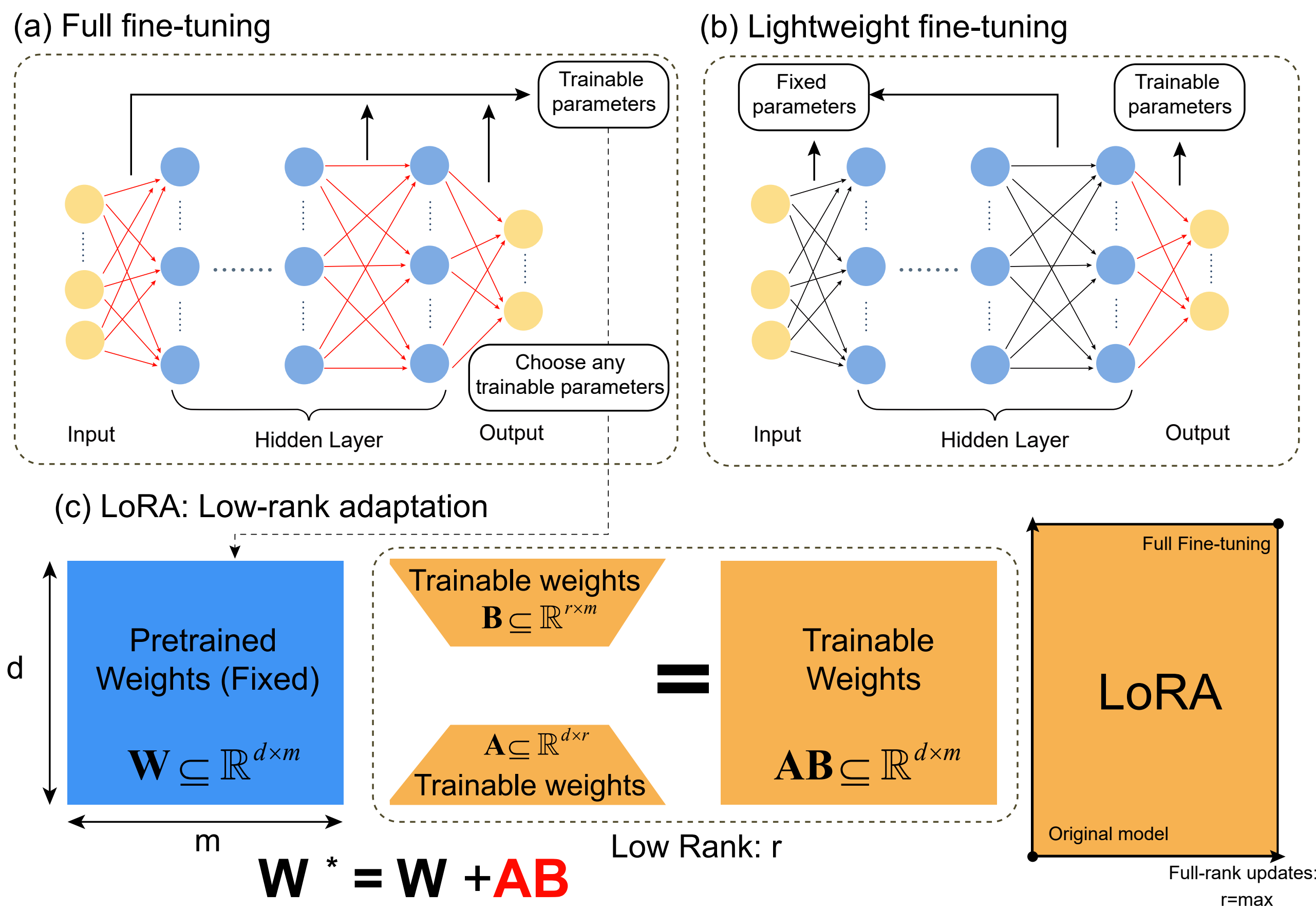

Supplementary Figure 26. Three common parameter-based transfer learning strategies: (a) Full fine-tuning: all parameters are updated (red arrows indicate trainable parameters). (b) Lightweight fine-tuning: only a subset of parameters is updated (red arrows). (c) LoRA: the blue matrix **W** denotes fixed pretrained weights, while the yellow matrices **A** and **B** represent trainable low-rank factors. During inference, the adapted weights are given by $\mathbf{W}^* = \mathbf{W} + \mathbf{AB}$.

Supplementary References

1. Erdogan, F. & Sih, G. On the crack extension in plates under plane loading and transverse shear. *Journal of basic engineering* **85**, 519–525 (1963).

2. Hussain, M., Pu, S. & Underwood, J. Strain energy release rate for a crack under combined mode i and mode ii. In *Fracture analysis: Proceedings of the 1973 national symposium on fracture mechanics, part II*, vol. 560, 2 (ASTM International, 1974).

3. Sih, G. C. Strain-energy-density factor applied to mixed mode crack problems. *International Journal of fracture* **10**, 305–321 (1974).

4. Bourdin, B., Francfort, G. A. & Marigo, J.-J. Numerical experiments in revisited brittle fracture. *Journal of the Mechanics and Physics of Solids* **48**, 797–826 (2000).

5. Francfort, G. A. & Marigo, J.-J. Revisiting brittle fracture as an energy minimization problem. *Journal of the Mechanics and Physics of Solids* **46**, 1319–1342 (1998).

6. Miehe, C., Hofacker, M. & Welschinger, F. A phase field model for rate-independent crack propagation: Robust algorithmic implementation based on operator splits. *Computer Methods in Applied Mechanics and Engineering* **199**, 2765–2778 (2010).

7. Amor, H., Marigo, J.-J. & Maurini, C. Regularized formulation of the variational brittle fracture with unilateral contact: Numerical experiments. *Journal of the Mechanics and Physics of Solids* **57**, 1209–1229 (2009).

8. Gerasimov, T. & De Lorenzis, L. On penalization in variational phase-field models of brittle fracture. *Computer Methods in Applied Mechanics and Engineering* **354**, 990–1026 (2019).

9. Samaniego, E. *et al.* An energy approach to the solution of partial differential equations in computational mechanics via machine learning: Concepts, implementation and applications. *Computer Methods in Applied Mechanics and Engineering* **362**, 112790 (2020).

10. Zhao, L. & Shao, Q. Denns: Discontinuity-embedded neural networks for fracture mechanics. *Computer Methods in Applied Mechanics and Engineering* **446**, 118184 (2025).

11. Goswami, S., Anitescu, C., Chakraborty, S. & Rabczuk, T. Transfer learning enhanced physics informed neural network for phase-field modeling of fracture. *Theoretical and Applied Fracture Mechanics* **106**, 102447 (2020).

12. Wang, Y., Sun, J., Li, W., Lu, Z. & Liu, Y. Cenn: Conservative energy method based on neural networks with subdomains for solving variational problems involving heterogeneous and complex geometries. *Computer Methods in Applied Mechanics and Engineering* **400**, 115491 (2022).

13. Tada, H., Paris, P. C. & Irwin, G. R. The stress analysis of cracks. *Handbook, Del Research Corporation* **34** (1973).

14. Goswami, S., Anitescu, C. & Rabczuk, T. Adaptive fourth-order phase field analysis for brittle fracture. *Computer Methods in Applied Mechanics and Engineering* **361**, 112808 (2020).

15. Manav, M., Molinaro, R., Mishra, S. & De Lorenzis, L. Phase-field modeling of fracture with physics-informed deep learning. *Computer Methods in Applied Mechanics and Engineering* **429**, 117104 (2024).

16. Moës, N., Dolbow, J. & Belytschko, T. A finite element method for crack growth without remeshing. *International journal for numerical methods in engineering* **46**, 131–150 (1999).

17. Williams, M. L. On the stress distribution at the base of a stationary crack. *Journal of Applied Mechanics* (1957).

18. Wang, Y. *et al.* Kolmogorov–arnold-informed neural network: A physics-informed deep learning framework for solving forward and inverse problems based on kolmogorov–arnold networks. *Computer Methods in Applied Mechanics and Engineering* **433**, 117518 (2025).

19. Hu, E. J. *et al.* Lora: Low-rank adaptation of large language models. *arXiv preprint arXiv:2106.09685* (2021).

20. Wang, Y. *et al.* Transfer learning in physics-informed neurals networks: Full fine-tuning, lightweight fine-tuning, and low-rank adaptation. *International Journal of Mechanical System Dynamics* (2025).

21. Hornik, K., Stinchcombe, M. & White, H. Multilayer feedforward networks are universal approximators. *Neural networks* **2**, 359–366 (1989).

22. Liu, Z. *et al.* Kan: Kolmogorov-arnold networks. *arXiv preprint arXiv:2404.19756* (2024).

23. He, K., Zhang, X., Ren, S. & Sun, J. Deep residual learning for image recognition. In *Proceedings of the IEEE conference on computer vision and pattern recognition*, 770–778 (2016).

24. Shukla, K., Toscano, J. D., Wang, Z., Zou, Z. & Karniadakis, G. E. A comprehensive and fair comparison between mlp and kan representations for differential equations and operator networks. *Computer Methods in Applied Mechanics and Engineering* **431**, 117290 (2024).

25. Zienkiewicz, O. C., Taylor, R. L. & Zhu, J. Z. *The finite element method: its basis and fundamentals* (Elsevier, 2005).
