# Supplementary material for: Towards unified AI-driven fracture mechanics: the extended deep energy method (XDEM)
Source: Nat Commun. 2026 Aug 15;17:8492. doi: 10.1038/s41467-026-76748-1 (PMC13484471; doi:10.1038/s41467-026-76748-1)
Supplement: Supplementary file 2 [file 41467_2026_76748_MOESM2_ESM.pdf]

nature portfolio

Peer Review File

Towards Unified AI-Driven Fracture Mechanics: The Extended Deep Energy Method (XDEM)

Corresponding Author: Professor Yinghua Liu

This file contains all reviewer reports in order by version, followed by all author rebuttals in order by version.

Version 0:

Reviewer comments:

Reviewer #1

(Remarks to the Author)
In this paper, the authors extend the Deep Energy Method, a physics-informed deep learning framework, to accurately handle both discrete cracks and phase-field models within a single approach. The proposed XDEM overcomes key limitations of previous methods by requiring fewer, uniformly distributed collocation points while capturing crack discontinuities and tip behavior more effectively. Benchmark tests show that XDEM delivers higher accuracy and efficiency in predicting crack growth and stress intensity compared to standard DEM.

Comments and Questions:
The major contribution of this work is the development of a novel physics-informed deep learning framework for fracture mechanics problems. The key idea is to enrich neural network architectures using crack and extended functions, similar to the extended finite element method (XFEM). The manuscript is well written and presents a wide range of results supporting its conclusions and claims. It is commendable that the authors provided a detailed description of the method in Section S3 Extended Discussion on XDEM. The reviewer suggests minor revisions to address the following concerns.

The reviewer would like to kindly ask the authors the following questions:
1. In the Results section, it would improve readability to clarify which problems are solved using XDEM-C and XDEM-D. Also, the Method section typically precedes the Results section.
2. In Fig. 3, a comparison with reference solutions would strengthen the claim of accuracy (similar to Fig. 2 if FEM reference solutions exist). In Fig. 4, the caption “(c)” is missing. In the caption of Fig. 5, there appears to be a repetition of the Young’s moduli E1 and E2.
3. On page 5, it is stated that “XDEM naturally accommodates such effects through its energy-based formulation and extended neural representation.” Strength-based formulations without a variational form may not be supported by XDEM. If that is the case, please acknowledge this limitation.
4. In the paper, it is stated that “traction-free conditions are imposed along the crack surface \Gamma_c.” Please elaborate on the form of the additional conditions that may be needed in the problem definition (energies and strong forms).
5. In Table S1, the functions w, g, X_1, X_2 are not presented.
6. Please comment on the reason for using two different types of neural network architectures, namely KAN and RBF, for the displacement and phase-field, respectively.
7. In Fig. 12b, XDEM-C indeed accurately captures the failure displacement, but it appears to be less accurate for the peak force.

(Remarks on code availability)

Reviewer #2

(Remarks to the Author)
The paper presents XDEM, essentially an extended DEM for fracture mechanics. XDEM is in spirit like a classical XFEM enrichment, i.e., it explicitly introduces a crack functon(like a Heaviside in XFEM) and a a Williams-series-based extended functon(analogous to crack-tip asymptotic enrichment). The paper is well written. The computational examples are

extensive. While the contribution is interesting and the presentation is generally clear, I have a number of major and minor comments that, in my opinion, need to be addressed to strengthen the manuscript.

Major comments:
- The quality and size of several figures are insufficient for publication. In particular, figures such as Fig. 2 and Fig. 3 have axis labels, tick marks, legends, and titles that are barely readable at normal viewing scale. This significantly hampers the reader's ability to assess the results. All figures should be enlarged and reformatted with substantially larger fonts and improved resolution to ensure clarity in both print and digital formats.

- In Section S3.3, sequential LoRA transfer is said to potentially induce non-physical fracture patterns (linked to Section S4.1), which are attributed to integration inaccuracies and zero-energy modes. Could the authors clarify how exactly LoRA interacts with this mechanism?

Additionally, do the reported wall-times and efficiency comparisons correspond to fully sequential LoRA transfer between load increments, or to transfer always initialized from the first load step? This distinction seems important for assessing the robustness and actual computational savings of the proposed strategy. Especially since the code is not online yet.

Minor comments:
- p.3 "(modes I, II and III).."
- Fig.S23 says "Theretical Minimum"
- there is a superscript in reference 2: Hussain, M., Pu, S. & Underwood"1" , J. Strain energy release rate e for a crack under combined mode i and mode ii.
In Fracture analysis: Proceedings of the 1973 national symposium on fracture mechanics, part II, vol. 560, 2 (ASTM International, 1974).

(Remarks on code availability)
The code is not available.

Version 1:

Reviewer comments:

Reviewer #1

(Remarks to the Author)

(Remarks on code availability)

Open Access This Peer Review File is licensed under a Creative Commons Attribution 4.0 International License, which

permits use, sharing, adaptation, distribution and reproduction in any medium or format, as long as you give appropriate credit to the original author(s) and the source, provide a link to the Creative Commons license, and indicate if changes were made.
In cases where reviewers are anonymous, credit should be given to 'Anonymous Referee' and the source.
The images or other third party material in this Peer Review File are included in the article's Creative Commons license, unless indicated otherwise in a credit line to the material. If material is not included in the article's Creative Commons license and your intended use is not permitted by statutory regulation or exceeds the permitted use, you will need to obtain permission directly from the copyright holder.
To view a copy of this license, visit https://creativecommons.org/licenses/by/4.0/

The Response to Reviewers for Revision

Nature Communications,

Manuscript ID: NCOMMS-25-95353A

Towards Unified AI-Driven Fracture Mechanics: The Extended Deep Energy Method (XDEM)

Yizheng Wang, Yuzhou Lin, Somdatta Goswami, Luyang Zhao, Huadong Zhang, Jinshuai Bai, Cosmin Anitescu, Mohammad Sadegh Eshaghi, Xiaoying Zhuang, Timon Rabczuk, Yinghua Liu

Corresponding Author: Yinghua Liu

yhliu@mail.tsinghua.edu.cn

To Editors and Reviewers

We would like to thank the editor for giving us the opportunity to revise our manuscript. We also want to thank the reviewers for their insightful questions and suggestions. We have carefully considered all the comments and revised our manuscript accordingly. In this revision, we believe that we have fully addressed the concerns of the reviewers. The quality and clarity of our manuscript have been further improved as a result.

We have made our code open-source on the github, and it is available at the following link: `https://github.com/yizheng-wang/XDEM`

Those changes are highlighted within the manuscript. Please see below, for a point-by-point response to the reviewers' comments and concerns.

All page numbers refer to the revised manuscript file with tracked changes.

Reviewer #1

The Reviewer's Comment

In this paper, the authors extend the Deep Energy Method, a physics-informed deep learning framework, to accurately handle both discrete cracks and phase-field models within a single approach. The proposed XDEM overcomes key limitations of previous methods by requiring fewer, uniformly distributed collocation points while capturing crack discontinuities and tip behavior more effectively. Benchmark tests show that XDEM delivers higher accuracy and efficiency in predicting crack growth and stress intensity compared to standard DEM.
Comments and Questions: The major contribution of this work is the development of a novel physics-informed deep learning framework for fracture mechanics problems. The key idea is to enrich neural network architectures using crack and extended functions, similar to the extended finite element method (XFEM). The manuscript is well written and presents a wide range of results supporting its conclusions and claims. It is commendable that the authors provided a detailed description of the method in Section S3 Extended Discussion on XDEM. The reviewer suggests minor revisions to address the following concerns.

Response:

Thank you for your recognition of this work. Your response was very professional. We sincerely appreciate your insightful feedback, which has significantly improved the quality of our work. We have revised the manuscript based on your comments.

The Reviewer's Comment #1

1. In the Results section, it would improve readability to clarify which problems are solved using XDEM-C and XDEM-D. Also, the Method section typically precedes the Results section.

Response:

Thanks for your comments. We have added clarification in the Results section regarding which problems are solved using XDEM-C or XDEM-D. As the Nature series papers typically require the Results section to precede the Method section, we made this clarification at the beginning of the Results section, as shown below:

"...

Results

This section presents the performance of the proposed Extended Deep Energy Method (XDEM) in modeling fracture evolution under varying loading and boundary conditions. XDEM is presented in two forms: discrete (XDEM-D) and continuous (XDEM-C). The results discussed in the main text pertain to XDEM-D, while results for XDEM-C are provided in the Supplementary Section. The results are organized into two parts: (i) prediction of stress intensity factors (SIFs), which quantify the near-tip stress fields, and (ii) simulation of crack path propagation under different fracture modes (modes I, II and III). ..."

The Reviewer's Comment #2

2. In Fig. 3, a comparison with reference solutions would strengthen the claim of accuracy (similar to Fig. 2 if FEM reference solutions exist). In Fig. 4, the caption (c) is missing. In the caption of Fig. 5, there appears to be a repetition of the Youngs moduli E1 and E2.

Response:

Thanks for your comment. We have added the FEM reference solution for Fig. 2. The FEM reference solution is obtained using COMSOL

"··· Finally, to further illustrate the generality of XDEM, Fig. R1 presents results for intersecting cracks. XDEM accurately reproduces displacement discontinuities along multiple crack interfaces in close agreement with the reference solution [1]. These results highlight the robustness of XDEM in representing complex crack topologies without requiring adaptive meshing or problem-specific refinement. ···"

Response:

Regarding Fig. 4, it has been corrected. As for Fig. 5, there is no repetition; it actually represents two different cases, soft and hard, with 4 values of E1 and E2.

The Reviewer's Comment #3

3. On page 5, it is stated that XDEM naturally accommodates such effects through its energy-based formulation and extended neural representation. Strength-based formulations without a variational form may not be supported by XDEM. If that is the case, please acknowledge this limitation.

Response:

Thank you for your valuable suggestion. The statement: "It is noteworthy that, as discussed by [2], classical variational phase-field formulations are generally unable to capture crack nucleation due to the absence of an explicit representation of material strength as an independent macroscopic parameter. In contrast, XDEM naturally accommodates such effects through its energy-based formulation and extended neural representation." may require further exploration. We believe your point might be correct. However, this idea diverges somewhat from the main theme of our paper, so we have decided to remove it.

The Reviewer's Comment #4

4. In the paper, it is stated that traction-free conditions are imposed along the crack surface Γ_c. Please elaborate on the form of the additional conditions that may be needed in the problem definition (energies and strong forms).

Response:

Thank you for your valuable suggestion. We have elaborated on the form of the additional conditions (i.e., non-traction-free crack faces) in Supplementary Section S1.1, as follows:

"···

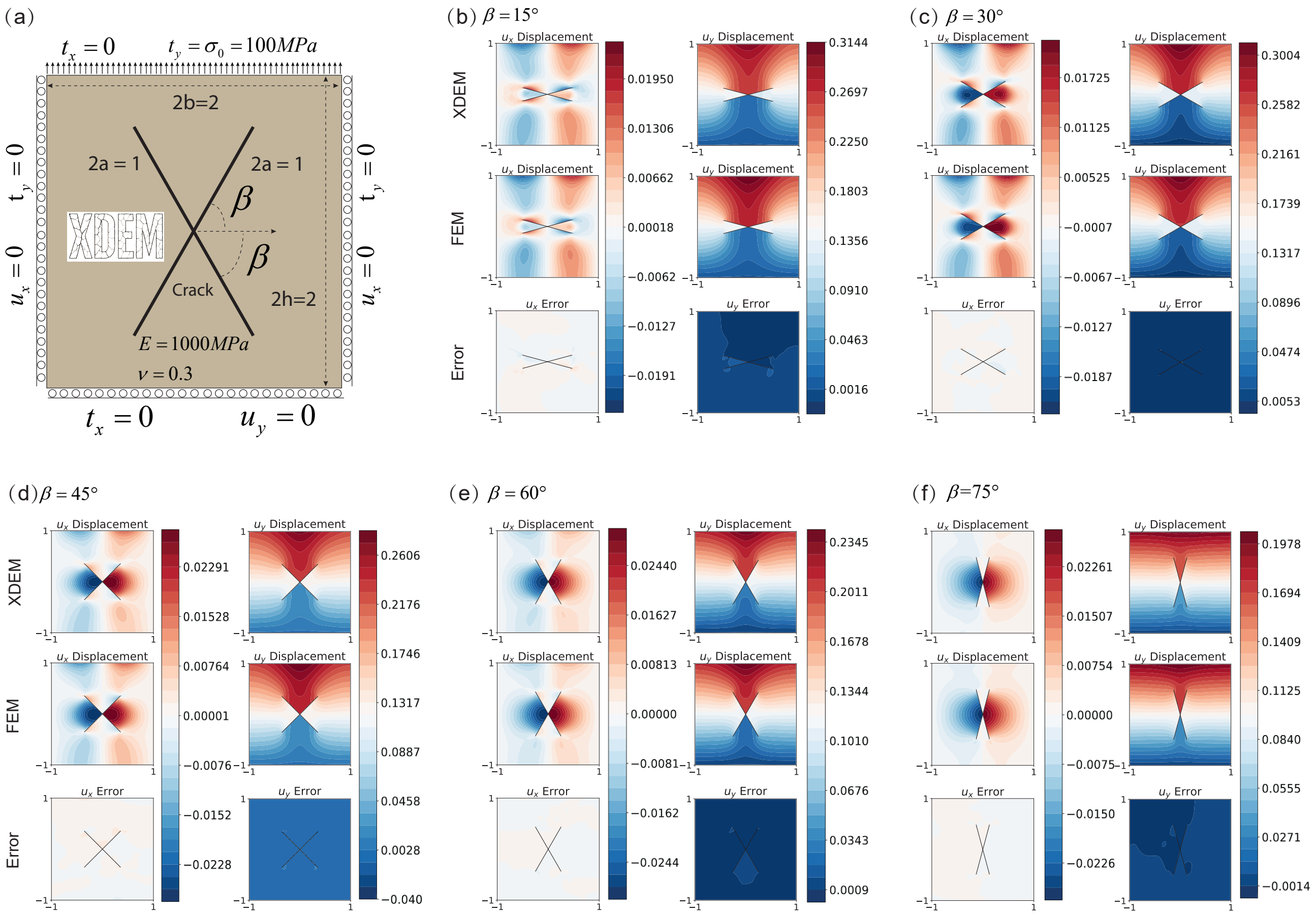

Fig. R1. Displacement contours predicted by XDEM and FEM (reference solution) for intersecting cracks, demonstrating accurate capture of displacement discontinuities by XDEM.

Discrete crack model in fracture mechanics

The discrete crack model is essentially governed by the PDEs of linear elasticity:

$$
\begin{cases}
\sigma_{ij,j}(\boldsymbol{x}) + f_i(\boldsymbol{x}) = 0 & \boldsymbol{x} \in \Omega, \\
\sigma_{ij}(\boldsymbol{x}) = C_{ijkl}\varepsilon_{kl}(\boldsymbol{x}) & \boldsymbol{x} \in \Omega, \\
\varepsilon_{ij}(\boldsymbol{x}) = \frac{1}{2}(u_{i,j}(\boldsymbol{x}) + u_{j,i}(\boldsymbol{x})) & \boldsymbol{x} \in \Omega \setminus \Gamma^c, \\
u_i^+(\boldsymbol{x}) \not\equiv u_i^-(\boldsymbol{x}) & \boldsymbol{x} \in \Gamma^c, \\
\sigma_{ij}^{\pm} n_j^{\pm} = \bar{q}_i^{\pm} & \boldsymbol{x} \in \Gamma^c, \\
\sigma_{ij}(\boldsymbol{x}) n_j = \bar{t}_i(\boldsymbol{x}) & \boldsymbol{x} \in \Gamma^{\boldsymbol{t}}, \\
u_i(\boldsymbol{x}) = \bar{u}_i(\boldsymbol{x}) & \boldsymbol{x} \in \Gamma^{\boldsymbol{u}}.
\end{cases}
\tag{1}
$$

Here, $\boldsymbol{\sigma}$, $\boldsymbol{C}$, $\boldsymbol{\varepsilon}$, and $\boldsymbol{u}$ denote the stress tensor, stiffness tensor, strain tensor, and displacement vector, respectively. The domain is Ω, with $\Gamma^{\boldsymbol{u}}$, $\Gamma^{\boldsymbol{t}}$, and Γ^c representing the Dirichlet boundary, Neumann boundary, and crack surface, respectively. $\boldsymbol{f}$, $\bar{\boldsymbol{t}}$, and

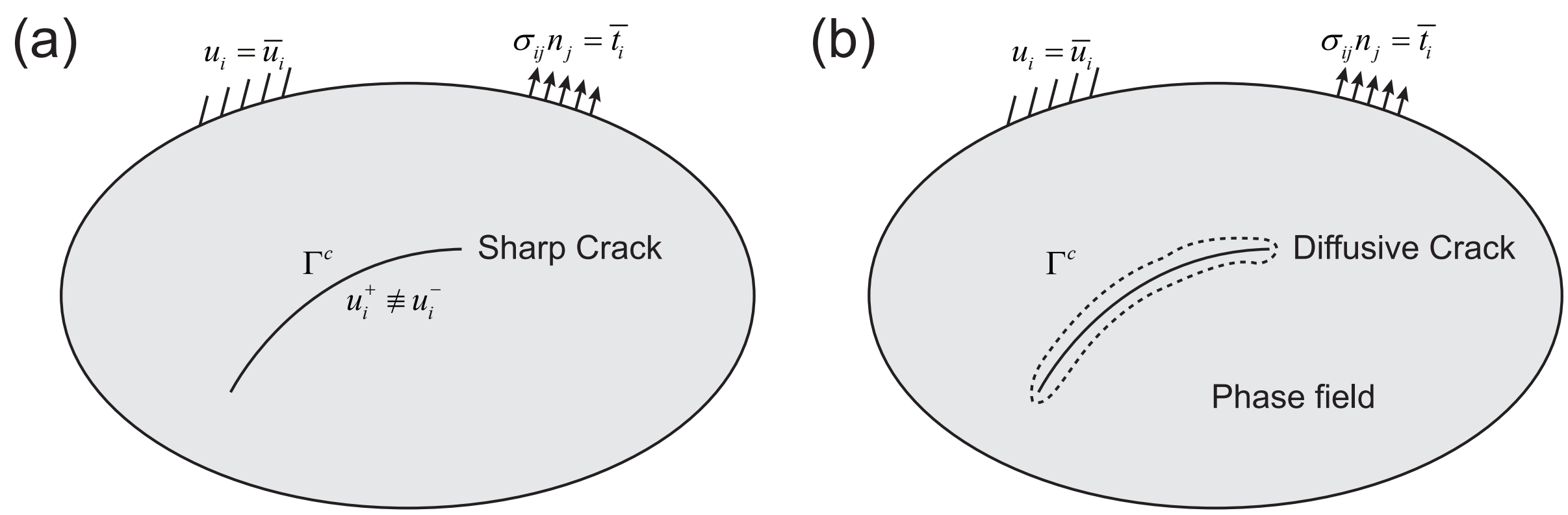

Fig. R2. Schematic of discrete and continuous damage models in fracture mechanics: (a) Discrete crack model with a sharp crack; (b) Continuous damage model where the sharp crack is regularized into a diffused zone.

$\bar{\boldsymbol{u}}$ denote the body force, prescribed traction, and prescribed displacement. $\bar{\boldsymbol{q}}$ denotes the external traction vector applied on the crack surface. The superscripts $\pm$ indicate the two opposing sides of the crack. The symbol $\not\equiv$ indicates a displacement discontinuity across Γ^c. In this work, only Dirichlet and Neumann boundary conditions are considered, with traction-free conditions $\bar{\boldsymbol{q}} = 0$ assumed on Γ^c. The boundaries satisfy $\Gamma^{\boldsymbol{t}} \cup \Gamma^{\boldsymbol{u}} = \Gamma$ and $\Gamma^{\boldsymbol{t}} \cap \Gamma^{\boldsymbol{u}} = \emptyset$. A schematic of the discrete crack model is shown in Fig. R2a.

The system of equations Eq. (1) can often be reformulated by the principle of minimum potential energy:

$$
\begin{aligned}
&\boldsymbol{u} = \arg\min_{\boldsymbol{u}} \Pi, \\
&\Pi = U_e - W_{ext}, \\
&U_e = \int_{\Omega} \frac{1}{2}\, \varepsilon(\boldsymbol{x}) : \boldsymbol{C} : \varepsilon(\boldsymbol{x})\, dV, \\
&W_{ext} = \int_{\Omega} \boldsymbol{f} \cdot \boldsymbol{u}(\boldsymbol{x})\, dV + \int_{\Gamma^{\boldsymbol{t}}} \bar{\boldsymbol{t}} \cdot \boldsymbol{u}\, dS + \int_{\Gamma^{\boldsymbol{c}}} \bar{\boldsymbol{q}}^{+} \cdot \boldsymbol{u}^{+} dS + \int_{\Gamma^{\boldsymbol{c}}} \bar{\boldsymbol{q}}^{-} \cdot \boldsymbol{u}^{-} dS, \\
\text{s.t. } & u_i(\boldsymbol{x}) = \bar{u}_i(\boldsymbol{x}),\ \boldsymbol{x} \in \Gamma^{\boldsymbol{u}}; \quad \forall\ u_i^{+}(\boldsymbol{x}) \not\equiv u_i^{-}(\boldsymbol{x}),\ \boldsymbol{x} \in \Gamma^{c}.
\end{aligned}
\tag{2}
$$

It is straightforward to show that $\delta\Pi = 0$ is equivalent to Eq. (1). By solving Eq. (2), the displacement field can be obtained, from which the stress field follows via the constitutive and geometric relations. To further simulate crack propagation, a fracture criterion must be specified. Common criteria include the maximum circumferential stress criterion [3], the maximum energy release rate criterion [4], and the minimum strain energy density criterion [5]. In this work, we adopt the maximum circumferential stress criterion, whereby the crack propagates in the direction of the maximum hoop tensile stress. $\cdots$"

The Reviewer's Comment #5

5. In Table S1, the functions w, g, X_1, X_2 are not presented.

Response:

Thanks for your comments. Added:

Table R1
Summary of the main symbols and notation used in this work.

Notation	Description
$\boldsymbol{u}$	Displacement field
ε	Strain field
$\boldsymbol{\sigma}$	Stress field
$\boldsymbol{C}$	Elastic stiffness tensor
$\boldsymbol{f}$	Body force
ϕ	Phase field variable
Γ^c	Crack surface
λ	Lamé constant
E	Young's modulus
ν	Poisson's ratio
G	Shear modulus
K	Bulk modulus
G_c	Fracture energy release rate
l	Regularization (length scale) parameter
K_I, K_{II}, K_{III}	Mode I, II, and III stress intensity factors
J	J-integral
$\Psi^+(\boldsymbol{\varepsilon})$	Tensile part of the strain energy density
$\Psi^-(\boldsymbol{\varepsilon})$	Compressive part of the strain energy density
$w(\phi)$	Degradation function that represents the reduction of material stiffness
$g(\phi)$	Local dissipation function
$\boldsymbol{X}$	Learnable Williams series expansion in the extend function
$D(\boldsymbol{x})$	Distance function
T	Decay function
FEM	Finite Element Method
XDEM-D	Extended Deep Energy Method in discrete crack models
XDEM-C	Extended Deep Energy Method in continuous damage models

The Reviewer's Comment #6

6. Please comment on the reason for using two different types of neural network architectures, namely KAN and RBF, for the displacement and phase-field, respectively.

Response:

Thanks for your comment. We have provided a detailed explanation in Supplement Section 3.2. In summary, in the continuous damage formulation, since the phase field is more compatible with the functional space of radial basis functions (RBFs), we recommend approximating the phase field using an RBF network. On the other hand, as the displacement field exhibits strong variations near crack tips, and Kolmogorov–Arnold Networks (KANs) have been shown to outperform MLPs for problems with sharp variations [6], we recommend approximating the displacement field with a KAN. The revised Supplement Section 3.2 is shown as follows:

"...

Extended Deep Energy Method in continuous damage models

In the continuous damage formulation, XDEM introduces a phase-field variable to represent cracks. The displacement field and the phase field are approximated by separate neural networks. Since the phase field is more compatible with the functional space of radial basis functions (RBFs), we recommend approximating the phase field using an RBF network, with a detailed justification provided in Section S4.5. On the other hand, as the displacement field exhibits strong variations near crack tips, and Kolmogorov–Arnold Networks (KANs) have been shown to perform better than MLPs for problems with sharp variations [6], we recommend approximating the displacement field with a KAN. ···"

The Reviewer's Comment #7

7. In Fig. 12b, XDEM-C indeed accurately captures the failure displacement, but it appears to be less accurate for the peak force.

Response:

Thanks for your comment. We noticed that the accuracy of XDEM-C shows a discrepancy compared with the traditional FEM results in 3D. Therefore, we carefully re-examined the implementation and repeated the 3D experiments. The updated results are presented as follows:

"··· The load–displacement response is shown in Fig. R3b. It is worth noting that conventional DEM can only solve this problem if collocation points are heavily concentrated around the crack; with uniformly distributed points, DEM fails to converge. In contrast, XDEM achieves accurate results using far fewer, uniformly distributed collocation points. Although the accuracy of XDEM-C still shows some discrepancy compared to traditional FEM, to the best of our knowledge, the 3D load-displacement curve is the best result available in the current literature. We believe that XDEM-C still has room for improvement in the future, especially regarding the issue of overcoming the energy multiminima barrier in phase-field fracture, which is a key challenge for the future breakthroughs of XDEM-C.

..."

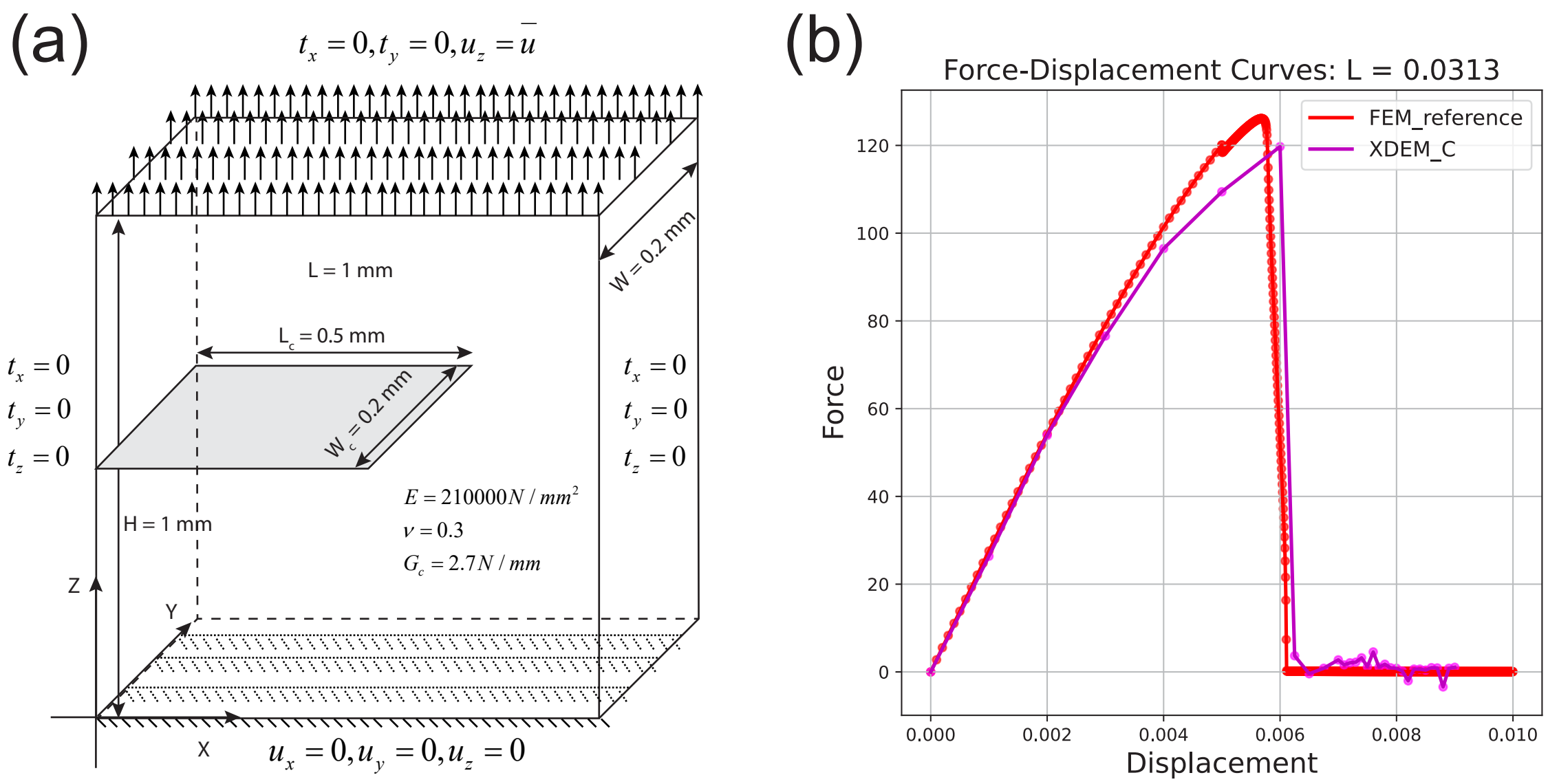

Fig. R3. (a) Geometry of the 3D crack problem. (b) Load–displacement response predicted by XDEM.

Response:

Finally, thank you once again for your thorough and high-quality review.

Reviewer #2

The Reviewer's Comment

The paper presents XDEM, essentially an extended DEM for fracture mechanics. XDEM is in spirit like a classical XFEM enrichment, i.e., it explicitly introduces a crack functon(like a Heaviside in XFEM) and a a Williams-series-based extended functon(analogous to crack-tip asymptotic enrichment). The paper is well written. The computational examples are extensive. While the contribution is interesting and the presentation is generally clear, I have a number of major and minor comments that, in my opinion, need to be addressed to strengthen the manuscript.

Response:

Thank you for your precious time and valuable suggestions. We sincerely appreciate your insightful feedback, which has significantly improved the quality of our work. We have revised the manuscript based on your comments.

The Reviewer's Major Comment #1

1. The quality and size of several figures are insufficient for publication. In particular, figures such as Fig. 2 and Fig. 3 have axis labels, tick marks, legends, and titles that are barely readable at normal viewing scale. This significantly hampers the readers ability to assess the results. All figures should be enlarged and reformatted with substantially larger fonts and improved resolution to ensure clarity in both print and digital formats.

Response:

Thank you for your comment. We have improved the quality of all images in the manuscript, such as Fig 2 and 3. Please see the revised version.

The Reviewer's Major Comment #2

2. In Section S3.3, sequential LoRA transfer is said to potentially induce non-physical fracture patterns (linked to Section S4.1), which are attributed to integration inaccuracies and zero-energy modes. Could the authors clarify how exactly LoRA interacts with this mechanism?

Response:

Thanks for your comment. You have observed and read very carefully. In fact, LoRA is not closely related to integration inaccuracies and zero-energy modes. The issues of integration inaccuracies and zero-energy modes primarily arise due to insufficient integration precision. We have provided a detailed explanation in Supplement Section 3.3:

"··· It should be noted that transfer can be performed incrementally between consecutive load steps. However, in the later stages of displacement-controlled loading, non-physical fracture patterns may appear (see Section S4.1). This issue arises due to the delicate balance between the expressive power of neural networks and the accuracy of numerical integration. Although it is fundamentally unrelated to transfer learning algorithms like LoRA, this issue primarily stems from the numerical integration precision. In the future, however, a transfer learning algorithm that improves the integration accuracy in subsequent load steps could potentially be developed for solving fracture mechanics problems using PINNs. Therefore, in practice, XDEM often uses the parameters obtained from the first load step as the baseline for transfer learning. For the fracture propagation results in the manuscript, we use the parameters obtained from the first load step as the baseline for transfer learning. ···"

The Reviewer's Major Comment #3

3. Additionally, do the reported wall-times and efficiency comparisons correspond to fully sequential LoRA transfer between load increments, or to transfer always initialized from the first load step? This distinction seems important for assessing the robustness and actual computational savings of the proposed strategy. Especially since the code is not online yet.

Response:

Thanks for your comment. For the fracture propagation results in the manuscript, we use the parameters obtained from the first load step as the baseline for transfer learning. We have provided a detailed explanation of this in Supplement Section 3.3, as follows. Additionally, our code has been open-sourced on GitHub, and the link is https://github.com/yizheng-wang/XDEM.

"··· For the fracture propagation results in the manuscript, we use the parameters obtained from the first load step as the baseline for transfer learning. ···"

The Reviewer's Minor Comment #1

1. p.3 "(modes I, II and III).."

Response:

Thanks for your comment. Corrected.

The Reviewer's Minor Comment #2

2. Fig.S23 says "Theretical Minimum"

Response:

Thanks for your comment. Corrected.

The Reviewer's Minor Comment #3

2. there is a superscript in reference 2: Hussain, M., Pu, S. Underwood"1" , J. Strain energy release rate e for a crack under combined mode i and mode ii. In Fracture analysis: Proceedings of the 1973 national symposium on fracture mechanics, part II, vol. 560, 2 (ASTM International, 1974).

Response:

Thanks for your comment. Corrected.

Remarks on code availability

The code is not available.

Response:

Our code has been open-sourced on GitHub, and the link is https://github.com/yizheng-wang/XDEM. Finally, thank you once again for your thorough and high-quality review.

References

[1] L. Zhao, Q. Shao, Denns: Discontinuity-embedded neural networks for fracture mechanics, Computer Methods in Applied Mechanics and Engineering 446 (2025) 118184.

[2] O. Lopez-Pamies, J. E. Dolbow, G. A. Francfort, C. J. Larsen, Classical variational phase-field models cannot predict fracture nucleation, Computer Methods in Applied Mechanics and Engineering 433 (2025) 117520.

[3] F. Erdogan, G. Sih, On the crack extension in plates under plane loading and transverse shear, Journal of basic engineering 85 (4) (1963) 519–525.

[4] M. Hussain, S. Pu, J. Underwood, Strain energy release rate for a crack under combined mode i and mode ii, in: Fracture analysis: Proceedings of the 1973 national symposium on fracture mechanics, part II, Vol. 560, ASTM International, 1974, p. 2.

[5] G. C. Sih, Strain-energy-density factor applied to mixed mode crack problems, International Journal of fracture 10 (3) (1974) 305–321.

[6] Y. Wang, J. Sun, J. Bai, C. Anitescu, M. S. Eshaghi, X. Zhuang, T. Rabczuk, Y. Liu, Kolmogorov–arnold-informed neural network: A physics-informed deep learning framework for solving forward and inverse problems based on kolmogorov–arnold networks, Computer Methods in Applied Mechanics and Engineering 433 (2025) 117518.
